# seqQscorer: automated quality control of next-generation sequencing data using machine learning

**DOI:** 10.1186/s13059-021-02294-2

**Published:** 2021-03-05

**Authors:** Steffen Albrecht, Maximilian Sprang, Miguel A. Andrade-Navarro, Jean-Fred Fontaine

**Affiliations:** Johannes Gutenberg-Universität Mainz, Biozentrum I, Hans-Dieter-Hüsch-Weg 15, 55128 Mainz, Germany

**Keywords:** Next-generation sequencing data, Quality control, Machine learning, Classification, Bioinformatics

## Abstract

Controlling quality of next-generation sequencing (NGS) data files is a necessary but complex task. To address this problem, we statistically characterize common NGS quality features and develop a novel quality control procedure involving tree-based and deep learning classification algorithms. Predictive models, validated on internal and external functional genomics datasets, are to some extent generalizable to data from unseen species. The derived statistical guidelines and predictive models represent a valuable resource for users of NGS data to better understand quality issues and perform automatic quality control. Our guidelines and software are available at https://github.com/salbrec/seqQscorer.

**Supplementary Information:**

The online version contains supplementary material available at 10.1186/s13059-021-02294-2.

## Background

Functional genomics based on next-generation sequencing (NGS) technology is used to study regulatory elements in genomes of all types of species. It is widely used in biological and clinical applications thanks to a variety of existing complementary assays that allow the investigation of, for example, gene expression quantification (RNA-seq), epigenetic modification and transcription factor occupancy (ChIP-seq), and open chromatin regions (e.g., DNase-seq, MNase-seq, or ATAC-seq).

The analysis of NGS data requires a stepwise process handled by dedicated software tools. The first processing step is the quality control (QC) of the data. It is of crucial importance to filter out low-quality data files that would have a negative impact on downstream analyses through addition of noise or systematic bias to the analyzed dataset [1]. When deriving differences between groups of samples, low-quality samples would increase the variance within a group and thus hamper the ability of statistical tests to find significant differences among them. In a clinical context, patient data of unnoticed low-quality can also lead to wrong diagnosis or ill-suited treatment. Filtering out or editing a small portion of sequencing reads within a file or applying more sophisticated bias mitigation methods may be detrimental to the downstream analysis [2, 3] or may not be enough to correct such a systematic error [4]. Common QC tools analyze the data files to derive numerous highly specific quality features for manual review. As the usefulness of many of these features was never demonstrated, a large majority of NGS scientists is still not confident about classifying a sequencing file by quality.

QC tools, used at the first step of an NGS pipeline, analyze raw sequencing data. The raw data is stored in FastQ files containing a set of short strings of DNA sequences (e.g., 100 bases long) called reads and related information such as a quality score for each base reflecting its sequencing error probability. The most popular QC tool in the NGS community is FastQC [5]. It performs various analyses that could indicate problems such as position-dependent biases (“Per base sequence quality” analysis), sequencing adapter contamination (“Overrepresented sequences” analysis), or DNA over amplification (“Sequence duplication levels” analysis). Quality analyses based on the raw data can be complemented by analyses performed at later steps of the NGS data processing. An important subsequent step is the mapping of the reads to the reference genome if available. Dedicated software tools such as Bowtie2 output mapping statistics that could be used as indicators of quality [6–9]. The number of sequencing reads that map to a unique position or the number of reads that do not map in the reference genome are presumably very important quality features. In following data processing steps, related software tools are often assay-specific [9–13]. Their results could still complement the tools mentioned above for different assays. Analyzing the genomic location of the reads to know if they map predominantly to expected functional elements or to know the distribution of the reads near gene transcription start sites (TSSs) are of special interest to ChIP-seq, DNase-seq, or ATAC-seq data for example. Although some tools offer reports that integrate results from several QC software [14, 15], the final QC decision still remains manual. This decision is complex given the multiplicity of quality features generated at different steps of the data processing, their expected dependency from experimental conditions (e.g., species, assays, or treatments), and the lack of statistical studies that would recommend specific values that differentiate low- and high-quality data. Therefore, a system to aid NGS QC-related decisions, making them automated and independent of human biases, is desirable.

Due to the coordinated efforts of many research groups, large repositories have been created that collect NGS data files in order to make them available to the scientific community. The scope of some repositories such as GEO and ArrayExpress is to share data with a minimal amount of annotation describing experimental conditions. Annotations are created in accordance with detailed guidelines [16] and ontologies [17]. Although they help to maintain a high standard of data annotation, they do not control the quality of the deposited data. Other specialized repositories, such as TCGA for cancer data, focus on high-quality data. Uniquely, the ENCODE repository [18, 19], specialized in functional genomics, provides access to a large number of high- and low-quality NGS files that were labeled either as released or revoked, respectively, based on a semi-automatic QC procedure. Although ENCODE guidelines were created to help NGS specialists to produce data of high quality, curators of their repository still manually decide the quality of the files after reviewing various quality features [20, 21]. The goal of this study is to improve NGS QC procedures by comparing these files and applying statistical methods and machine learning algorithms to derive useful statistics and classification models leveraging comprehensive quality features.

Although machine learning has been used to classify the quality of reads or single-nucleotide polymorphisms [22], a high performing application to full NGS files is still required. We focused our work on RNA-seq, ChIP-seq, and DNase-seq data files of human and mouse samples, and included ATAC-seq data in the validations. We were first interested in defining the scope of application and relevance of each individual feature by comparing data statistics from different species and assays. Then, we used machine learning methods to derive optimal and unbiased predictive models combining multiple features. We evaluated decision-tree based ensemble methods, a multilayer perceptron as well as Bayesian, instance-based, kernel-based, and regression-based classifiers.

After extensive validation of the models, we investigated their usefulness in different scenarios such as database curation and disease diagnosis. Finally, we released to the community our statistical guidelines and a software application to use the classification models on new datasets.

## Results

We have studied a large number of annotated NGS files to characterize data quality and to create machine learning models able to automatically predict quality from the raw data. Our goal is to provide an alternative to manual quality control of NGS data files, which currently requires high-level expertise and highly depends on assays and experimental conditions and is prone to human biases. Interestingly, a minimum number of uniquely mapped reads and usable fragments mentioned in the ENCODE guidelines cannot be used to categorize the ENCODE data with respect to quality (Additional file 1: Fig. S14 A, C, and E).

### Workflow

**Fig. 1:**
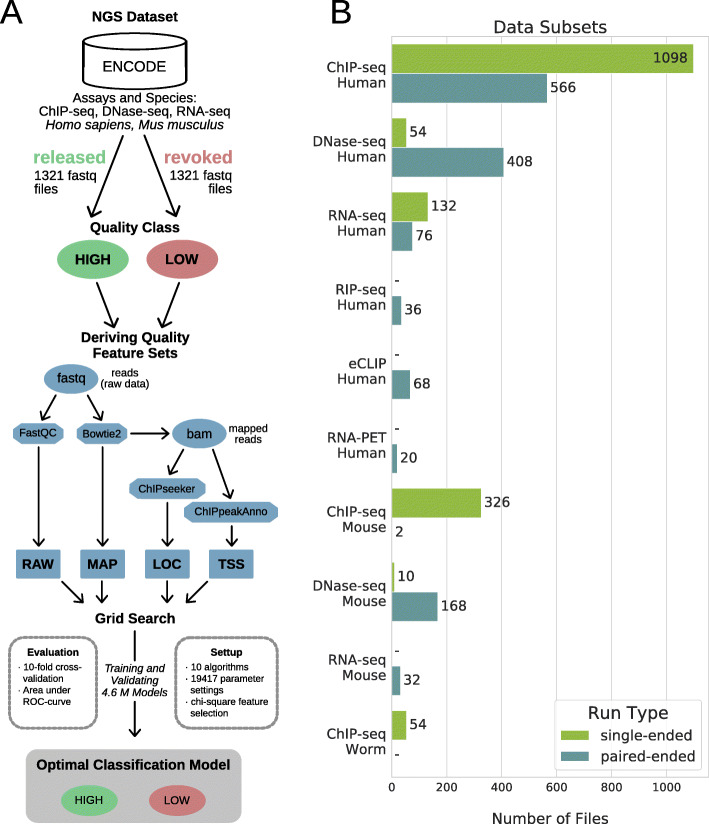
Workflow and dataset. **a** Workflow. Raw NGS data files were retrieved from the ENCODE data portal. Four quality feature sets were derived using standard bioinformatics tools (e.g., FastQC): raw data (RAW), genome mapping (MAP), genomic localization (LOC), and transcription start sites profile (TSS) feature sets. A grid search was used to derive optimal machine learning models by testing various parameter settings and algorithms. **b** Dataset. Bars show the number of files in each data subset (*y*-axis). Two files are counted for each paired-end sample

Our study is based on 2642 human and mouse raw NGS data files labeled as high- or low-quality from the ENCODE data portal (Fig. 1). From them, we extracted different quality features using software tools commonly used in the NGS field. Each tool represents a different stage or view of the NGS data analysis, providing features sets related to raw sequencing reads (RAW), mapping to a reference genome (MAP), genomic localizations of the reads (LOC), and spatial distribution of the reads near transcription start sites (TSS) (see the “Methods” section for details).

Our approach was first to derive statistical guidelines from the detailed study of individual quality features, and then to systematically benchmark 10 popular machine learning algorithms in a grid search, to predict the quality of NGS data files based on combinations of quality features and various sets of parameters (see grid search specifications in Additional file 2). We finally evaluated 4.6 M predictive models covering different data subsets: either generic (including all species and/or all assays) or specialized in particular species and assays (e.g., human ChIP-seq or mouse DNase-seq).

### Quality prediction

#### One-feature quality predictions as baseline and guidelines

**Fig. 2:**
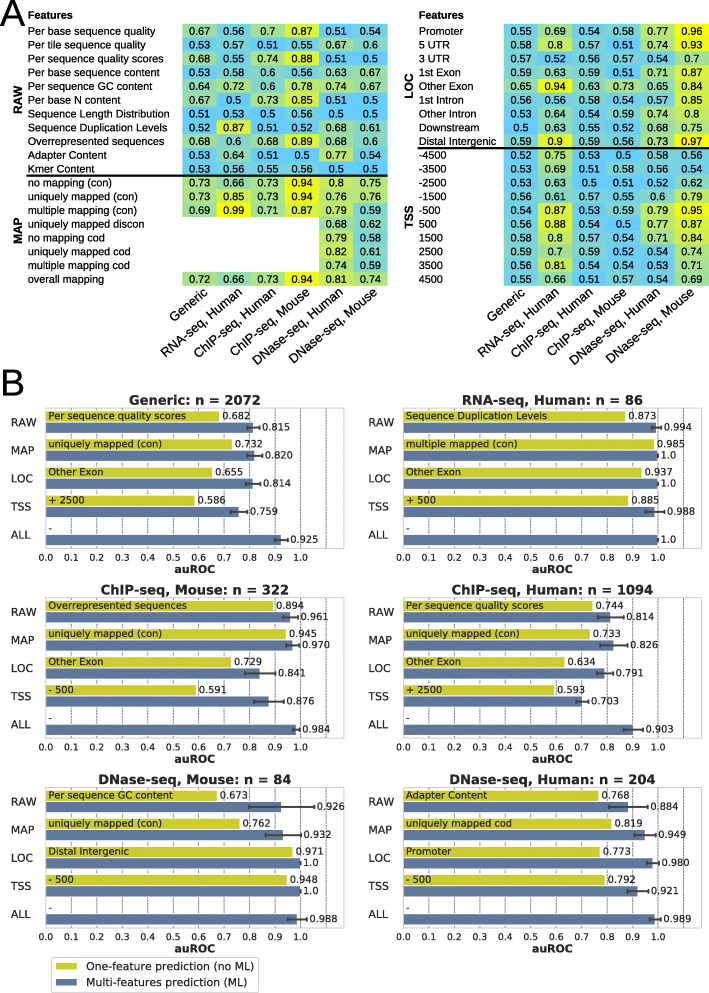
Predictive performance of quality features and machine learning (ML). **a** One-feature predictions. Predictive performances of each feature (no machine learning) as areas under receiver operating characteristics curves (auROC) ranging from 0.5 (random predictions) to 1 (perfect predictions). **b** Multi-feature predictions. Predictive performances of optimal generic and specialized machine learning models trained using given feature sets (*y*-axis) outperform one-feature predictions. Bars show mean value and error bars show standard deviations derived from 10-fold cross-validations within the grid search. Feature sets: RAW (raw data), MAP (genome mapping), LOC (genomic localization), TSS (transcription start sites profile), ALL (all features)

Before evaluating machine learning algorithms, we evaluated the predictive power of each quality feature independently (Fig. 2a). This first analysis, which does not involve machine learning, gave us an overview of their performance across the data subsets and a baseline for the evaluation of the machine learning algorithms. We compared the results using the area under the receiver operating characteristic curve (auROC) that ranges from 0.5 to 1, from not predictive to completely predictive, respectively. The predictive power of the features strongly depends on the data subset, ranging from poorly predictive (e.g. genomic localization in “1st exon” for human RNA-seq; auROC = 0.50) to highly predictive (e.g., “overall mapping” for mouse ChIP-seq files; auROC = 0.94).

Some quality features are of broad interest because of their good performance across all data subsets, especially all MAP features and the following two RAW features: “Overrepresented sequences” and “Per sequence GC content” (auROC up to 0.89 and 0.78, respectively). Other quality features are less interesting because of their poor performance in all subsets (e.g., “Sequence Length Distribution”; or “TSS − 2500”; auROC ≥ 0.62). Comparing species, DNase-seq showed more differences than ChIP-seq results. This is probably due to the large difference in composition between human and mouse DNase-seq samples (Additional file 1: Fig. S7). Analyzing separately narrow and broad peak samples from the ChIP-seq results, we could observe an under-representation of broad peak samples (only 82 human and 46 mouse samples) and differences between peak types (Additional file 1: Fig. S9).

From this analysis, we have derived detailed statistical views that confirm the specificity to data subsets observed above (Additional file 1: Fig. S1). By providing an easy way to compare quality features of new data files to thousands of ENCODE files, these results can serve as statistical guidelines to NGS specialists.

#### Multi-feature quality predictions by machine learning

With the performance of each single quality feature as baseline, we evaluated the potential improvement of using combinations of features by machine learning algorithms to predict NGS data files quality. We evaluated machine learning models trained with the different data subsets for each feature set (Fig. 2b). The models, tuned by a grid search that systematically explores performances over the parameter space, outperformed the baseline. Each model may use a different algorithm or set of parameters. For example, the tuned generic model using all features is based on a Random Forest classifier using 1000 estimators, while the tuned human ChIP-seq model for single-end reads experiments is based on a multilayer perceptron with 2 hidden layers (see Additional file 3 for details about parameters used by each tuned model).

Data files quality from each subset can be predicted with high performance, especially when using all features (auROC > 0.9; Fig. 2b). Within each data subset, the different feature sets led to comparable results, although performances were more variable with RAW features in DNase-seq subsets, and LOC and TSS feature sets were less performing for ChIP-seq subsets. Results for all combinations of feature sets and other performance measures such as area under precision-recall curve, accuracy, or F1 are shown in Additional file 1: Fig. S2 and S5A.

This analysis confirms the expectation that sequencing reads from the ChIP-seq subset (including narrow and broad peak types) would not be highly biased towards specific genomic localizations or TSS relative positions different to RNA-seq, for instance, for which these two feature sets enable very high performing models (auROC = 1 or 0.988, respectively). Nevertheless, we also evaluated separate models for narrow-peak and broad-peak ChIP-seq samples (Additional file 1: Fig. S10). Considering all features, human and mouse narrow-peak models performed similarly to the generic or less specialized ChIP-seq models described above. Broad-peak models outperformed the other models although we could not exclude a bias due to the lower sample size of the underlying training sets.

Taken together, we can see that the tuned generic and specialized models show their best performances mostly when using all quality features. Therefore, we considered those tuned models using all features as optimal and used them for further analyses below (see Additional file 4 for details about parameters of the 7 optimal models).

### Within-experiment analysis

**Fig. 3:**
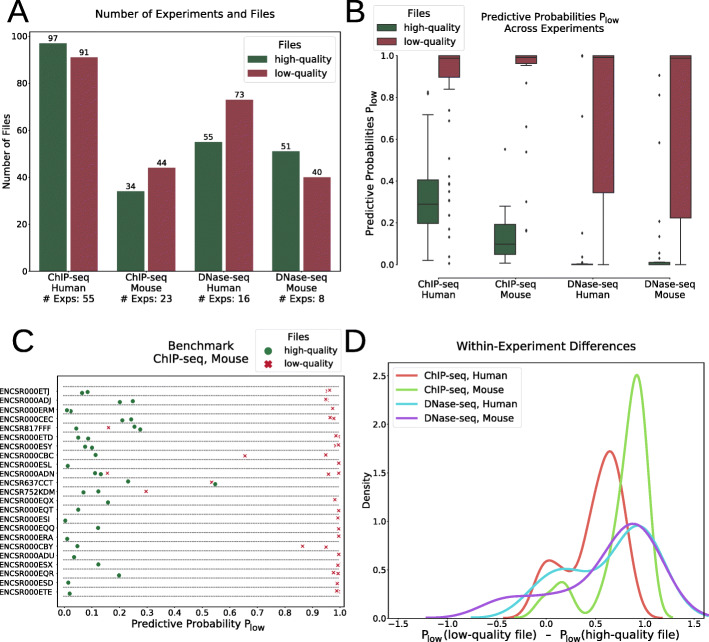
Within-experiment differences. **a** Numbers of selected experiments and files to study within experiment differences. Only experiments that contain released files together with revoked files were selected. **b** Predictive probabilities across experiments summarize the good predictive power in each data subset. **c** Benchmark of mouse ChIP-seq files in selected experiments illustrates the good predictive power of the models. P_low_: predictive probability to be of low quality. **d** Density curves of within-experiment differences between predictive probabilities for low- and high-quality files. Average differences are large (range from 0.5 to 1). The low-quality probabilities P_low_ shown in panels **b**, **c**, and **d** were computed by the optimal specialized models trained on the corresponding data subset but without the files from the experiment under investigation

The extent to which individual NGS researchers would benefit from using machine learning models in their QC procedure could be known by analyzing the results on replicate files produced within the same experiment. In our dataset derived from ENCODE, some experiments gather high- and low-quality files from different replicate and control samples. For the different species-assay combinations we extracted these experiments with the corresponding files (Fig. 3a).

Focusing on the probability of a file to be of low quality that was provided by the optimal specialized models to each file across the different experiments, our models were able to clearly differentiate which files would be considered high or low quality after manual QC (Fig. 3b). Within each experiment, we could mostly observe a clear cut or a meaningful sorting by actual quality (Fig. 3c and Additional file 1: Fig. S3), with an average difference between low- and high-quality files ranging from 0.5 to 1 (Fig. 3d). From these observations, we conclude that the optimal specialized models were not biased towards some experiments. These results suggest that, early in the sequencing analysis pipeline, researchers can already define the potential of their data files to be considered of enough quality for publication and can accordingly take decisions that could save a substantial amount of time and resources for further analyses, storage, or manual reviews. For instance, out of 23 mouse ChIP-seq experiments in our dataset, 41 (53%) files could have been early identified as of low quality and not submitted, stored, processed, and manually reviewed by the ENCODE database curators.

### Top machine learning algorithms and parameters

**Fig. 4:**
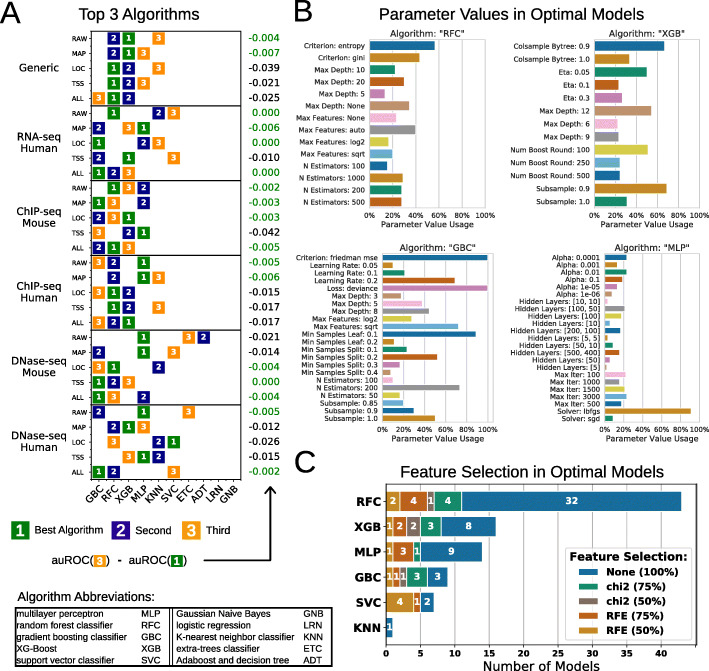
Best performing algorithms and parameters. **a** The top three performing algorithms for selected data subset-feature set combinations (colored boxes). Numbers on the right-hand side show the difference of the area under receiver operating characteristics curve (auROC) between the very best and the third best algorithm. Feature sets: RAW (raw data), MAP (genome mapping), LOC (genomic localization), TSS (transcription start sites profile), ALL (all features). **b** Frequency of algorithmic parameter settings in 90 optimal models selected by the grid search. For each algorithm, optimal models were created for each possible combination of feature set and data subset. **c** Frequency of chi-square-based feature selection (chi2) and recursive feature elimination (RFE) settings in best performing models. For each possible combination of feature set and data subset, we compared optimal models derived by different algorithms and retained the best one and its algorithm

Out of our model tuning strategy, which systematically tested 10 different algorithms and numerous parameter sets, different algorithms could be found in models optimal for each data subset and/or feature set. Given the heterogeneity of the data composed of numerical and categorical values, and the moderate dataset size, we thought that decision-tree-based algorithms would be appropriate to the task. To test this hypothesis, we summarized the results across the data subsets and combinations of feature sets. As expected, decision-tree-based algorithms (random forest, gradient boosting, and XG boost) often performed better than others as well as multilayer perceptron, which is a deep learning classifier based on artificial neural networks (Fig. 4a). In general, there were only minor differences between the top algorithms. Nevertheless, the choice of their parameter values proved to be critical (Fig. 4b). For example, the multilayer perceptron clearly benefited from the quasi-Newton method (lbfgs) to solve the weight optimization (> 80% of the best models) but optimal sizes of the hidden layers were more dependent on other parameters. The choice of an automatic feature selection could also be of importance, especially with gradient boosting algorithms or support vector machines (Fig. 4c).

### Cross-species generalization

**Fig. 5:**
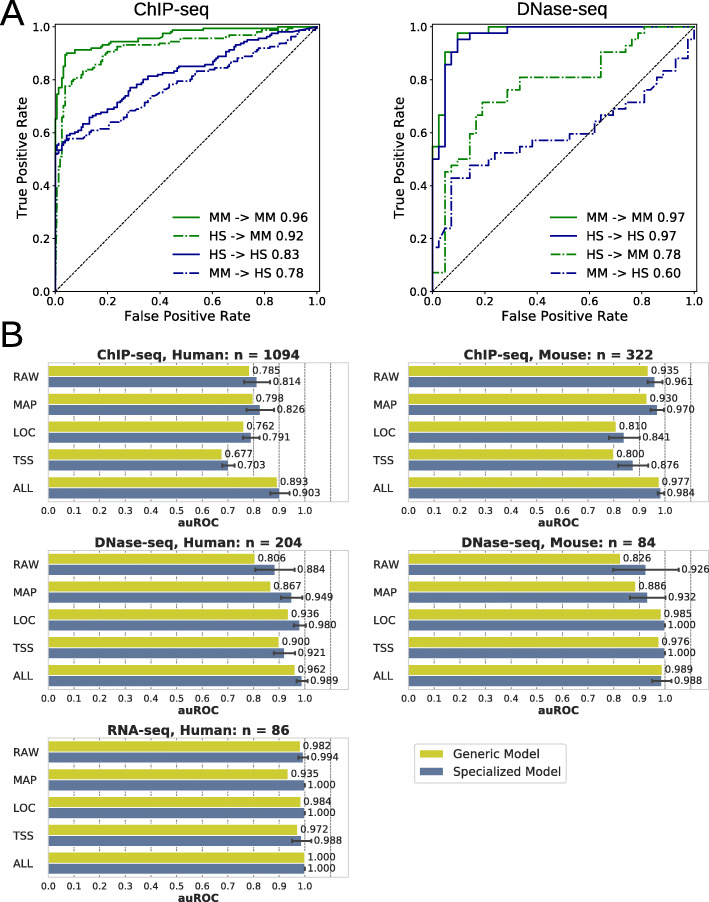
Unbiased and generalizable models. **a** Performance of species-specific models (optimal specialized models) in cross-species predictions demonstrates model generalization to other species. ROC curves show the classification performance for different species-assay combinations using all features. The solid lines represent cases in which data from the same species was used to define training and testing sets. The dashed lines show the performance on cases in which the species defining the training data differs from the species defining the testing data; e.g., “HS → MM” means the training set contains only data from human (*Homo sapiens*, HS), while the testing data is only from mouse (*Mus musculus*, MM). Legends also show corresponding auROC values. **b** Correlation of predictive performance of the optimal generic model trained on all data and features compared to different specialized models demonstrates lack of bias. For each feature set, performance of the generic model is detailed across the data subsets (green bars) and compared to specialized models trained for each combination of feature set and data subset (blue bars). Error bars show standard deviations derived from 10-fold cross-validations within the grid search. Feature sets: RAW (raw data), MAP (genome mapping), LOC (genomic localization), TSS (transcription start sites profile), ALL (all features). ROC, receiver operating characteristics; auROC, area under ROC curve

A main limitation of this study is the availability of labeled data only for human and mouse. In order to know if the models were potentially generalizable to other species, we conducted a test where models were trained with human data and tested on mouse data unseen during model training, and vice versa (Fig. 5a). The tests were performed with ChIP-seq and DNase-seq data, which was available for both species. Results showed that ChIP-seq models trained with a particular species can predict the quality of files from the same and the other unseen species with comparable performance. This could not be clearly observed with DNase-seq, for which prediction performance dropped substantially when trying to predict the file quality from data of unseen species. ROC-curves derived from the different feature sets were consistent with this observation (Additional file 1: Fig. S4). Therefore, cross-species generalizability of the models can be considered assay dependent.

### Generic model

A model that was of importance to us was the optimal generic model trained on files from different species and different assays of the full dataset. Although its performance was high during the cross-validations (Fig. 2b) we were interested to know if it could be biased to data files from a particular subset, especially because the human ChIP-seq subset is overrepresented (52% of the dataset). After tuning and cross-validation of the generic model, we detailed its performance for each data subset (Fig. 5b and Additional file 1: Fig. S5B). The generic model was able to predict the quality of files from each subset with high performance. Still, specialized models, specifically trained on each subset, performed slightly better. We compared the predictions across different ChIP-seq protein targets; we were unable to observe any bias towards particular targets or an effect of including LOC and TSS features in the model (Additional file 1: Fig. S11). Moreover, we could observe consistency of the predictions with the real annotations when observing distributions of uniquely mapped reads and usable fragments in relation to the ENCODE guidelines (Additional file 1: Fig. S14 A-F).

### Evaluation in independent datasets

We have shown above that machine learning models trained on appropriate data from the same source (ENCODE) are powerful and unbiased predictors of NGS file quality. Testing the models on independent datasets from another source, such as the GEO [23] and Cistrome [24] databases, would allow us to assess the generalizability of our approach and consequently demonstrate its useability in different applications.

#### Application to independent diagnostic studies

**Fig. 6:**
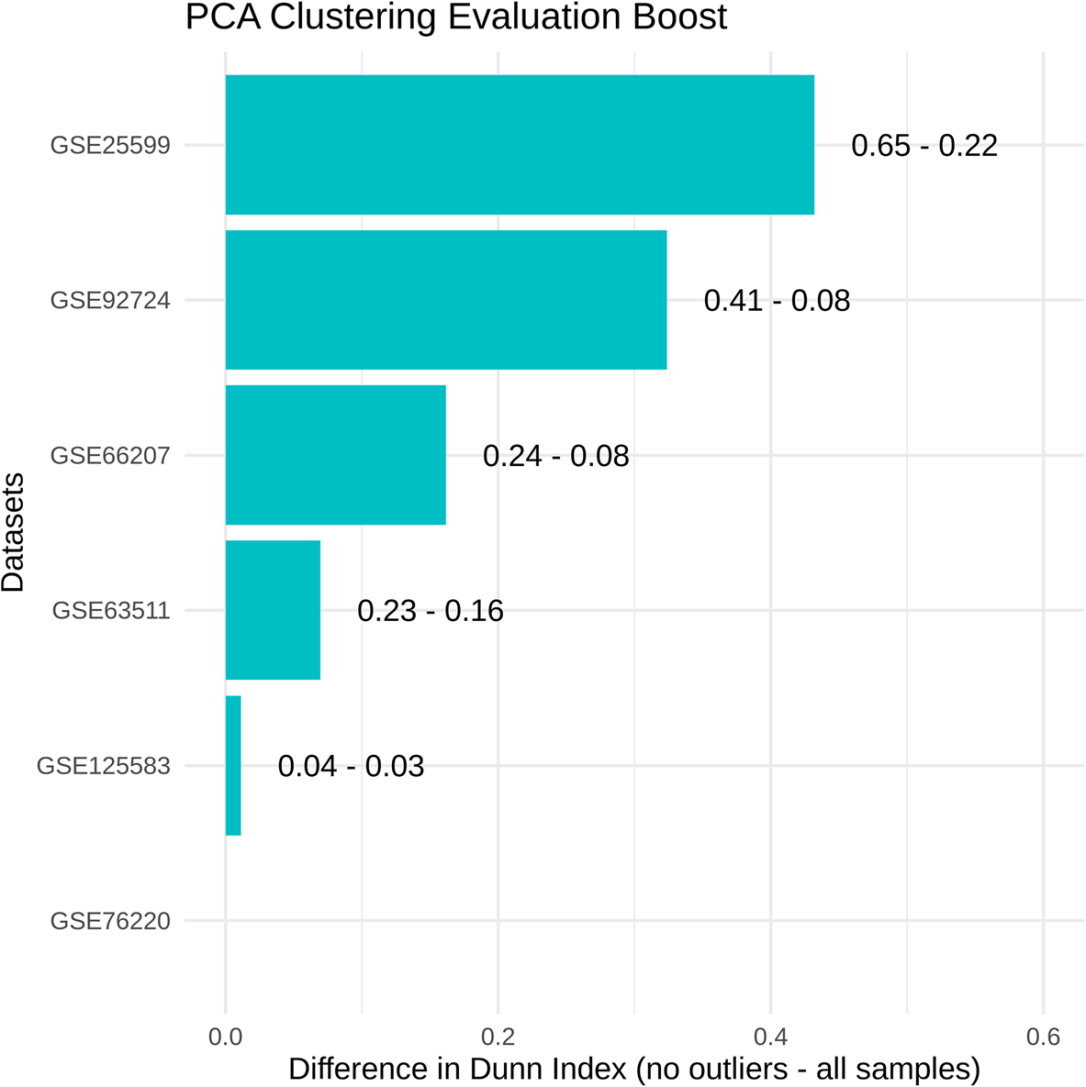
Identifying outliers in independent diagnostic studies. Gene expression data of 90 human disease samples (RNA-seq) were retrieved from 6 independent diagnostic datasets in the GEO database. For each dataset, samples were plotted using the 2 first components of a principal component analysis (PCA) applied on normalized gene expression profiles and clustering by group (control vs disease samples) was evaluated by the Dunn index. The bar plot shows for each dataset the difference of Dunn index before and after automatic removal of outlier samples. In each group, 2 samples with highest low-quality probability were arbitrarily considered outliers. A positive difference in Dunn index denotes an improved clustering after removing outlier samples

The potential of the models to filter low-quality files from diagnostic studies was evaluated on 90 samples from 6 independent gene expression studies related to the following diseases: Alzheimer’s disease (GEO Series GSE125583), Crohn’s disease (GSE66207), diabetes (GSE92724), sporadic amyotrophic lateral sclerosis (GSE76220), liver cancer (GSE25599), and thyroid papillary carcinoma (GSE63511). Assuming a large effect of the quality on gene expression data, being able to automatically identify files of low quality would potentially prevent patients from receiving inappropriate medications or treatments. We analyzed the datasets to mark samples predicted to have low quality as potential outliers (Fig. 6 and Additional file 1: Fig.S12). Those samples could be expected to confuse the comparison of the samples by group, for example, in a two-dimensional projection derived from the gene expression profiles. Thus, we applied a principal component analysis (PCA) before and after the removal of outliers and compared the resulting clusters based on the first two principal components. The clustering of the samples was substantially improved in 4 datasets out of 6 (minor or no improvement in the 2 other datasets). Although selected here automatically and only for testing purposes, such samples could be potential outliers that should be reviewed to decide their relevance for downstream analysis. As we have observed that probabilities returned by the models may not be directly comparable between studies, we would recommend for a similar application to train the models on mostly similar clinical data labeled as low and high quality when available.

#### Assessing the generalizability of models on independent datasets

The Cistrome database provides quality information for independent ChIP-Seq, DNase-Seq, and ATAC-Seq samples that were not in ENCODE and thus not used to train our models. Compared to the quality status used in ENCODE to label our training sets, the Cistrome quality metrics are automatically produced and simple cutoff values are given in the Cistrome’s guidelines to flag each metric to represent good or bad samples. Nevertheless, we used this data to test if our models would generalize to guidelines derived by another database and to ATAC-Seq samples.

**Fig. 7:**
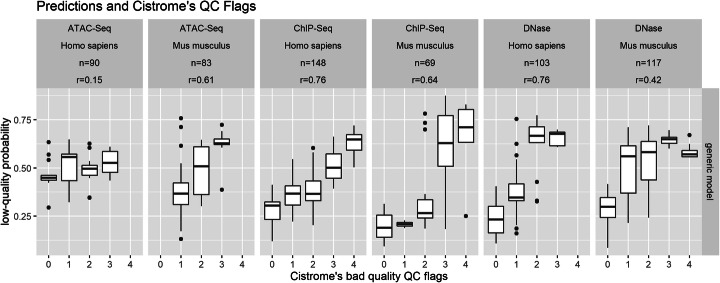
Independent validation on Cistrome datasets. We derived low-quality probabilities on human and mouse ATAC-Seq, ChIP-seq, and DNase-seq datasets referenced in the independent Cistrome database using the optimal generic model. Probabilities of 610 samples (from 32 datasets; not used to train the model) are compared to bad quality flags derived from Cistrome’s guidelines. The higher the number of bad quality flags, the lower the expected quality. Although trained on a binary categorization of independent ENCODE samples as low or high quality, the model’s low-quality probabilities positively correlate with the number of bad-quality flags from Cistrome. *n*, number of samples; *r*, Pearson’s correlation coefficient

We downloaded and analyzed 610 ChIP-Seq, DNase-Seq, and ATAC-Seq samples from 32 datasets annotated in Cistrome. For human and mouse ChIP-seq and DNase-seq subsets, there was a high positive correlation (from 0.42 to 0.76) between the number of Cistrome’s bad quality flags and the low-quality probabilities derived by the optimal generic model (Fig. 7). Similar correlation results were obtained with optimal specialized models used where appropriate, but more variance could be observed (Additional file 1: Fig. S13).

On the ATAC-Seq data, the generic model also produced probabilities that positively correlated with the flags. However, the correlation was high with mouse data (0.61) and only moderate with human data (0.15). Applying the specialized DNase-seq models to ATAC-seq data also performed well with mouse data (0.44) and substantially improved the results for human data (0.46). Although this is not an external or independent validation, we note that a Random Forest model directly trained on ATAC-seq data showed high performance in 10-fold cross-validations (auROC of 0.910 and auPRC of 0.932; see the “Methods” section).

Taken these results together, the optimal generic model better generalizes to independent datasets representing various data subsets. ATAC-seq data could also be analyzed by this model but optimal specialized models for DNase-seq or custom models directly trained on ATAC-seq data may be more relevant.

## Discussion

The versatility and power of NGS applications make the sequencing technology a popular tool in biology and medicine. The complexity to evaluate data quality leads to non-optimal data file filtering and consequently has a negative impact on research and clinical results. Using thousands of files from ENCODE, we first derived statistics-based guidelines to interpret NGS quality features from standard software tools. Then, using a systematic method testing 10 algorithms, different sets of features, and numerous parameters, we were able to build unbiased optimal models able to accurately predict the quality of NGS data files. The classification models outperformed the baseline (the best single feature) and were applicable to other assays and species. The application of this method on labeled database content and external datasets was very promising in its ability to clearly identify problematic samples.

The study of quality features derived by widely used bioinformatic tools on the ENCODE files provides statistic-based guidelines to NGS specialists who have to make a manual decision of high complexity on the quality of their files (Additional file 1: Fig. S1). For instance, FastQC is the most widely used tool to decide the quality of all types of NGS assays. Yet, taken independently, its derived quality features show poor or moderate performance in predicting RNA-seq or DNAse-seq file quality, some of the features being not informative in any situation. On the contrary, other features and tools are more recommendable and, thanks to our data-driven guidelines, NGS specialists will know exactly if their files are more comparable to high- or low-quality files from ENCODE.

The best individual features were systematically outperformed by machine learning models that combine multiple features. In general, across our grid search, the best performing classifiers were tree-based ensemble methods and multilayer perceptron. Such classifiers, especially from the field of deep learning, benefit from bigger datasets but we were not able to find a data repository comparable to our main source (ENCODE) for integration. The restricted amount of annotated low-quality data in ENCODE was therefore the main limitation of our study that could have generated biases and non-generalizable models. Our models have been trained only on two species and three assays for which we found a reasonable number of files. Interestingly, the generalization across different species of some models partly overcomes this limitation. In systematic evaluations, we could still identify biases for some specialized models when sample compositions differ widely between training data and test set. However, we found no biases towards data subsets or experiments when using the optimal generic model based on Random Forest and trained on all data types and all features. This model also generalized better to external datasets, including human and mouse RNA-seq, ChIP-seq, DNase-Seq, and even ATAC-Seq data, a type of assay that had not been used in the ENCODE training set. The inclusion of various types of assays in the training set seemed critical for generalization.

In general, models may also benefit from additional features from other NGS software tools or genomic annotations [25, 26]. Still, results from our models significantly correlated with independent guidelines from Cistrome including assay-specific quality features not used for training. This result highlights the informativeness of the carefully selected set of features used to train our models. This does not mean that other less informative features might not be useful as they could be more interpretable for humans.

As it has been noted in other contexts, negative results can be valuable [27], and we encourage researchers that generate new data to share negative results or low-quality NGS files with the community together with high-quality files in order to enable more accurate and more generalizable models for NGS quality control.

The possible applications detailed in this study highlight the usefulness of our predictive models. Either as a researcher or as a database curator who wishes to identify low-quality files, using the models as a decision support tool can save a substantial amount of resources (up to 50% for problematic experiments). For selected external disease datasets, models trained with ENCODE data have shown their relevance by classifying potential outlier samples. Nevertheless, as explained above, we would highly recommend to directly train models on most similar clinical data to create the best possible models that will prevent the impact of quality issues to diagnosis and therefore to enable patients to receive appropriate medication or treatment. Implemented calibration methods may also be used to better compare results from different models [28]. Yet, we could expect that a technical batch effect would prevent the comparison of the results of the models between datasets, especially with RNA-seq datasets. In such a case, it would still be possible to compare probabilities within a batch, but the comparison between batches could be done using ranks of probabilities. Results obtained on samples undergoing major DNA damage or rearrangements, induced by particular cancer cells, for example, should be taken carefully as models would be limited to features depending on a healthy reference genome and may confuse these phenomena with quality issues.

Finally, the software used to create and apply the models is freely available online either in a github repository or as a docker image (see [29]). Using command line Python scripts, quality features can be computed from user-provided FastQ files and the optimal models can be applied on the derived quality features. The software can be used with data from any species for which a GTF gene annotation file, a corresponding Bowtie index, and training set files can be provided. Together with the optimal generic model, optimal specialized models are provided for human and mouse RNA-seq, ChIP-seq, and DNase-Seq data. Models can be selected for best performance (highest auROC) or best calibration (lowest Brier loss). Given its good performance and generalization on independent samples, the optimal generic model is the default model in the software. New models can be trained on user-provided data, such as demonstrated by the ATAC-seq model, also provided within the software. The output of the software shows detailed information to support decision-making by comparing results on user-provided data to ENCODE data (quality features and cross-validations). For example, the percentage of uniquely mapped reads can be compared to minimum, maximum, or median values of low- or high-quality ENCODE data, and the user can see additional statistics such as precision, recall, or F1 score achieved by the same model for different thresholds on output probabilities observed during cross-validations.

## Conclusions

We have statistically characterized common NGS quality features of a large set of data files and optimized their complex quality control using a machine learning approach. The derived statistical guidelines and predictive models represent a valuable resource for NGS specialists. Predictive models can be unbiased, accurate and to some extent widely applicable to unseen data types. Given enough labeled data for training, this approach could work for any type of NGS assay or species. Therefore, we strongly encourage researchers to share both high- and low-quality data with the community.

## Methods

### Dataset

To analyze the potential of machine learning applied to the quality assessment of NGS data files, we implemented a workflow as shown in (Fig. 1a). FastQ files and quality annotations were downloaded from the ENCODE data portal. The ENCODE status represents the result of a comprehensive manual inspection of the data by scientists from the ENCODE’s Data Coordination Center (DCC) according to the ENCODE guidelines with a main focus on the quality. FastQ files that are uploaded to ENCODE and assigned to an experiment are initially released. In ENCODE, the status of whole experimental datasets or single files is changed to revoked if they were deemed erroneous or significantly below standards after release. We used this status as an indication of the quality of a file and considered FastQ files to be low quality when revoked and high-quality when released.

We downloaded 1321 low-quality files plus the same number of high-quality files (total = 2642) to define balanced training and testing sets. For the selection of high-quality files, we prioritized files that are associated with an experiment that also contains revoked files. The remaining high-quality files were chosen randomly. From this full dataset, we defined data subsets as sets of files representing a combination of species and assay such as human ChIP-seq or mouse DNAse-seq (Fig. 1b). Because of their higher number of files and to facilitate comparisons between species, we used the following subsets for training machine learning models: human ChIP-seq (single-end and paired-end), mouse ChIP-seq (single-end), human and mouse DNAse-seq (paired-end), and human RNA-seq. ChIP-seq results are discussed in the article and plotted in figures only for single-end files (Additional file 1: Fig. S5 shows results for paired-end files).

### Deriving quality feature sets

We derived four different feature sets for the set of downloaded FastQ files as visualized in the sub-workflow in (Fig. 1a). The first feature set RAW was defined by eleven features from the summary statistics of the FastQC tool [5]. In the summary of a FastQC report, each statistic is flagged as Fail, Warning, or Pass. We use these flags as values for the features. The second feature set MAP contained the mapping statistics after applying Bowtie2 [6] to map the sequencing reads of human and mouse against the hg38 and mm10 genome assemblies, respectively. The mapping statistics describe the percentage of reads being unmapped, uniquely mapped, or multiply mapped and their overall mapping rate. Accordingly, there are four features for single-end and eight features for paired-end as these statistics are done for both the concordantly and discordantly mapped reads. The third feature set LOC is composed of nine features describing the distribution of reads mapped within the following types of genomic regions of interest: promoter, first intron, other introns, 5′UTR, first exon, other exons, 3′UTR, distal intergenic, and downstream proximal to the 3′UTR. The features were derived using the Bioconductor package ChIPseeker [30]. The fourth feature set TSS describes the distribution of reads near TSS (transcription start site) positions in the genome. The Bioconductor package ChIPpeakAnno [31] was used to compute the number of reads within the region 5 kb up- and downstream the TSS divided into ten bins, resulting in ten features for TSS identified by their central coordinate (e.g., TSS − 4500 denotes the genomic region with the following boundaries relative to TSSs: − 5 kb and − 4 kb). To reduce memory requirements during computation, the features for LOC and TSS were derived on one million mapped reads randomly sampled. For paired-end files, the RAW features were derived independently for each of the two files, while MAP, LOC, and TSS features were derived for the pair of files itself. In order to reduce redundancy in the dataset, we filtered out the RAW features for one member of each pair randomly. The largest files that were created within this data preprocessing are the FastQ and BAM files that sum up to a data set size of 5.6 TB and 2 TB, respectively.

The one-feature ROC-curves were derived by using the feature values as probabilities describing a non-machine learning probabilistic model. For example, the higher the uniquely mapped reads rate, the lower the probability of having a low-quality sample. A further example is the no mapping rate. In this case the higher these values, the higher the probability of having a low-quality sample. Hence, the features need to be treated differently. Therefore, we derived two ROC curves using first the default quality labels and second the inverted labels. Finally, the maximum area under the curve for a given feature was reported.

### Machine learning models

We conducted classification experiments on either all samples of the dataset (generic models) or on data subsets containing samples for a particular combination of species and assay (specialized models) as defined above. Based on the different species-assays and feature set combinations, machine learning algorithms were applied to train models that classify the quality class defined from the ENCODE status. A comprehensive grid search was applied to find the optimal algorithm and parameter setting for each subset. The algorithm set is listed within Fig. 4. In combination with parameter settings specific to each algorithm, a total of 19,417 different models were trained and evaluated within the grid search for each classification case. Furthermore, for each parameter setting, we applied three different feature selection methods prior to the classification. The feature selection selects the top k features based on chi-squared statistics, recursive feature elimination (RFE), and stability selection [32]. We analyzed four values for k: 100% (no feature selection), 75%, 50%, or 25%. Besides the scikit-learn grid search, we explored the potential of more complex and deeper neural networks [33] on the generic dataset (dropout regularization; up to 5 hidden layers); however, we could not find a configuration that outperforms the best Random Forest setting.

The predictive performance was evaluated by the area under receiver operating characteristic curve (auROC). Within the grid search and feature selection, a tenfold cross-validation was applied to evaluate the predictive performance. The entire grid search (including the preprocessing methods as well as the feature selection and nine of the classification algorithms) was implemented using the Python package scikit-learn [25]. The XGBoost algorithm, not supported by scikit-learn, was implemented only in the grid search using an external library [26]. The seqQscorer software used to derive further results in the article did not include XGBoost.

### Best performing model selection

Primarily, the best performing classification model was evaluated by the best auROC. For example, the optimal generic model trained on all data and features is a Random Forest model using the entropy as splitting criterion, the default maximum depth, the squared root function to define the maximum number of features, and 1000 estimators (trees). We additionally assessed all models by the Brier loss that represents how well the predicted probabilities are calibrated (the lower the better). However, we could not find models that drastically improved after calibration for most of the cases (Additional file 1: Fig. S8). The algorithms, parameters, and feature selection methods that achieved the highest auROC for all other generic and specialized cases in the grid search are shown in Additional file 5. Models used by the seqQscorer software are described in Additional files 3 and 4 (no XGBoost).

### Cross-species generalization

In order to test the generalization of classification models to data from unseen species, we performed classification experiments using training and testing sets containing data from different species, respectively. We used human and mouse ChIP-seq and DNase-seq data. Using a fivefold cross-validation, five training and testing sets were created for each species and the grid search was applied to the training sets. The best performing model for each training set was identified by the highest auROC achieved within a tenfold cross-validation on the training set. Finally, each of these models was evaluated twice, firstly on the corresponding testing set from the fivefold cross-validation that contains data from the same species and secondly on the corresponding testing set containing data from the differing species.

### Human diagnostic studies

For this analysis, we used 90 samples from 6 published datasets downloaded from GEO (Gene Expression Omnibus) the accessions are given below. The data was preprocessed based on the workflow that we also applied on the data from ENCODE as explained above and in Fig. 1a (extraction of quality feature sets). Quantification of gene expression was performed by the Salmon tool [27] on the human transcriptome GRCh38 as implemented in the Rasflow pipeline [34]. The counts were normalized using TPM [33]. Finally, for all the six GEO datasets, gene expression values were log2 transformed and standardized before applying the Principal Component Analysis. Dunn [35] indices were computed in R using the fpc package [36].

### External validation on datasets from Cistrome

We used 610 samples from 32 datasets available in GEO and referenced in the Cistrome [24] database to evaluate the quality predictions. The accessions, assay types, and other information about the datasets are given in Additional file 6. We computed low-quality probabilities of all samples using the optimal generic model. We also computed low-quality probabilities of the following samples using corresponding optimal specialized models: single-end or paired-end human ChIP-seq, single-end mouse ChIP-seq, paired-end human or mouse DNase-seq. For plotting, human and mouse paired-end DNase-seq models were used as specialized models for human and mouse paired-end ATAC-seq data, respectively, and single-end mouse ChIP-seq model was used on paired-end mouse ChIP-seq data.

From the Cistrome database, we used 5 numerical quality metrics derived by their own pipeline [37] that could be converted as bad quality flags if the value was below a recommended threshold (see Cistrome’s guidelines [24]). Similarly, good quality flags were derived for values above recommended thresholds. The 5 metrics are the raw sequence median quality score of FastQC (bad if < 25), the uniquely mapped reads of BWA (bad if < 60%) [7], the PBC score (bad if < 80%), the FRiP score (bad if < 1%) [21], and the union DHS (DNase I hypersensitive site) overlap (bad if < 70%) [38]. We compared our prediction probabilities in relation to the number of values indicating low quality, or bad quality flags, expecting a high number of these values to be associated with a high probability. We used this approach instead of a binary classification because the metrics that define the flags can represent actual quality very differently. All samples were annotated in Cistrome with at least 4 quality metrics.

### Custom human ATAC-Seq model

In order to create a labeled training set for a human ATAC-seq specialized model with the Cistrome data, we divided the samples in two groups as follows: if a sample had more than 3 good quality flags it was labeled as high-quality, low-quality otherwise. From 90 NGS samples, our arbitrary labeling resulted in a well-balanced training set with 49% low-quality samples. On this set, we trained a Random Forest model using the settings that achieved optimal results in our grid search on the generic case (all data types and features). This model was evaluated using a 10-fold cross-validation.

## Supplementary Information

**Additional file 1: Figure S1**. Statistical guidelines computed on the ENCODE files selection. **Figure S2.** Predictive performance of tuned machine learning models. **Figure S3.** Within-experiment benchmarks. **Figure S4.** Cross-species generalization. **Figure S5.** Paired-end human ChIP-seq data subset. **Figure S6.** Counts of broad peak targets in the ChIP-seq samples. **Figure S7.** Counts of sample names in the DNase-seq samples. **Figure S8.** Comparison of predictive and calibration performance. **Figure S9.** Peak-type specific one-feature predictions. **Figure S10.** Predictive performance of peak-type specific classification models. **Figure S11.** Cross validated predictions of the optimal generic model across most frequent ChIP-seq protein targets. **Figure S12.** External validations (RNA-Seq). **Figure S13.** Independent validation on Cistrome’s datasets. **Figure S14.** ENCODE guidelines and status.

**Additional file 2.** Table of grid search parameters. Parameters are relevant to a scikit-learn implementation.

**Additional file 3.** Table of tuned models used by seqQscorer. Dataset: species-subset-layout, generic: all data. Feature sets: RAW (raw data), MAP (genome mapping), LOC (genomic localization), TSS (transcription start sites profile). Feature Selection: method-percentage (percentage of retained features), chi-square (chi2), recursive feature elimination (RFE). Algorithm Parameters: relevant to a scikit-learn implementation.

**Additional file 4.** Table of optimal models used by seqQscorer. Optimal models are the best models for each data subset (Dataset column). Dataset: species-subset-layout, generic: all data. Feature sets: RAW (raw data), MAP (genome mapping), LOC (genomic localization), TSS (transcription start sites profile). Feature Selection: method-percentage (percentage of retained features), chi-square (chi2), recursive feature elimination (RFE). Algorithm Parameters: relevant to a scikit-learn implementation.

**Additional file 5.** Table of models tuned using the grid search. Dataset: species-subset-layout, generic: all data. Feature sets: RAW (raw data), MAP (genome mapping), LOC (genomic localization), TSS (transcription start sites profile). Feature Selection: method-percentage (percentage of retained features), chi-square (chi2), recursive feature elimination (RFE). Algorithm Parameters: relevant to a scikit-learn implementation.

**Additional file 6.** Table of Cistrome datasets. The table details all Cistrome datasets used for independent validation. Dataset GSE60731 is listed 2 times to represent either mouse DNase-seq samples (GSE60731) or ChIP-seq samples (GSE60731_2). GEO_Series: identifier from the GEO database.

**Additional file 7.** Table of ENCODE datasets. The table details all ENCODE datasets used for training the model). Quality label is 0 for released files or 1 for revoked files.

**Additional file 8.** Review history.

## Data Availability

The latest version of the seqQscorer software is freely available online on GitHub [29] under an open-source MIT license. The GitHub documentation describes the optional usage of a Docker image [39] which is freely available online on Docker hub [40]. The version used to analyze the data in this article is indexed and stored on Zenodo [41]. The datasets analyzed during the current study are available in the ENCODE database [42], Cistrome [43], and GEO repositories [44]. Accessions of ENCODE datasets used for training the models are detailed in Additional file 7. GEO accessions of Cistrome datasets used for independent validation are detailed in Additional file 6. GEO accessions of human diagnostic RNA-seq studies: Alzheimer’s disease (GSE125583), Crohn’s disease (GSE66207), diabetes (GSE92724), sporadic amyotrophic lateral sclerosis (GSE76220), liver cancer (GSE25599), and thyroid papillary carcinoma (GSE63511).
