# Supplementary material for: seqQscorer: automated quality control of next-generation sequencing data using machine learning
Source: Genome Biol. 2021 Mar 5;22:75. doi: 10.1186/s13059-021-02294-2 (PMC7934511; doi:10.1186/s13059-021-02294-2)

Supplementary Figures

Table of Contents

[Fig. S1 - Statistical guidelines computed on the ENCODE files selection 2](#_Toc63760760)

[Fig. S2 - Predictive performance of tuned machine learning models 17](#_Toc63760761)

[Fig. S3 - Within-experiment benchmarks 22](#_Toc63760762)

[Fig. S4 - Cross-species generalization 24](#_Toc63760763)

[Fig. S5 - Paired-end human ChIP-seq data subset 26](#_Toc63760764)

[Fig. S6 - Counts of broad peak targets in the ChIP-seq samples 27](#_Toc63760765)

[Fig. S7 - Counts of sample names in the DNase-seq samples 28](#_Toc63760766)

[Fig. S8 - Comparison of predictive and calibration performance 29](#_Toc63760767)

[Fig. S9 - Peak-type specific one-feature predictions 31](#_Toc63760768)

[Fig. S10 - Predictive performance of peak-type specific classification models 32](#_Toc63760769)

[Fig. S11 - Cross validated predictions of the optimal generic model across most frequent ChIP-seq protein targets 33](#_Toc63760770)

[Fig. S12 - External validations (RNA-Seq) 35](#_Toc63760771)

[Fig. S13 - Independent validation on Cistrome’s datasets 38](#_Toc63760772)

[Fig. S14 - ENCODE guidelines and status 39](#_Toc63760773)

# Fig. S1 - Statistical guidelines computed on the ENCODE files selection

For each data subset, bar plots or boxplots show the distributions of (**A**) the raw data features derived by the FastQC tool, (**B**) the genome mapping features (MAP) derived by Bowtie2, (**C)** the genomic localization features (LOC) derived by the ChIPSeeker R library, (**D)** the transcription start sites profile features (TSS) derived by the ChIPpeakAnno R library.

**A**


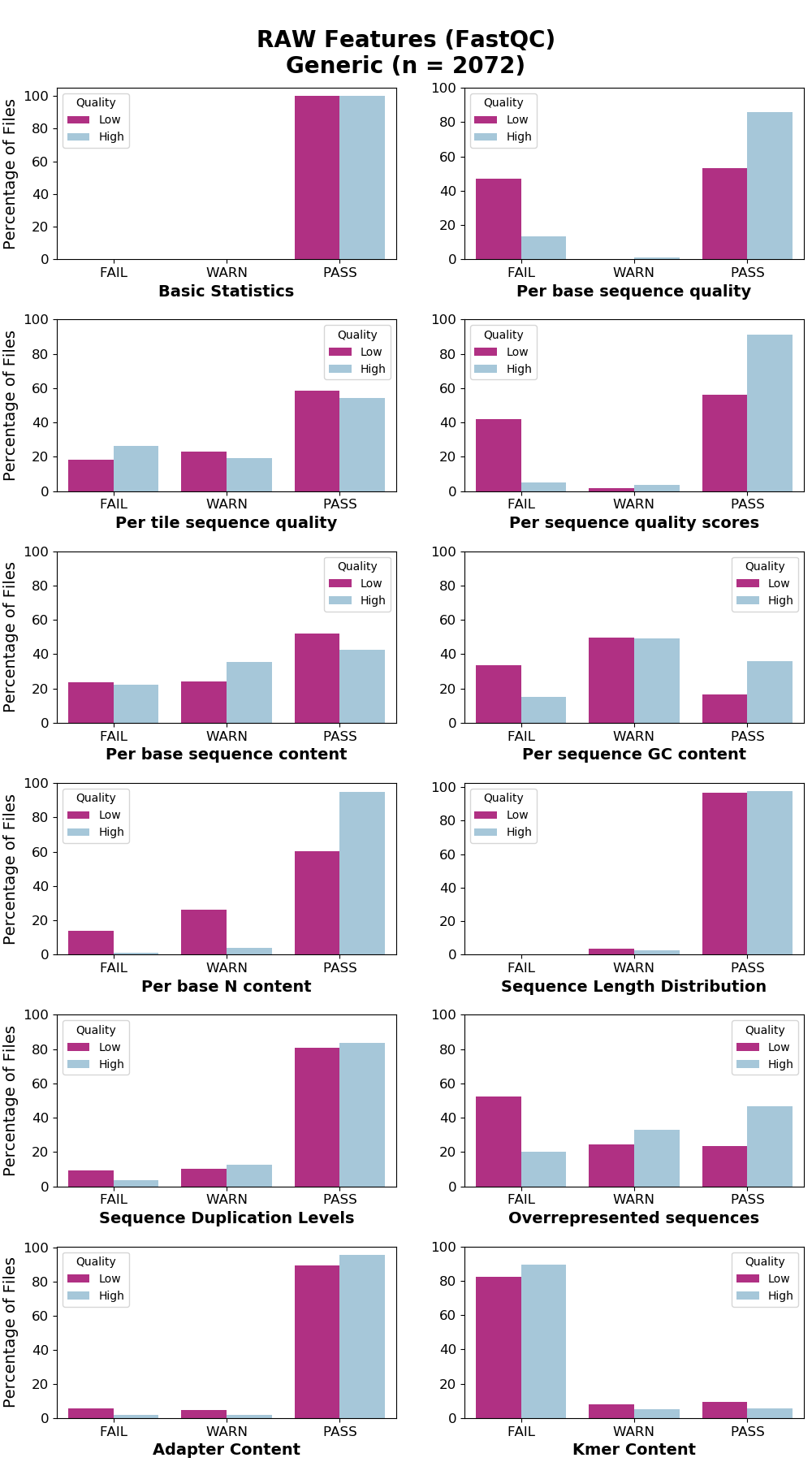


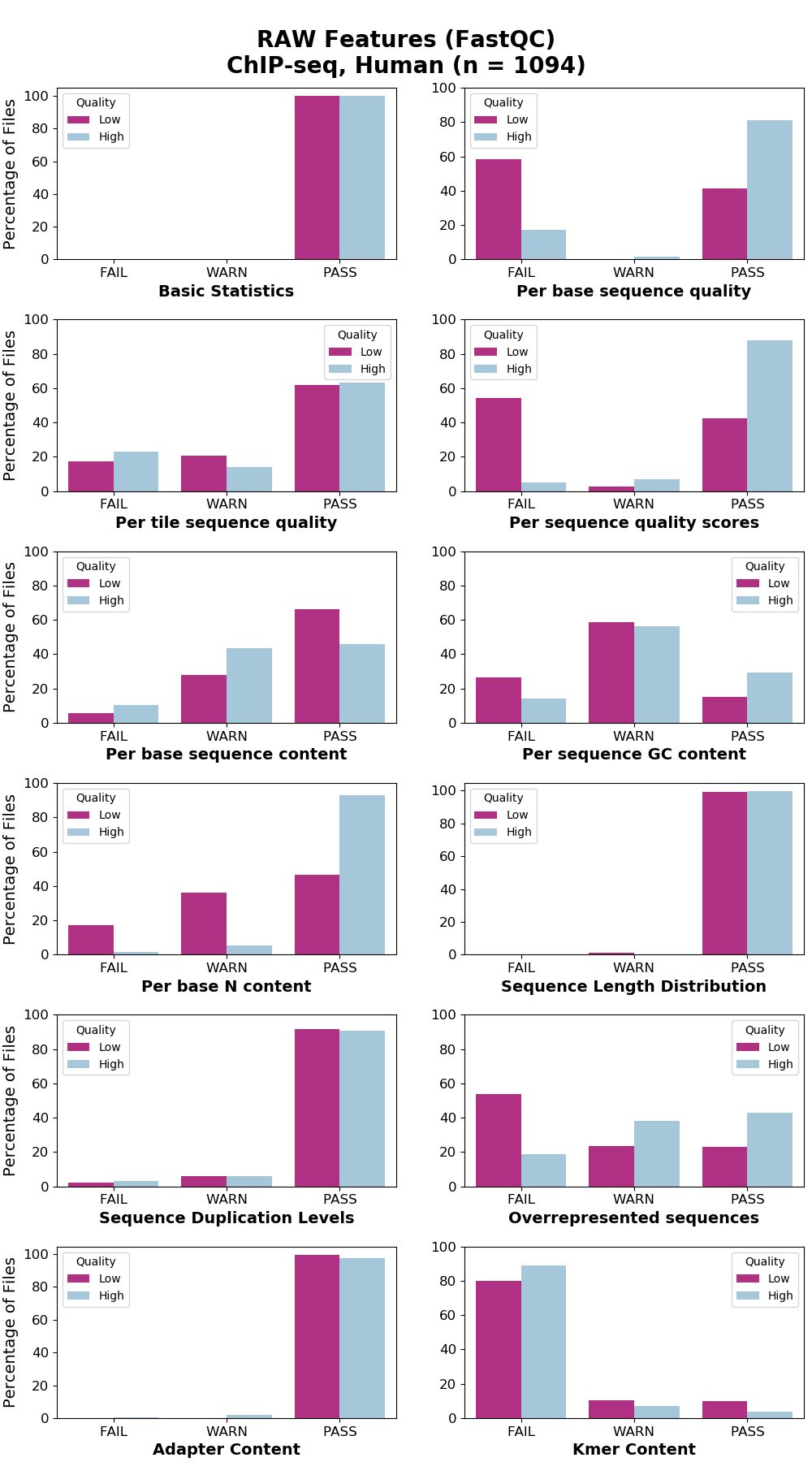

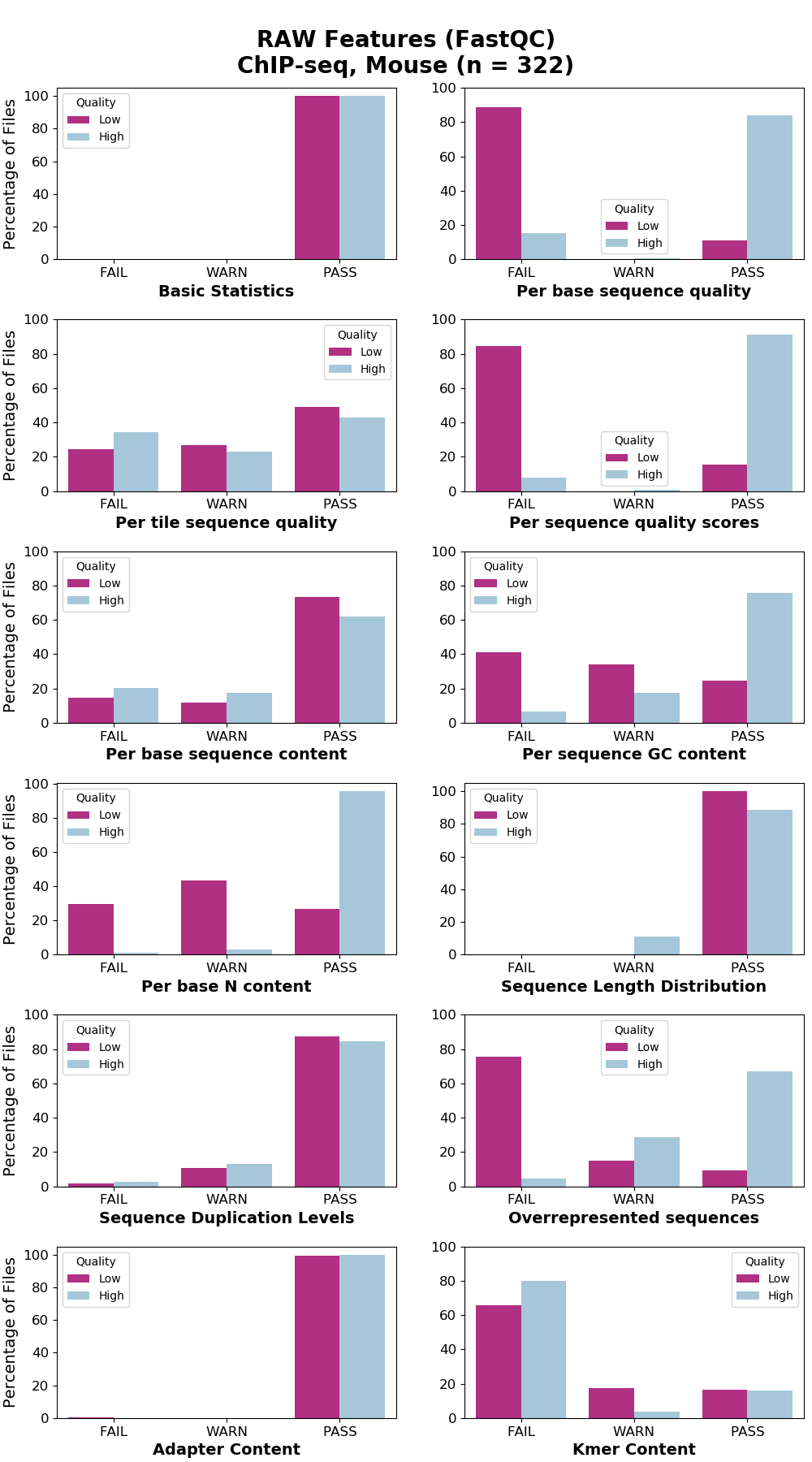


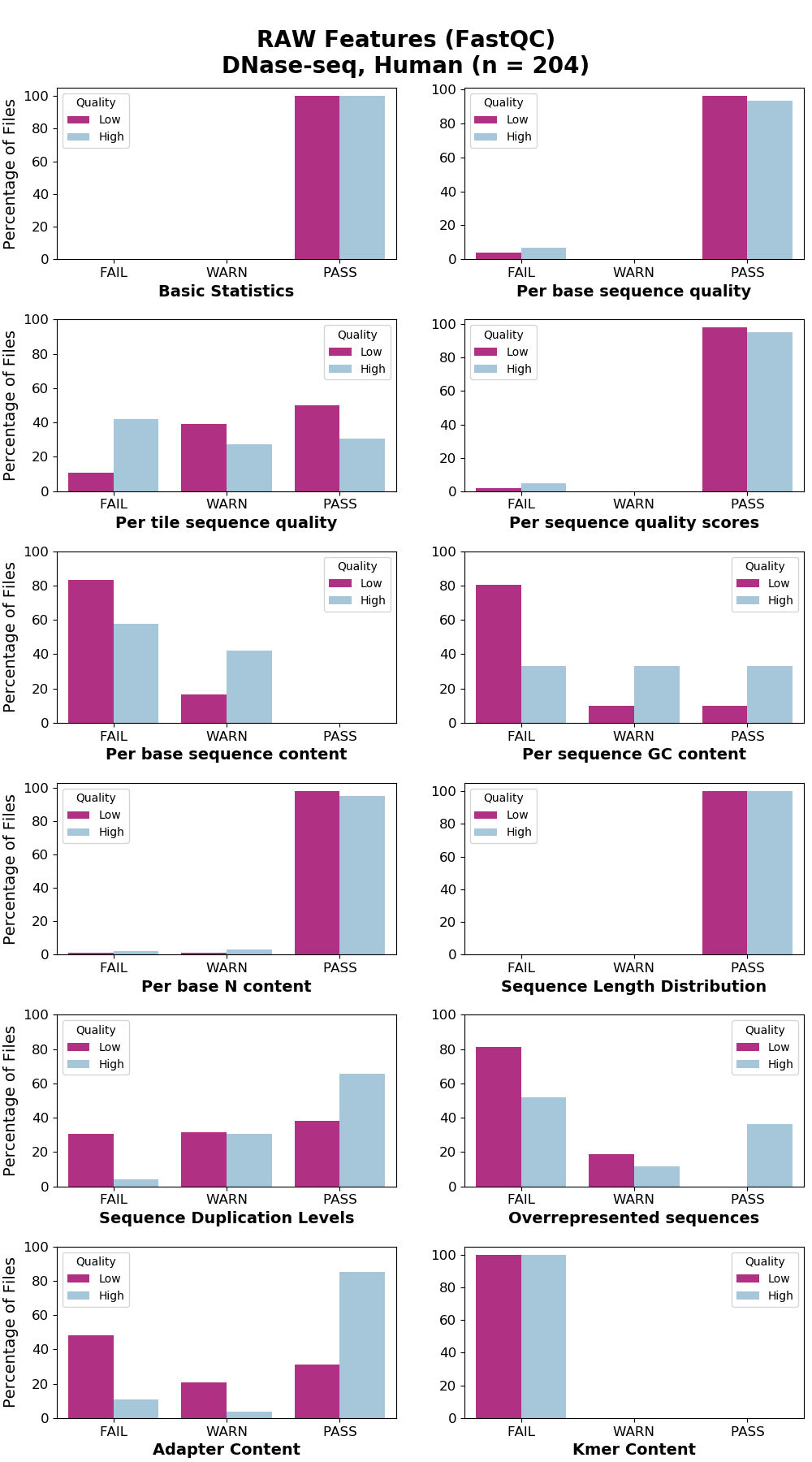


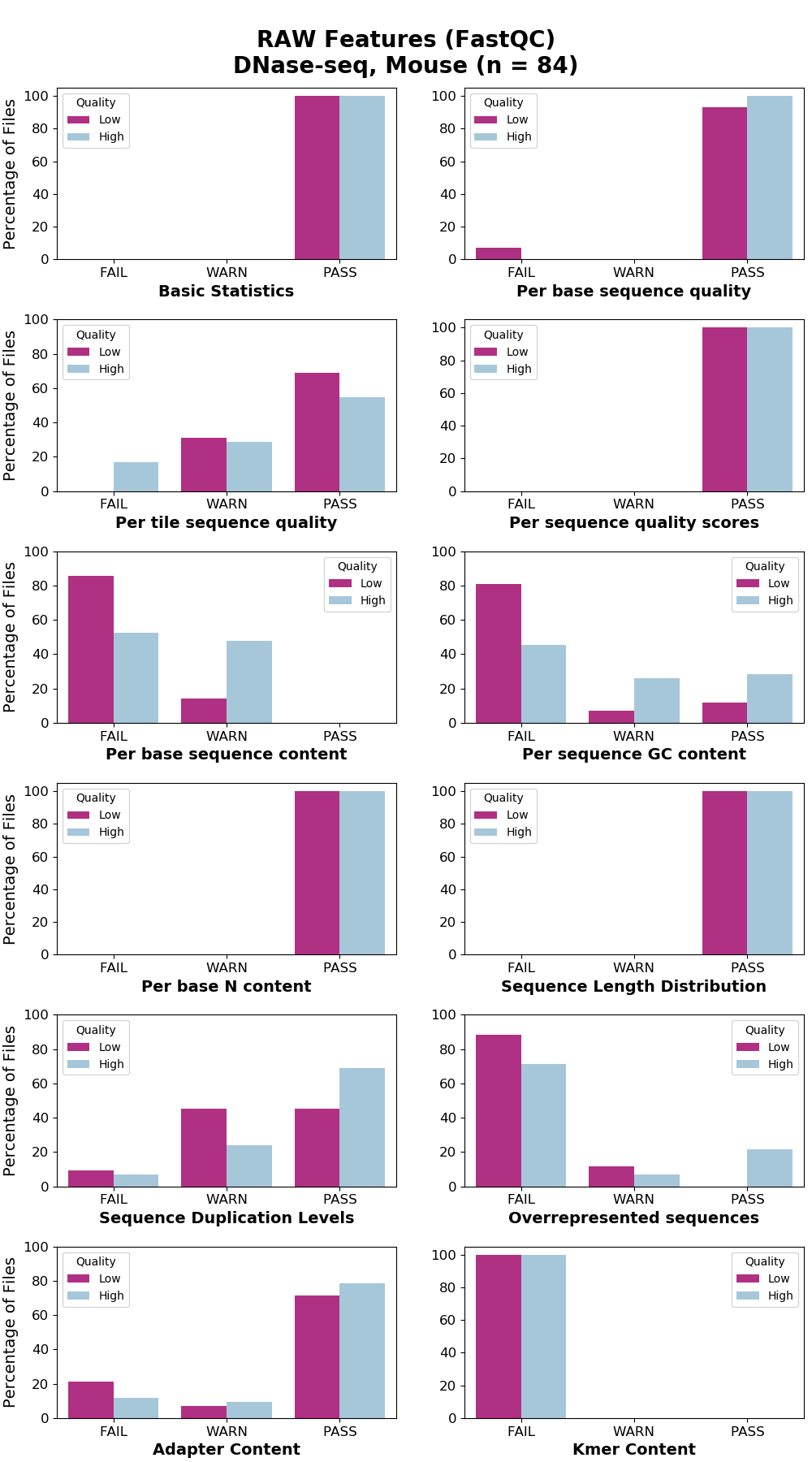

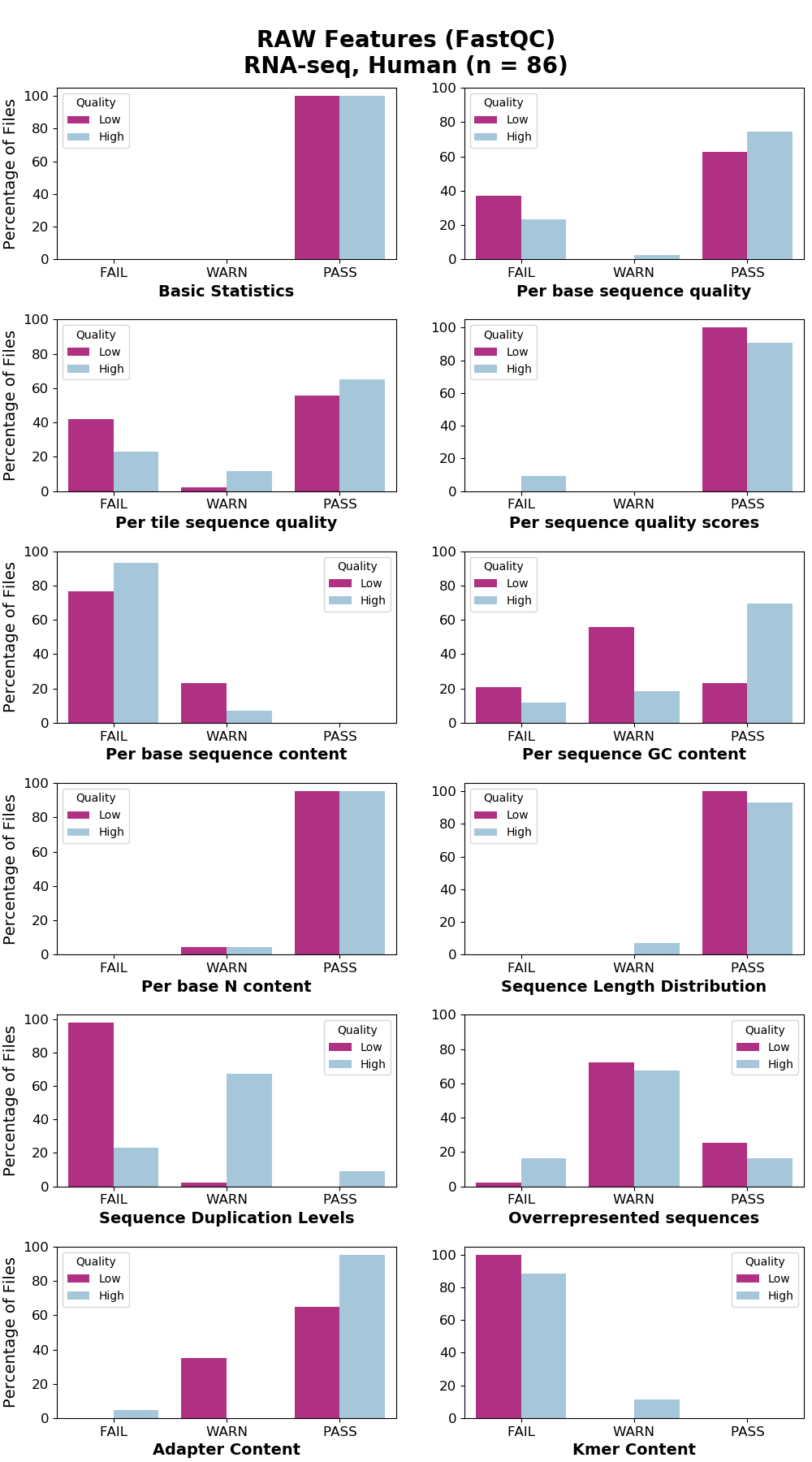


**B**


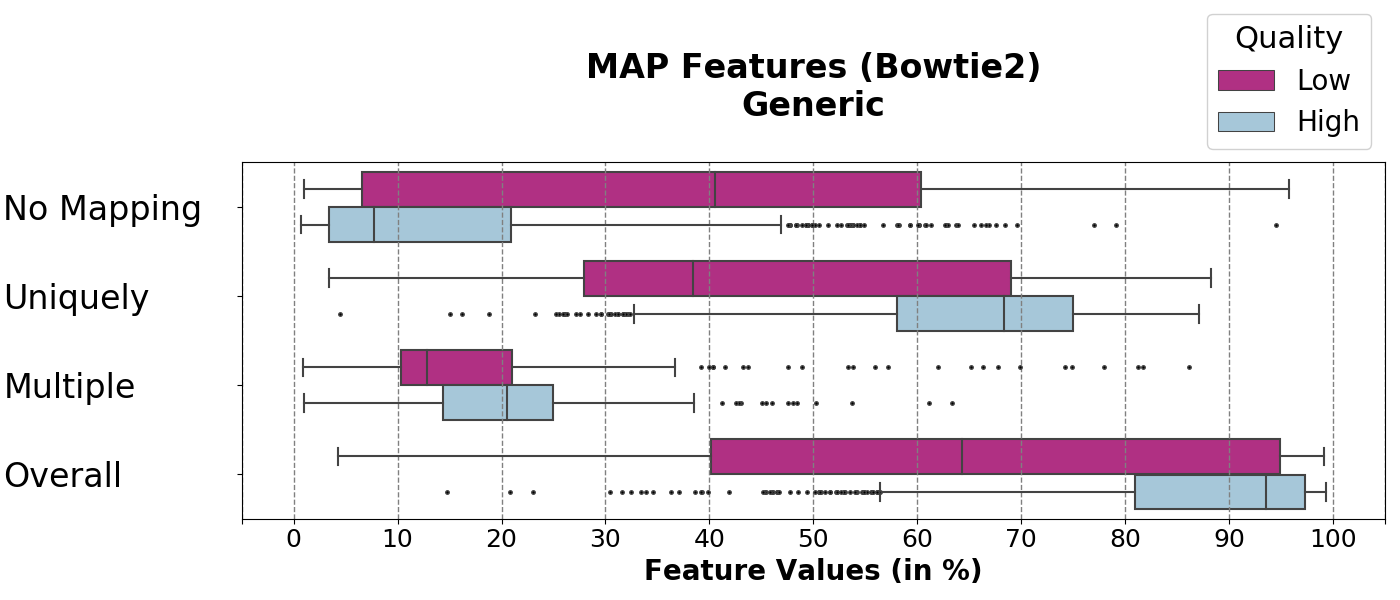


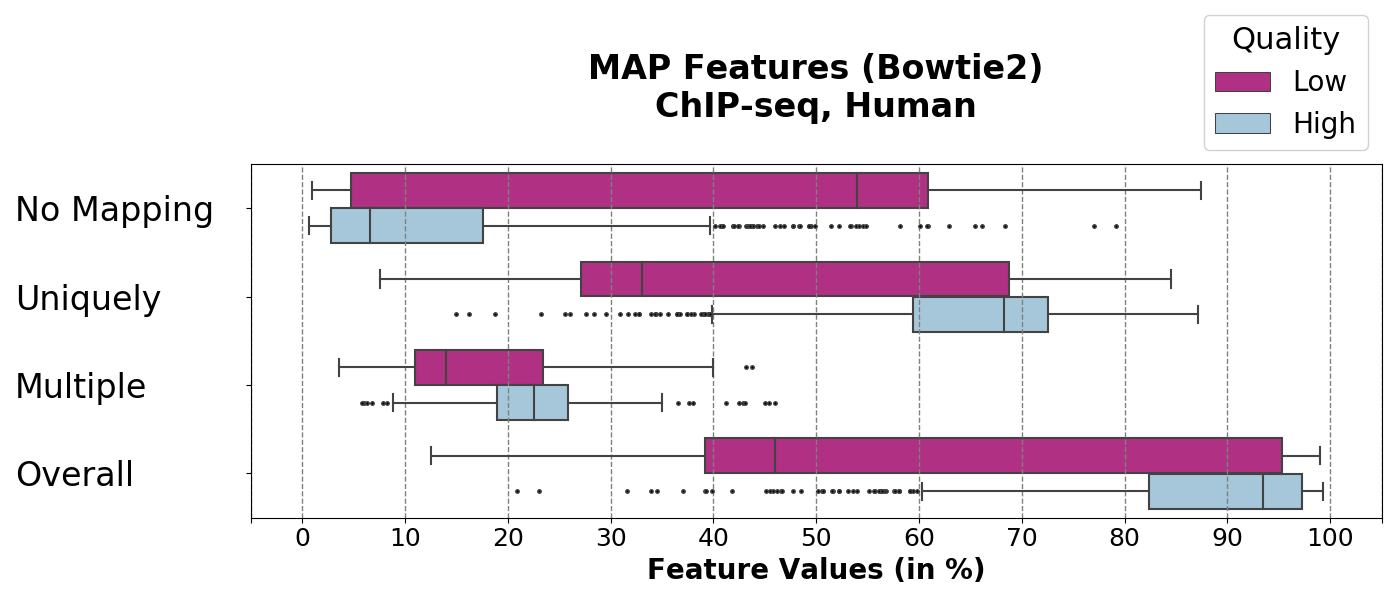


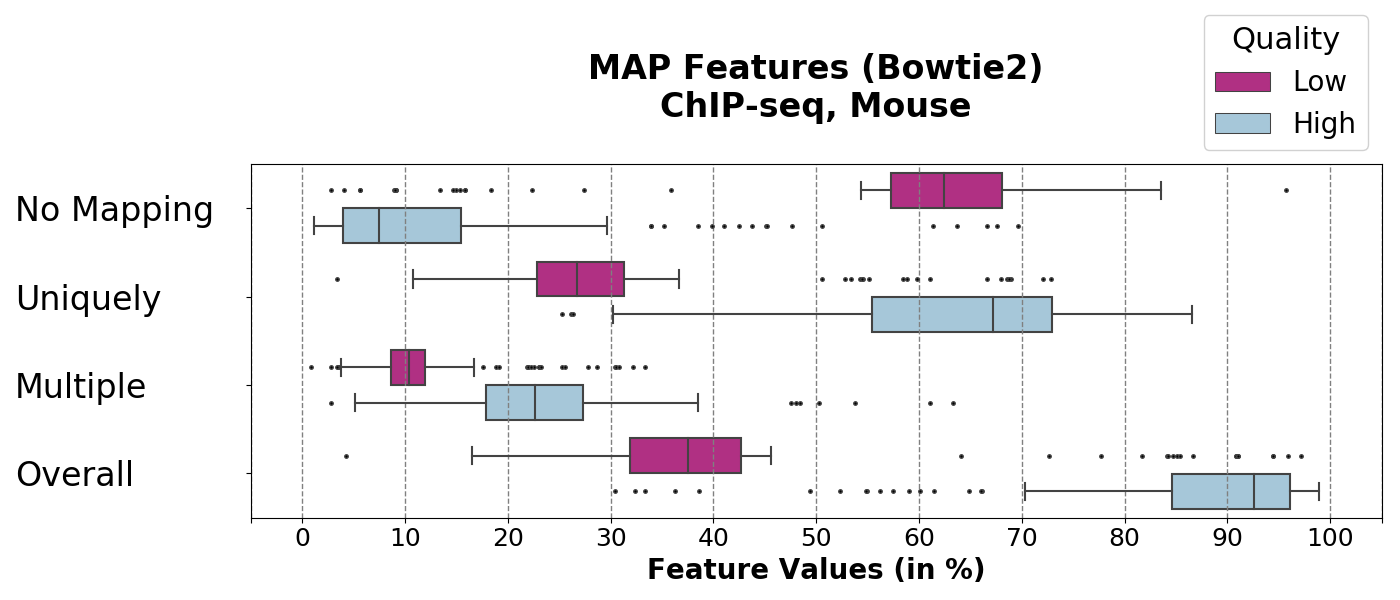


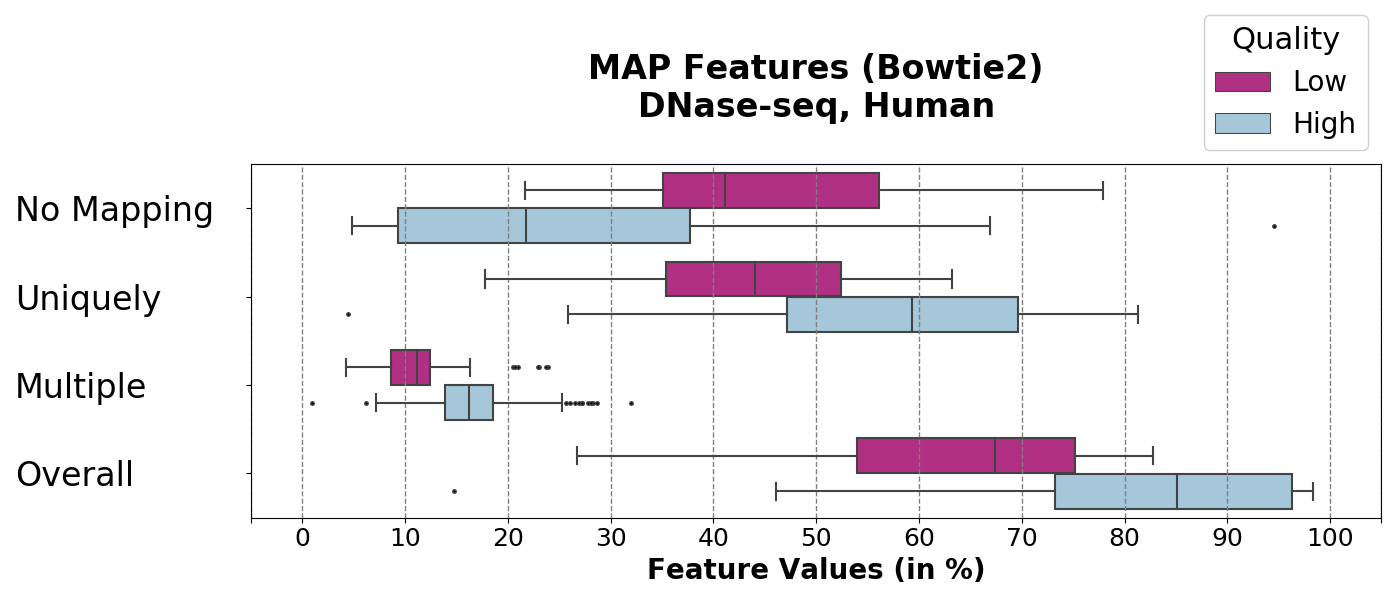


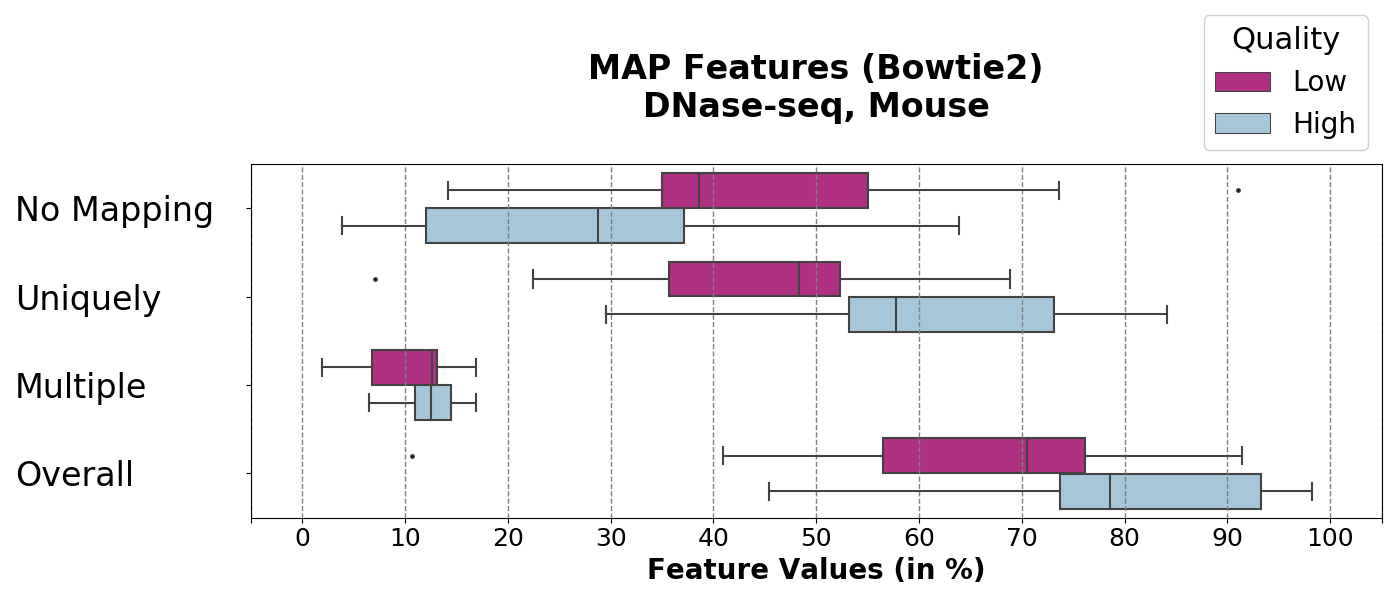


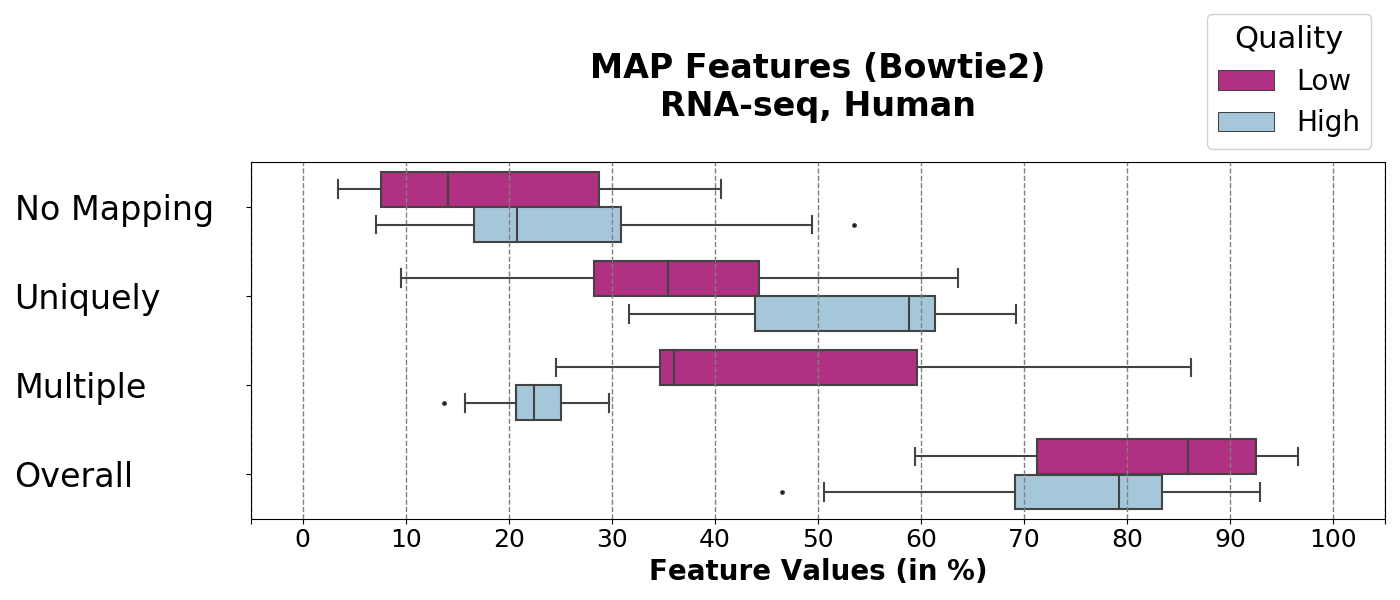


**C**


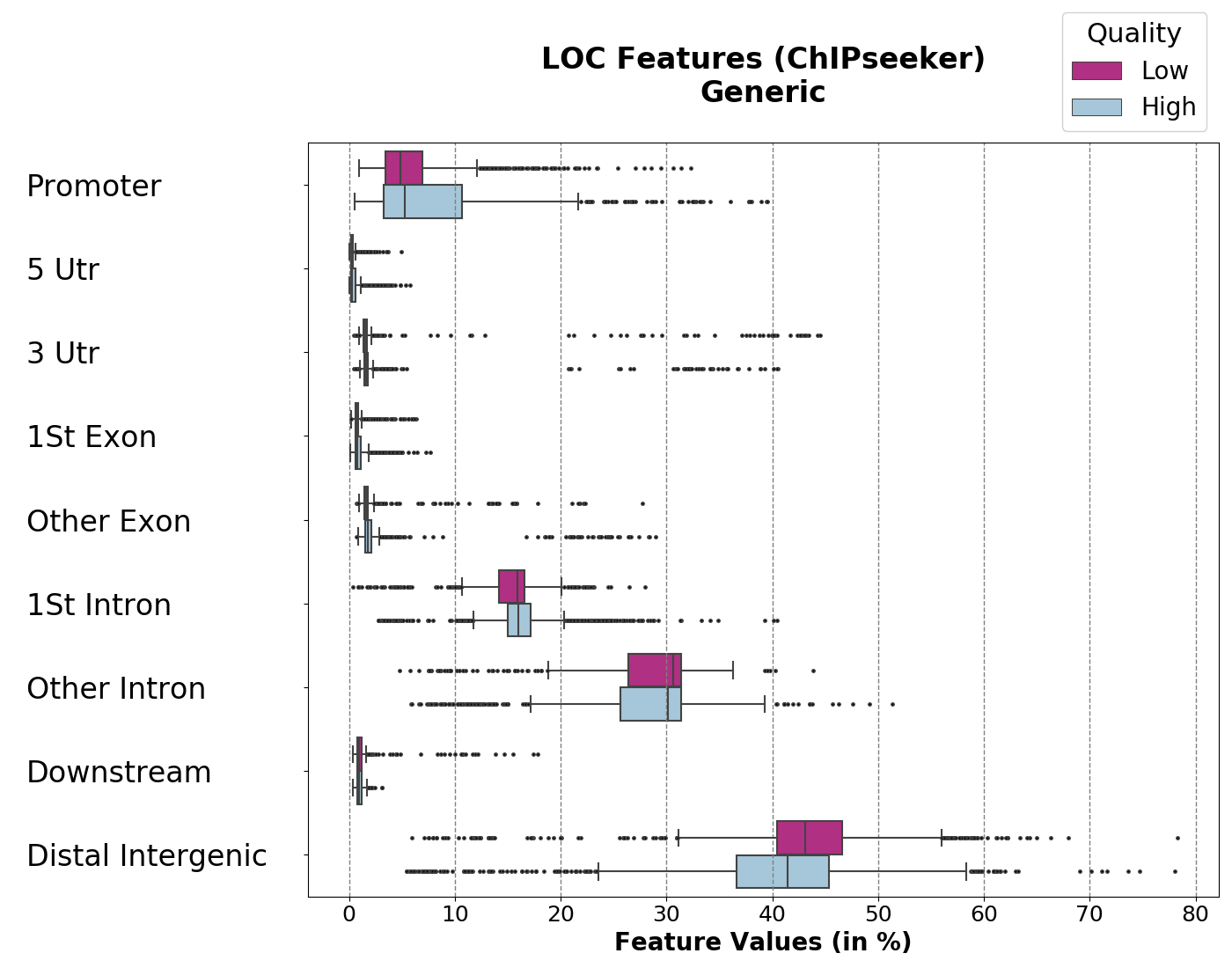


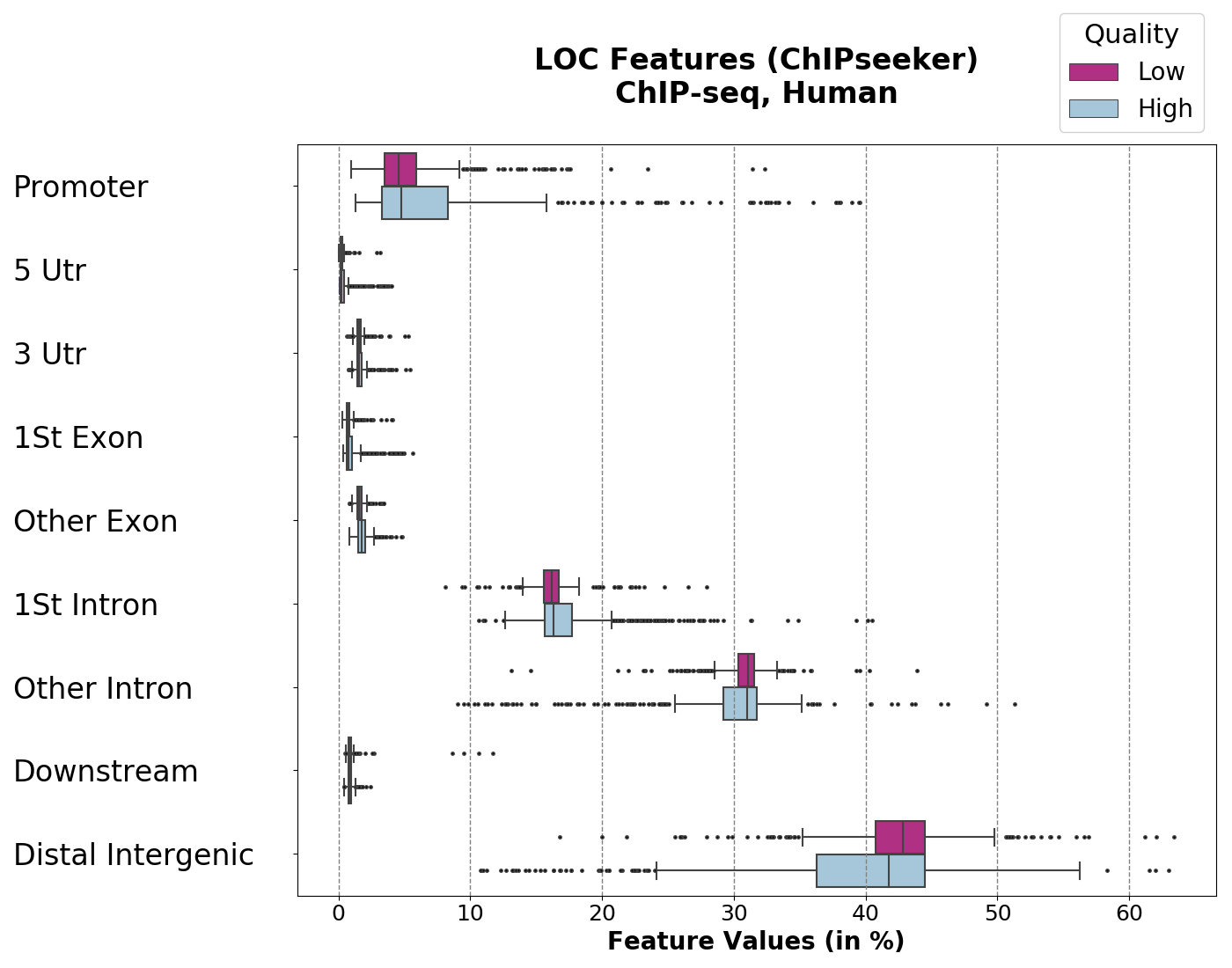


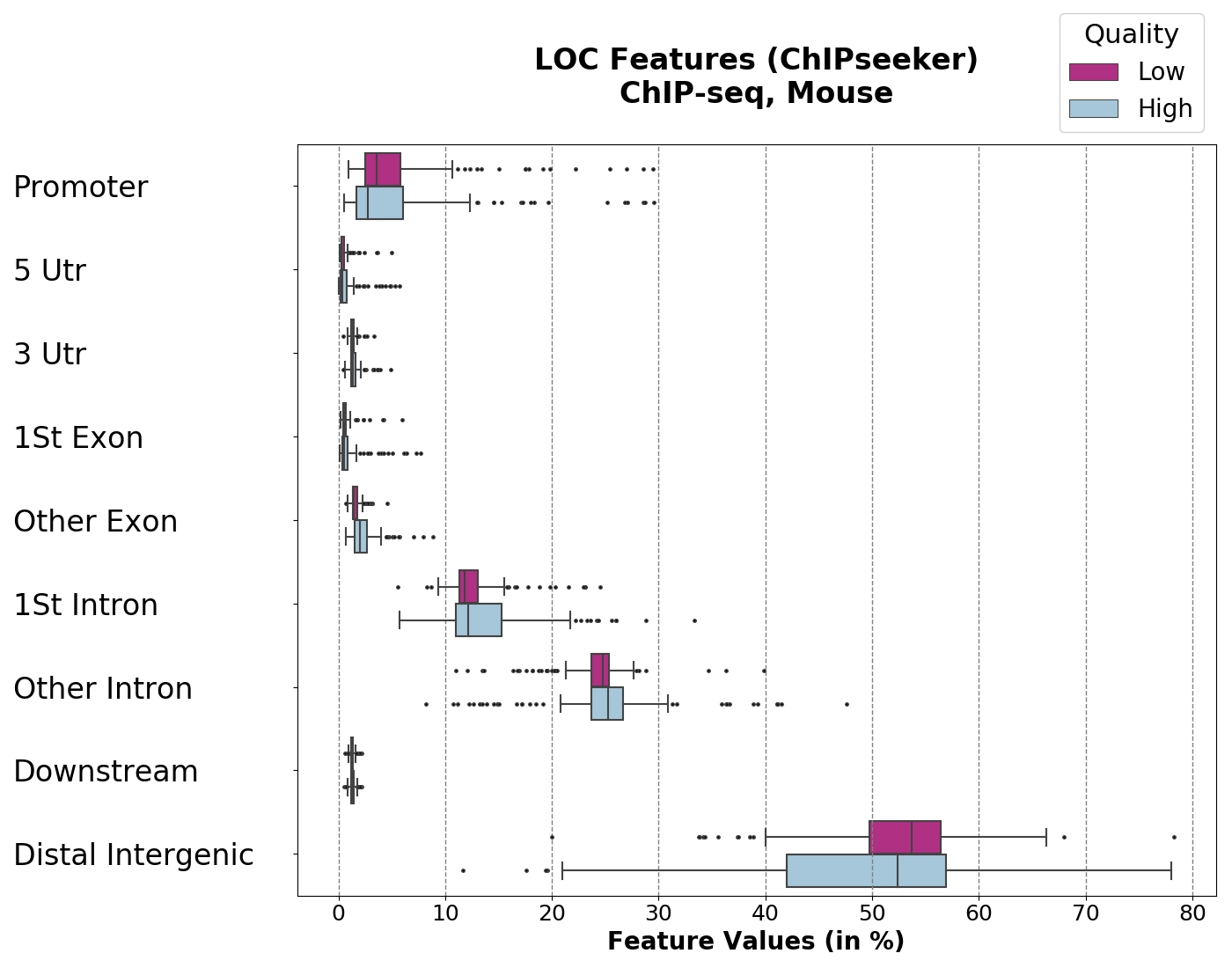


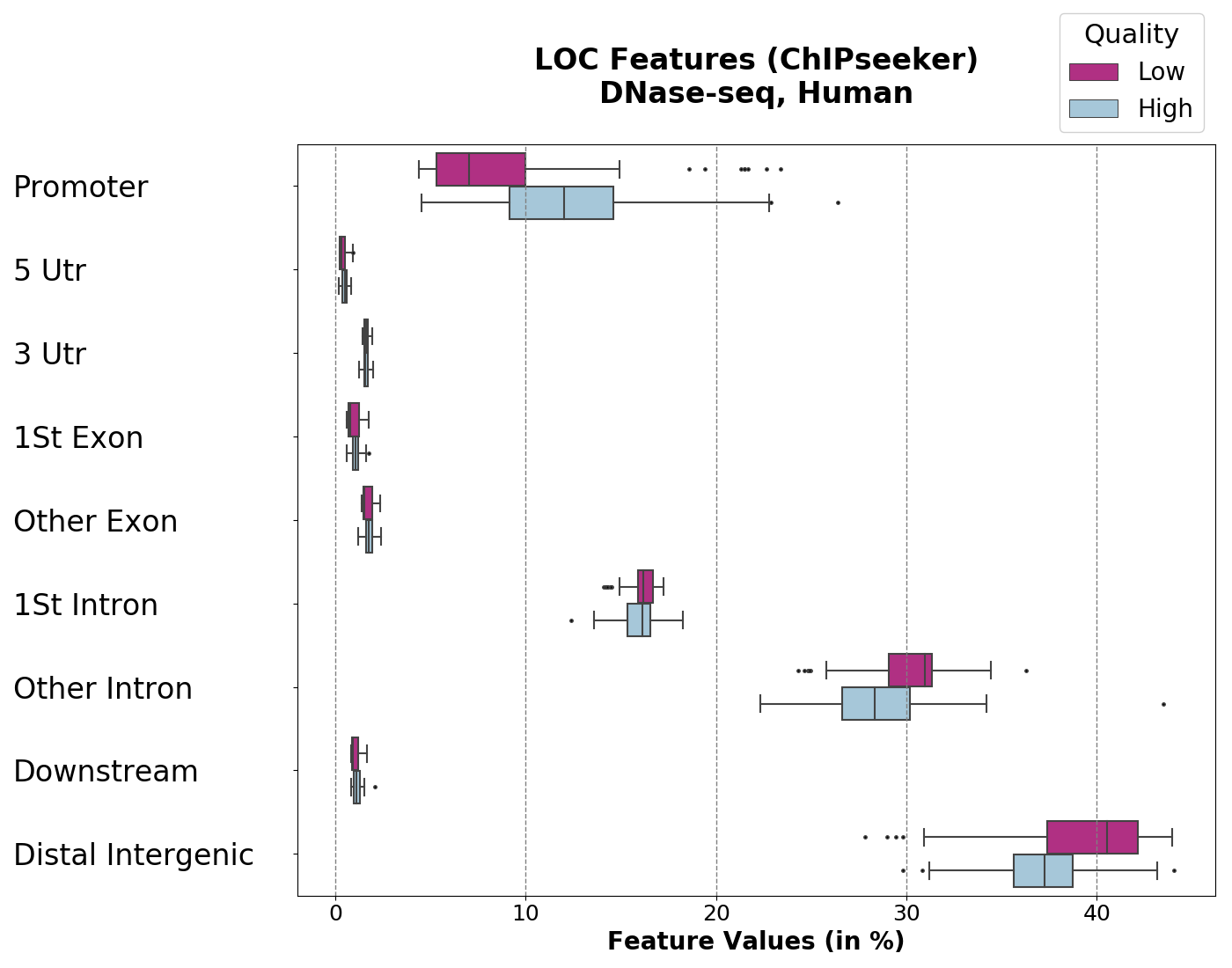


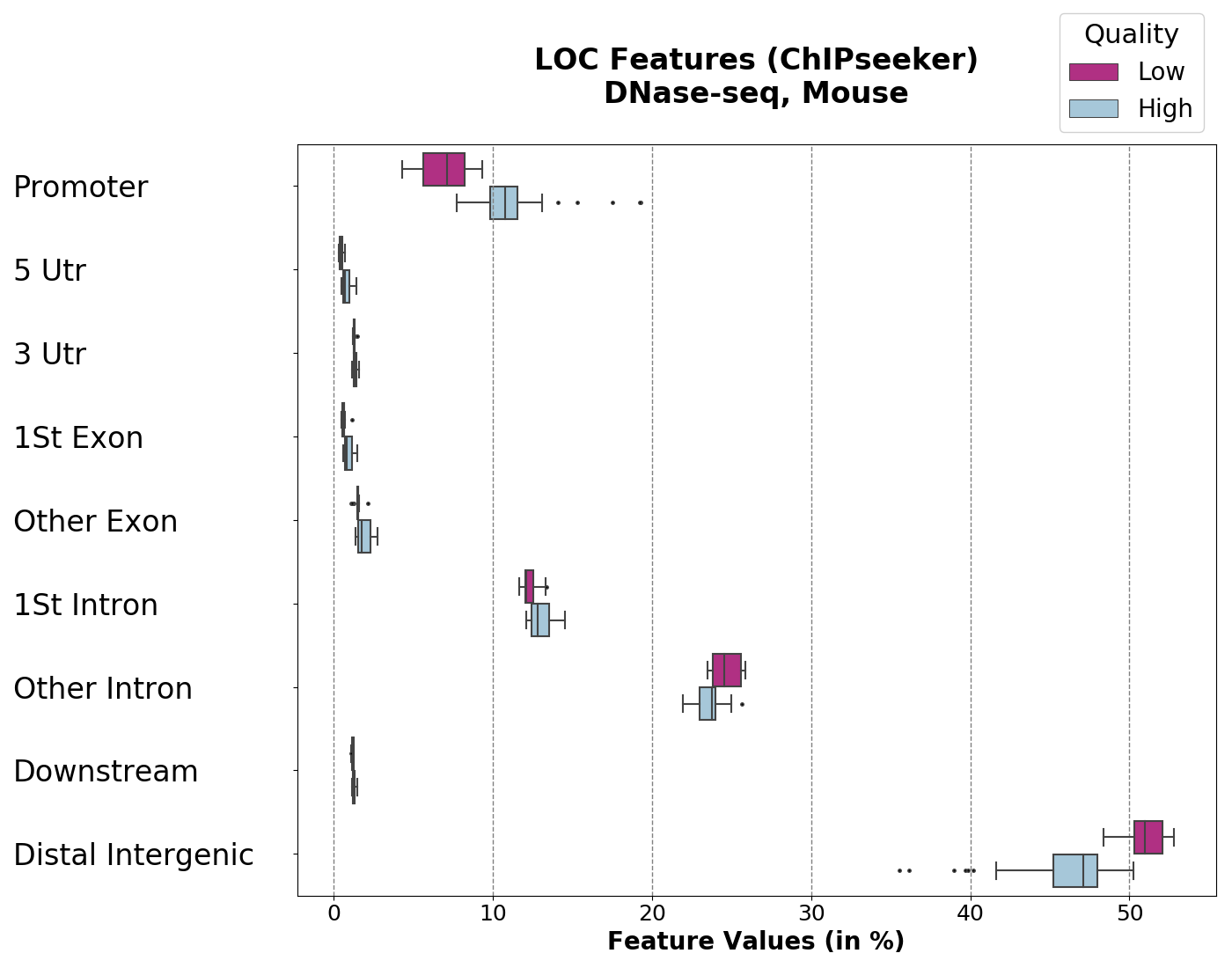


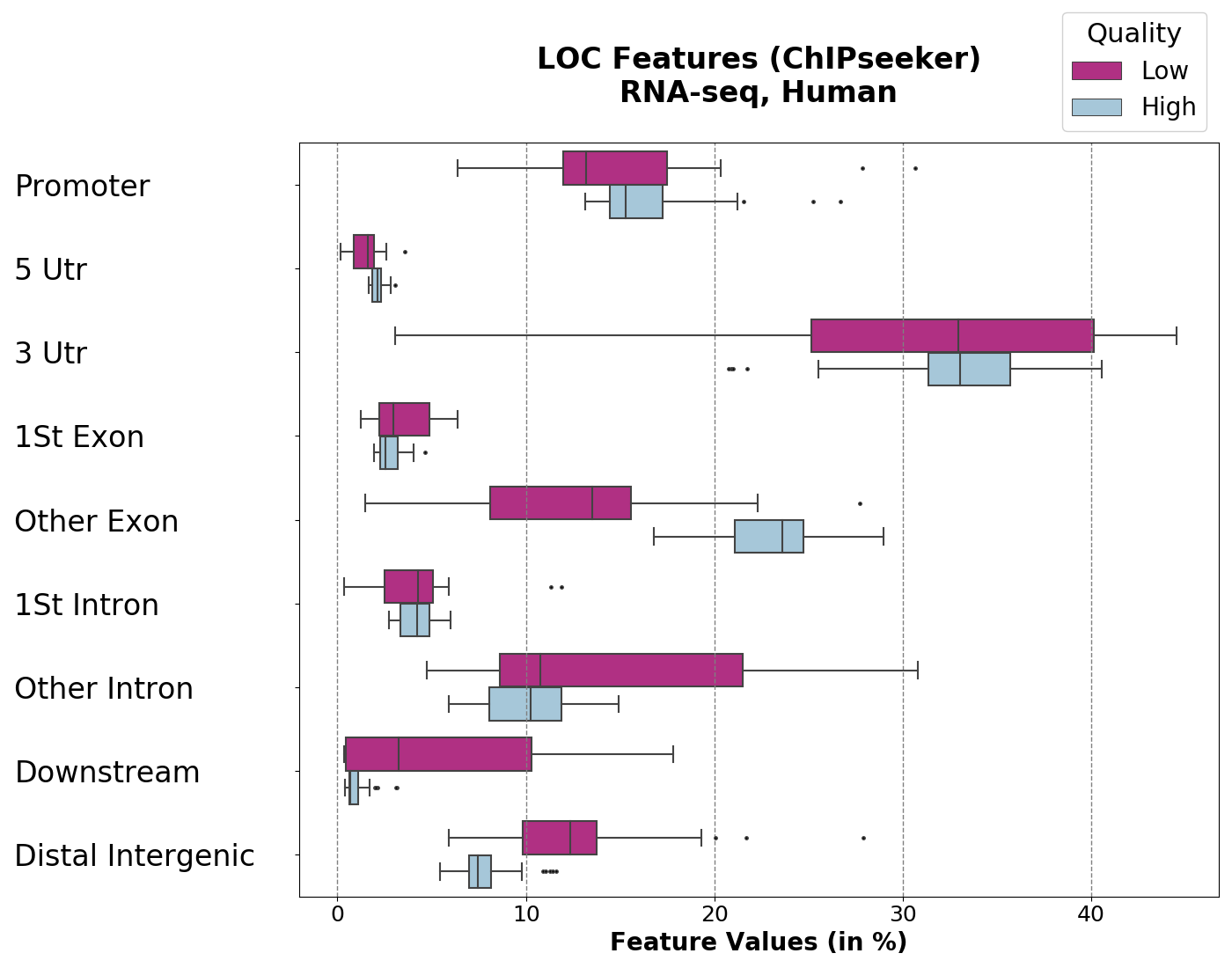


**D**


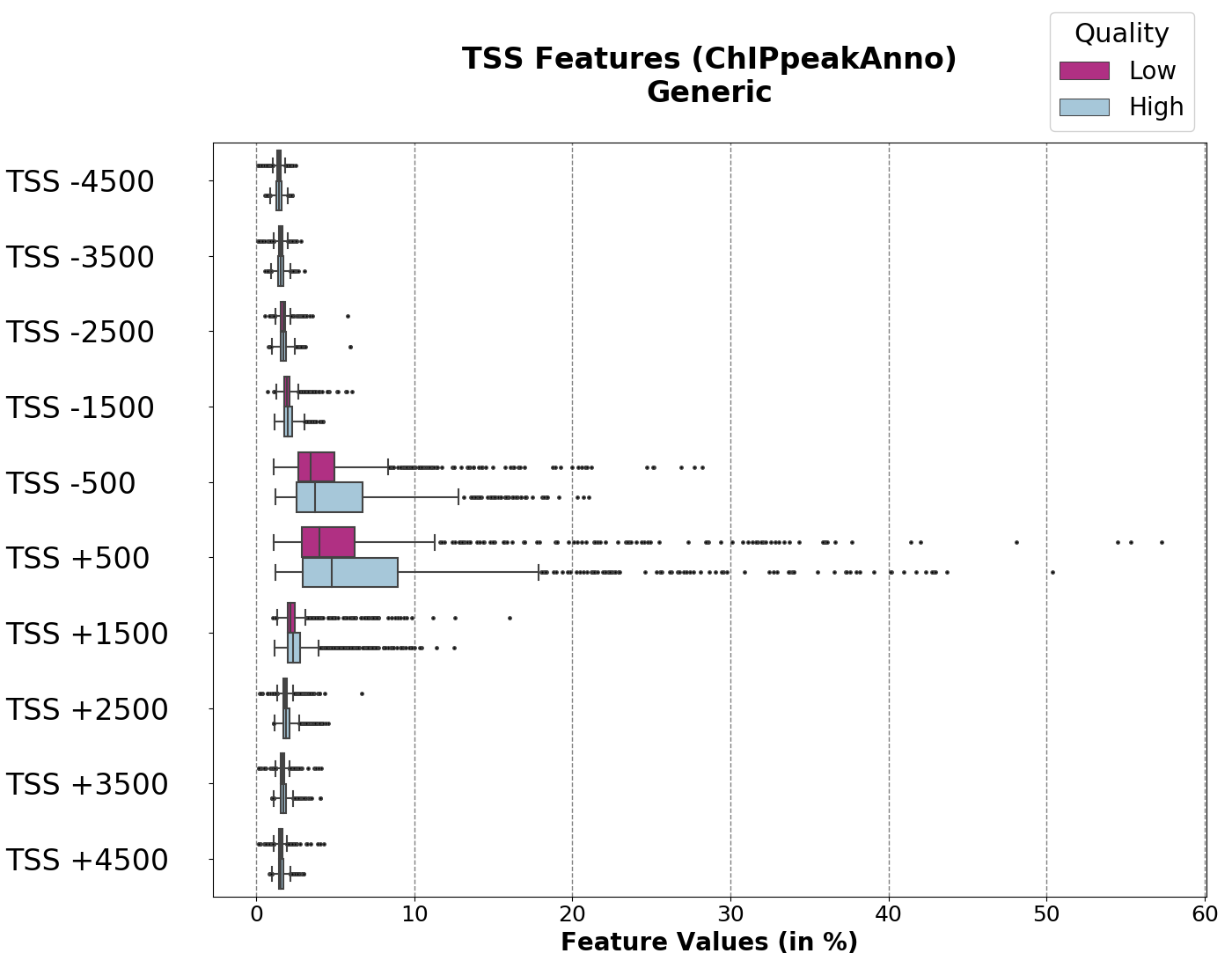


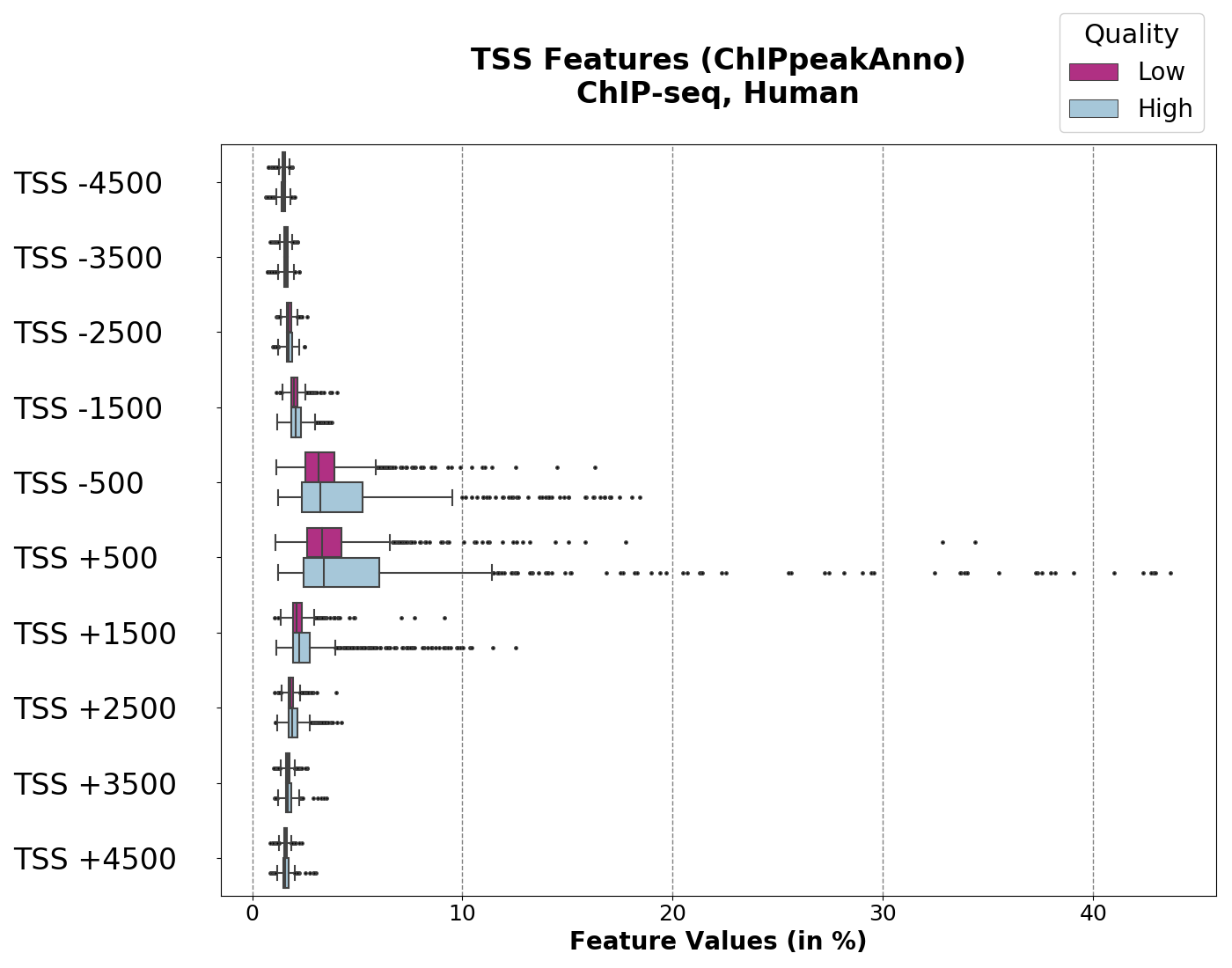


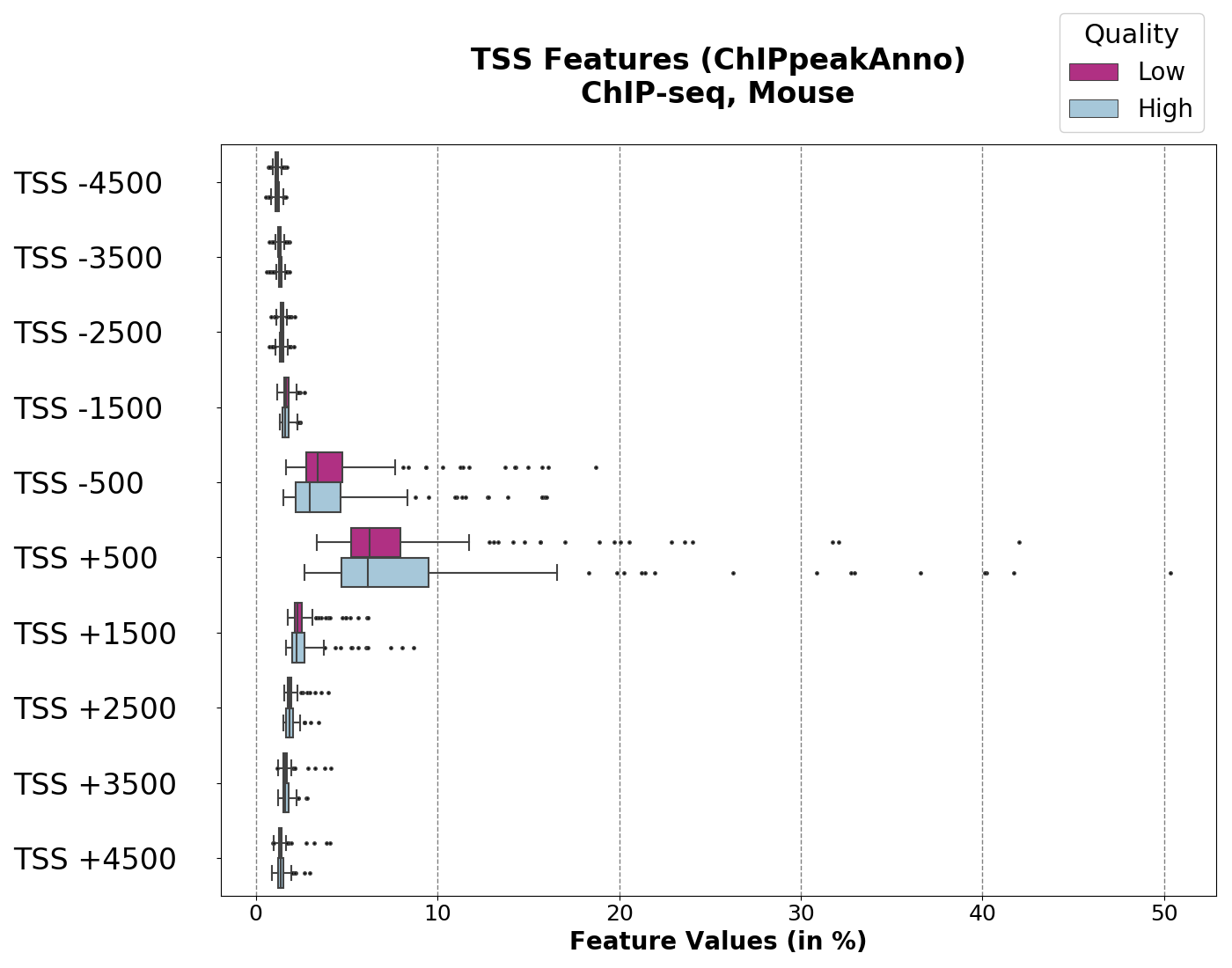


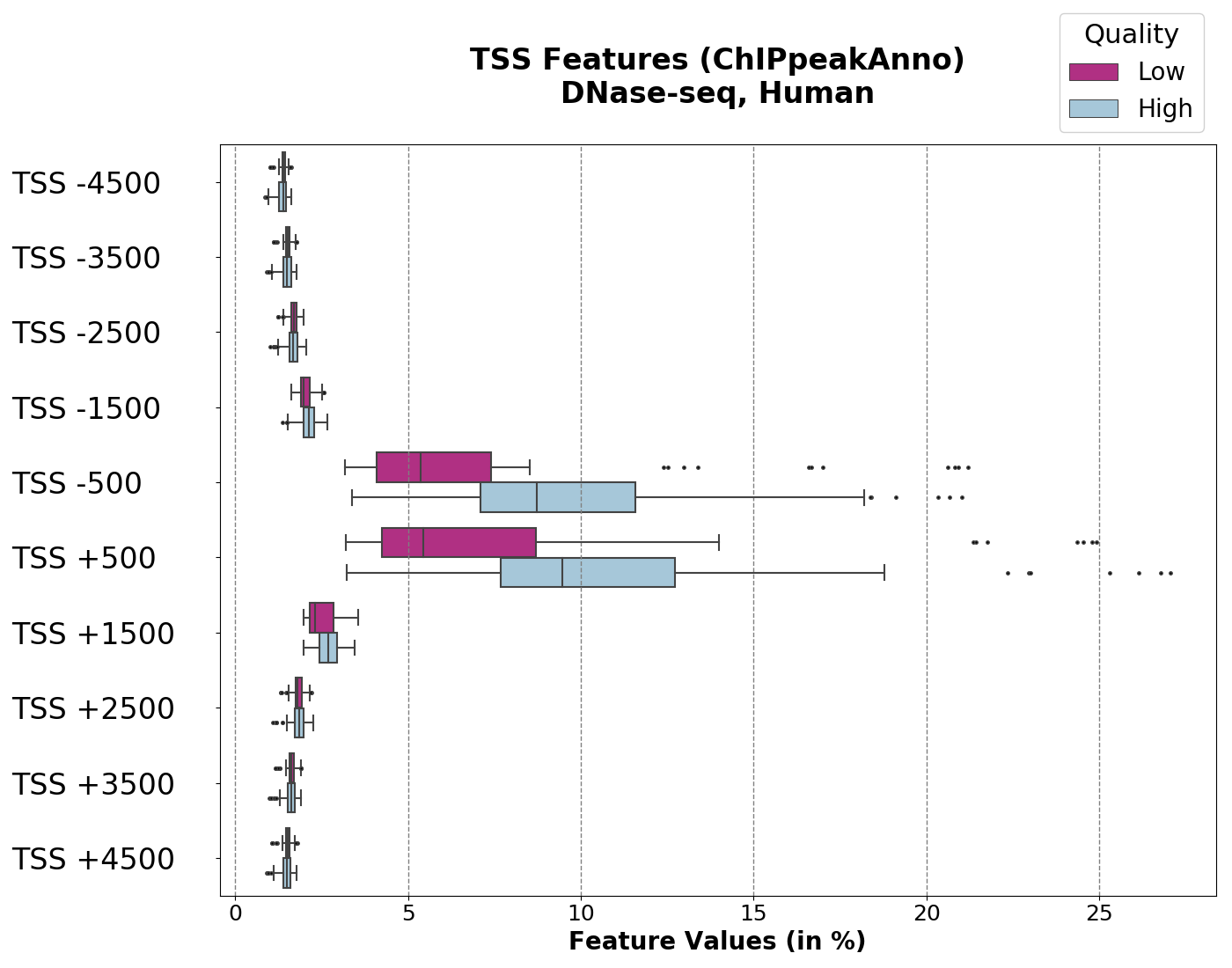


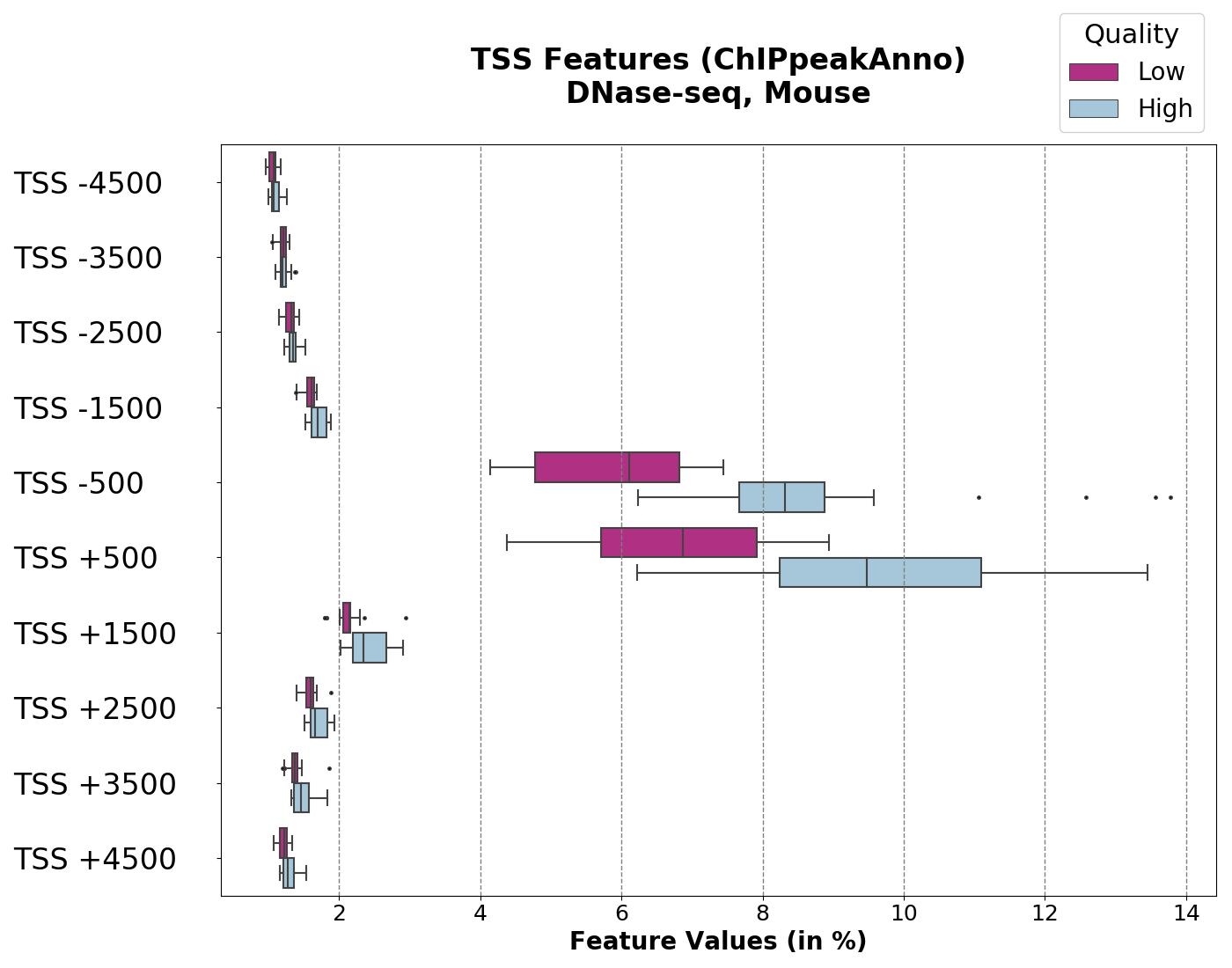


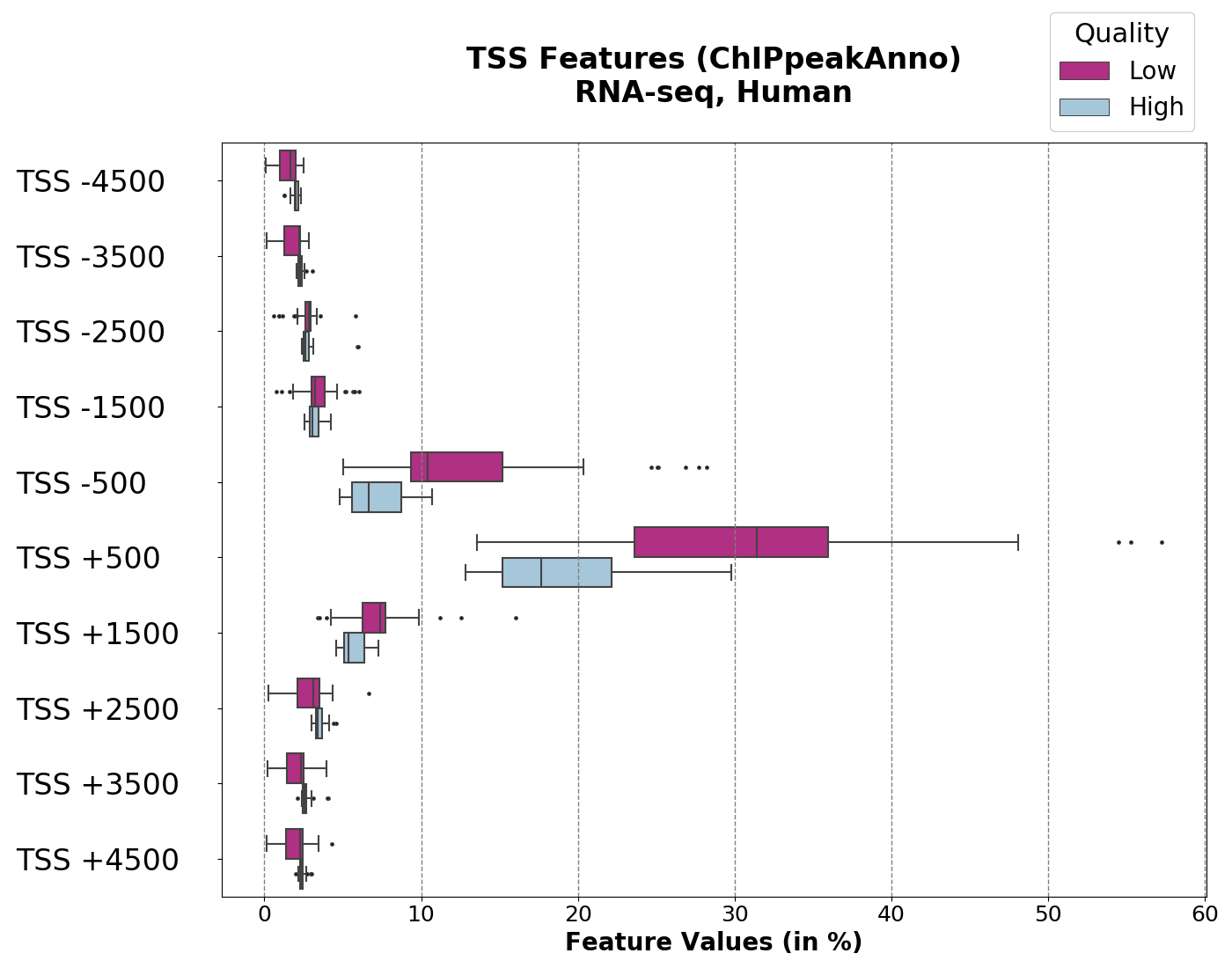


# Fig. S2 - Predictive performance of tuned machine learning models

Models tuned by a grid search were defined for each data subset (y-axis) and all possible combination of feature sets. Several performance measures were used: (**A**) area under ROC-curve (auROC), (**B**) area under precision-recall curve (auPRC), (**C**) accuracy (ACC), and (**D**) F1 measure. Feature sets: RAW (raw data), MAP (genome mapping), LOC (genomic localization), TSS (transcription start sites profile).


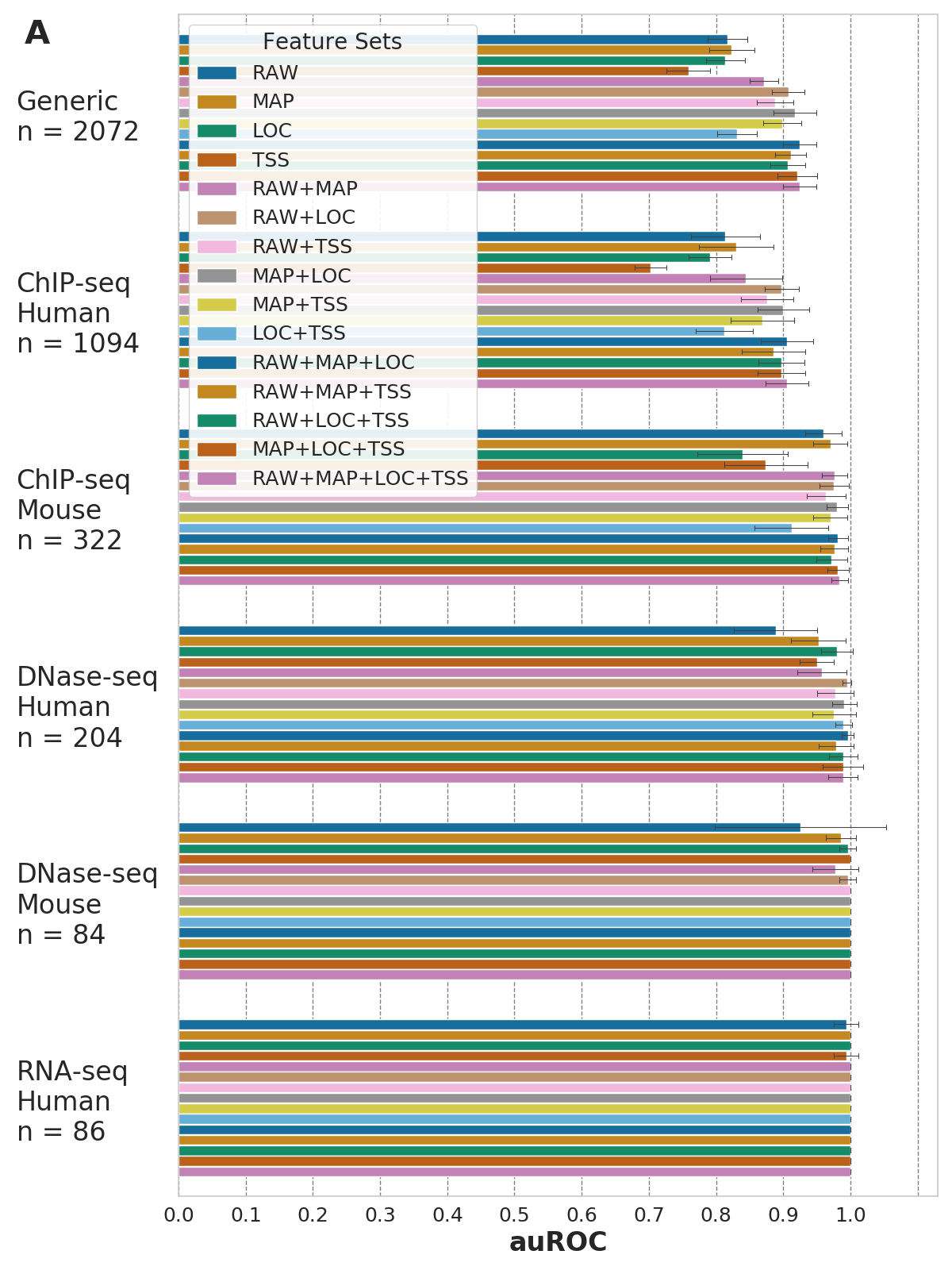


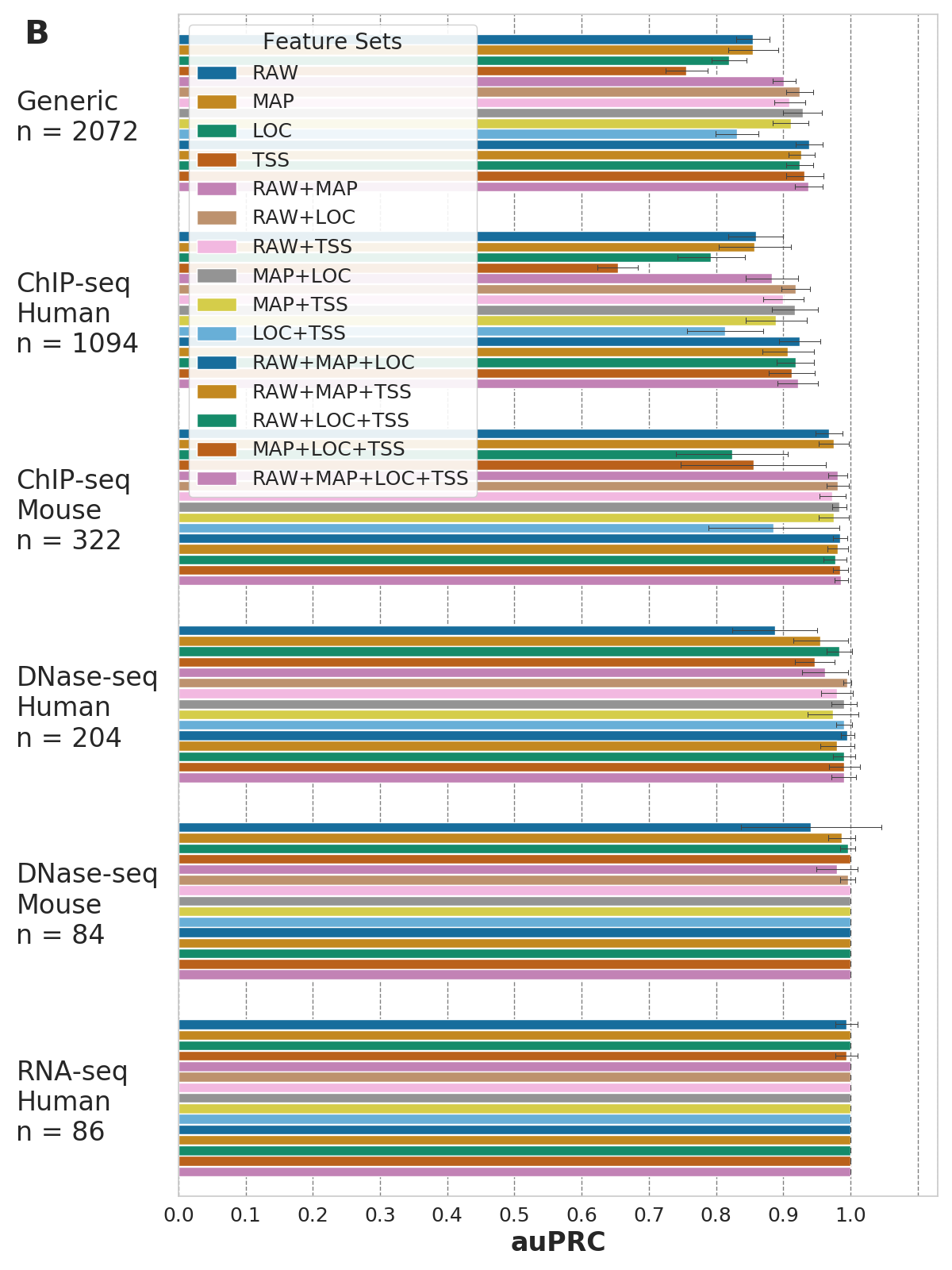


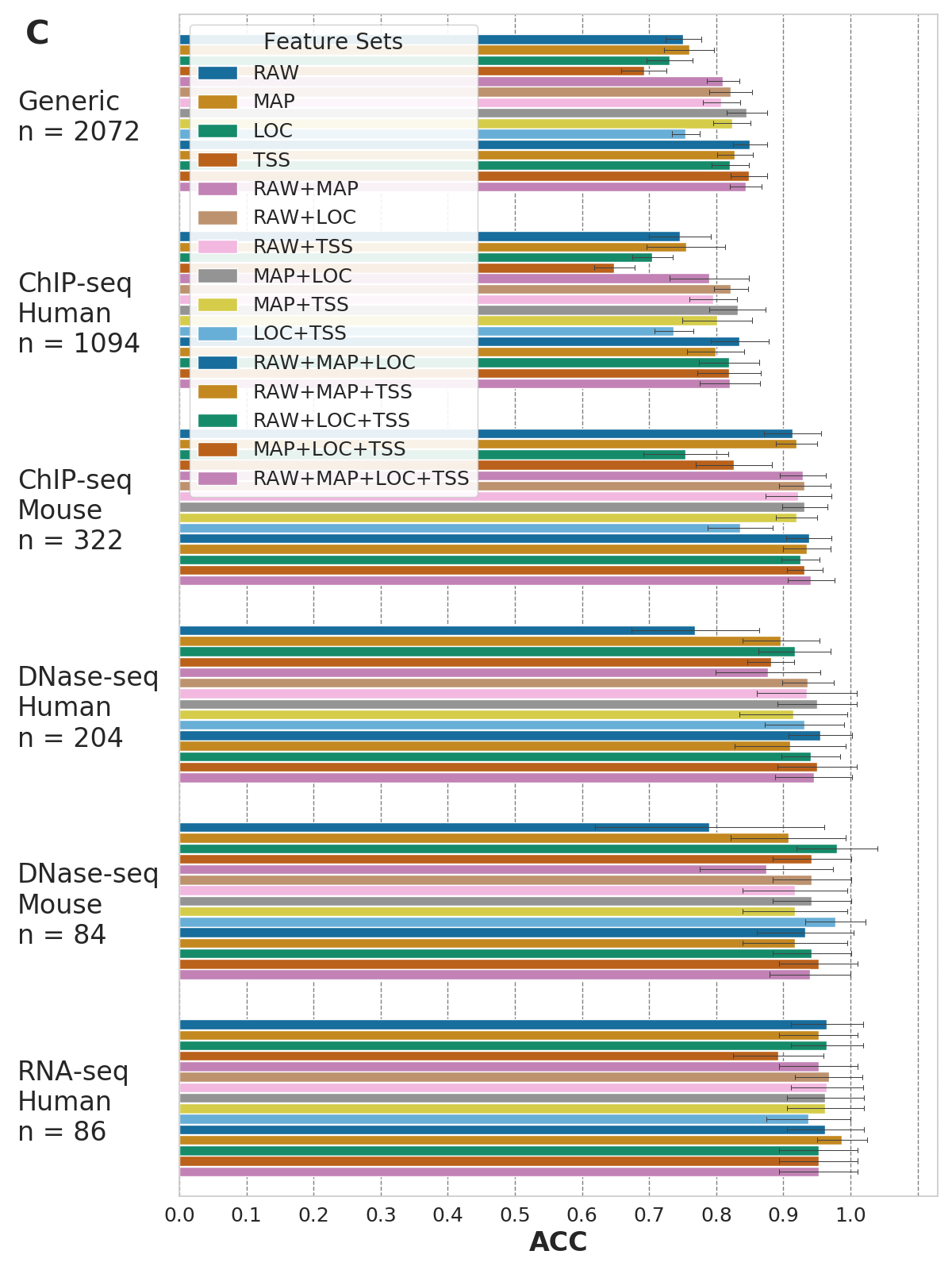


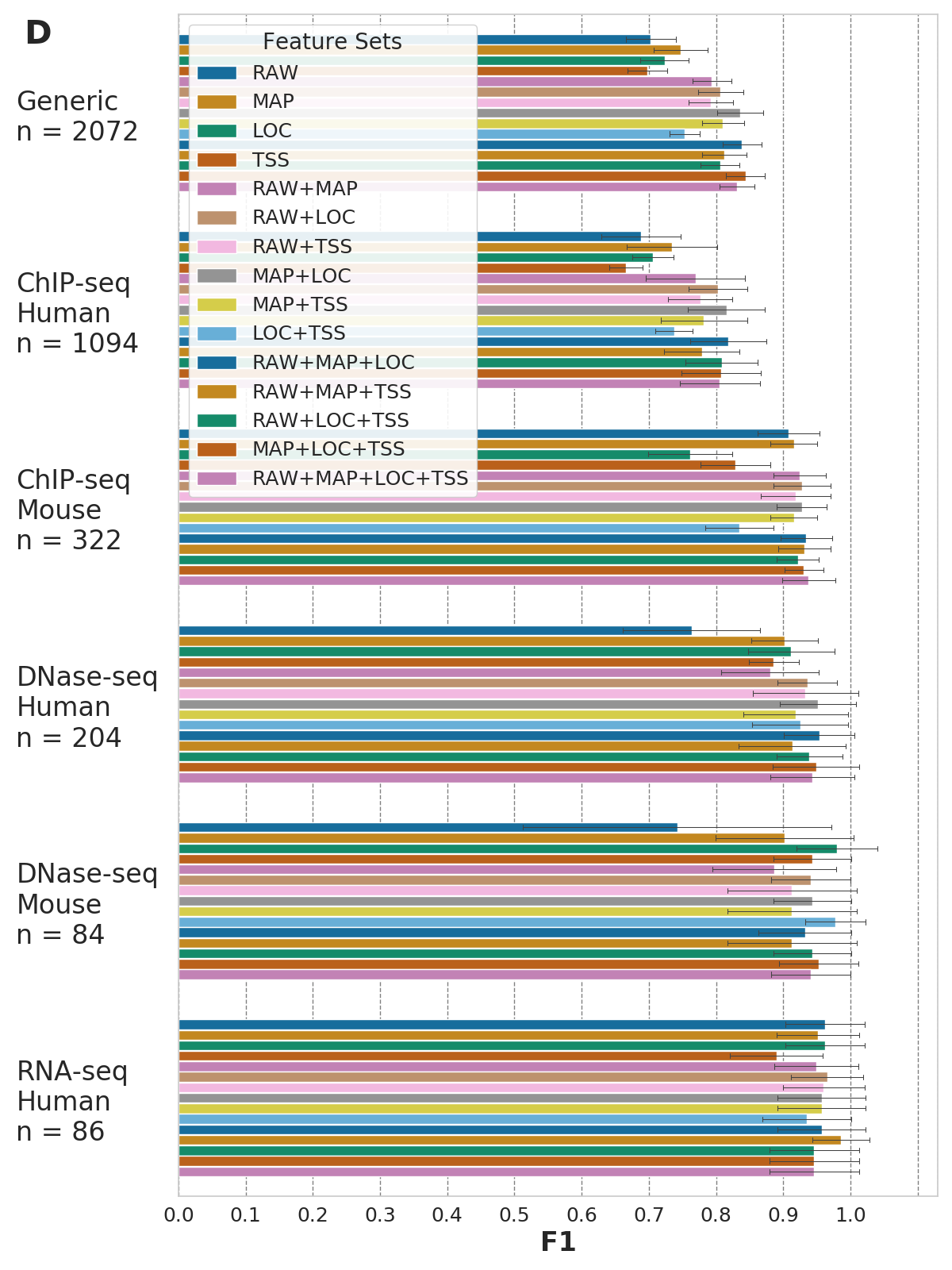


# Fig. S3 - Within-experiment benchmarks

ENCODE experiment IDs are shown along the y-axis while the files that belong to one experiment are plotted within the area indicated by the dashed lines. The x-axis shows the predictive probability of a file to be of low-quality P_low derived with the corresponding optimal specialized model indicated by the subplot titles.


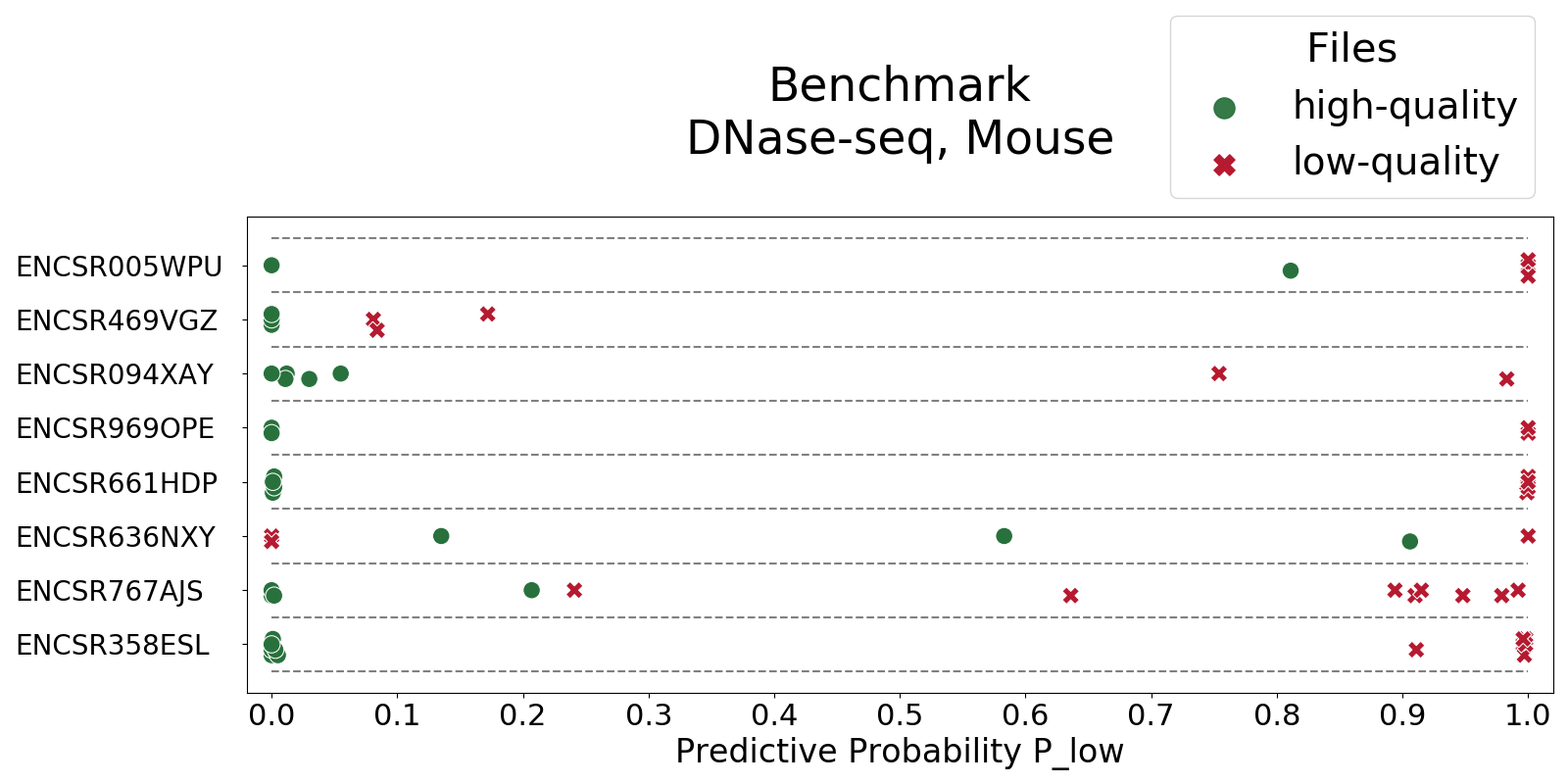


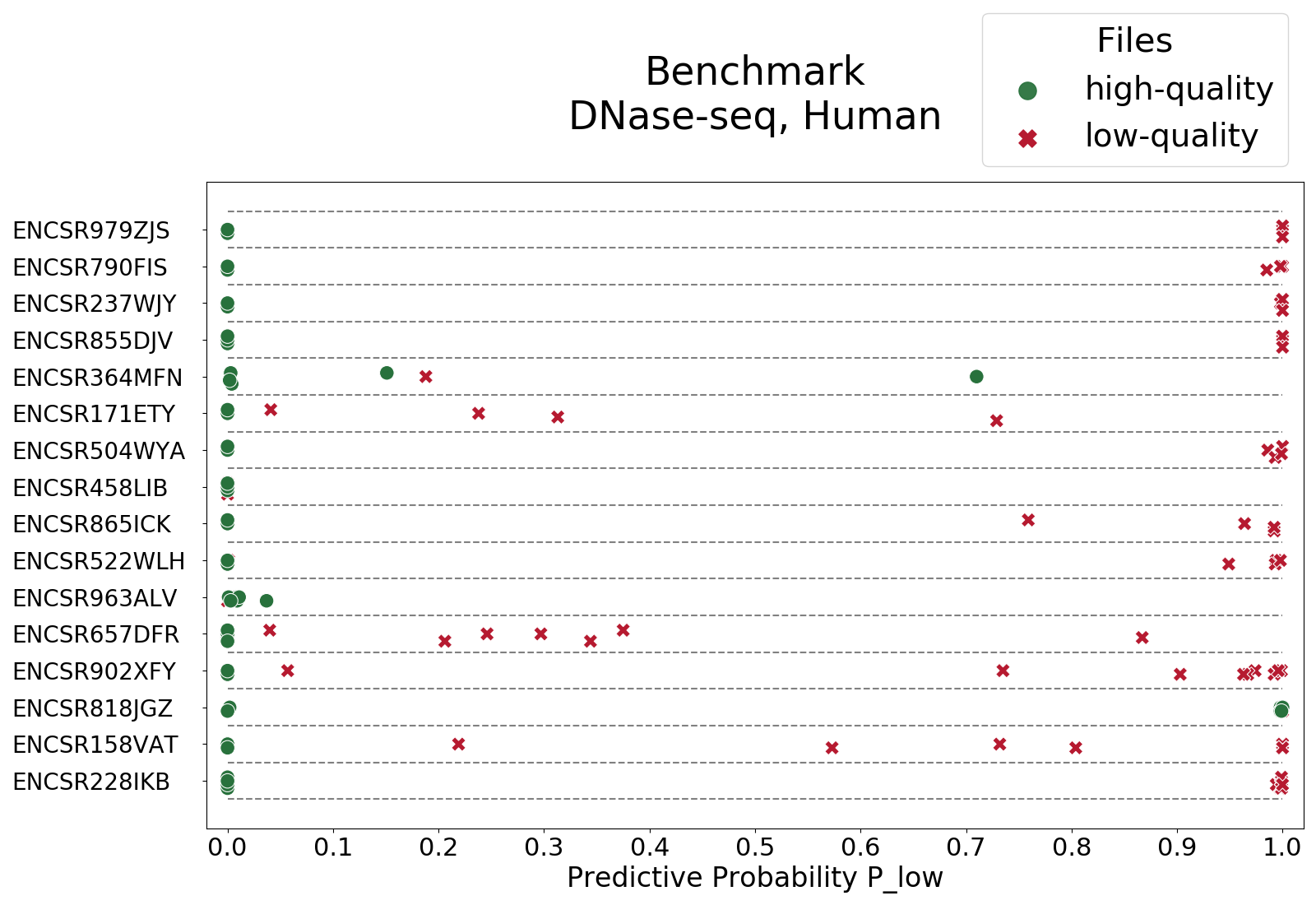


**
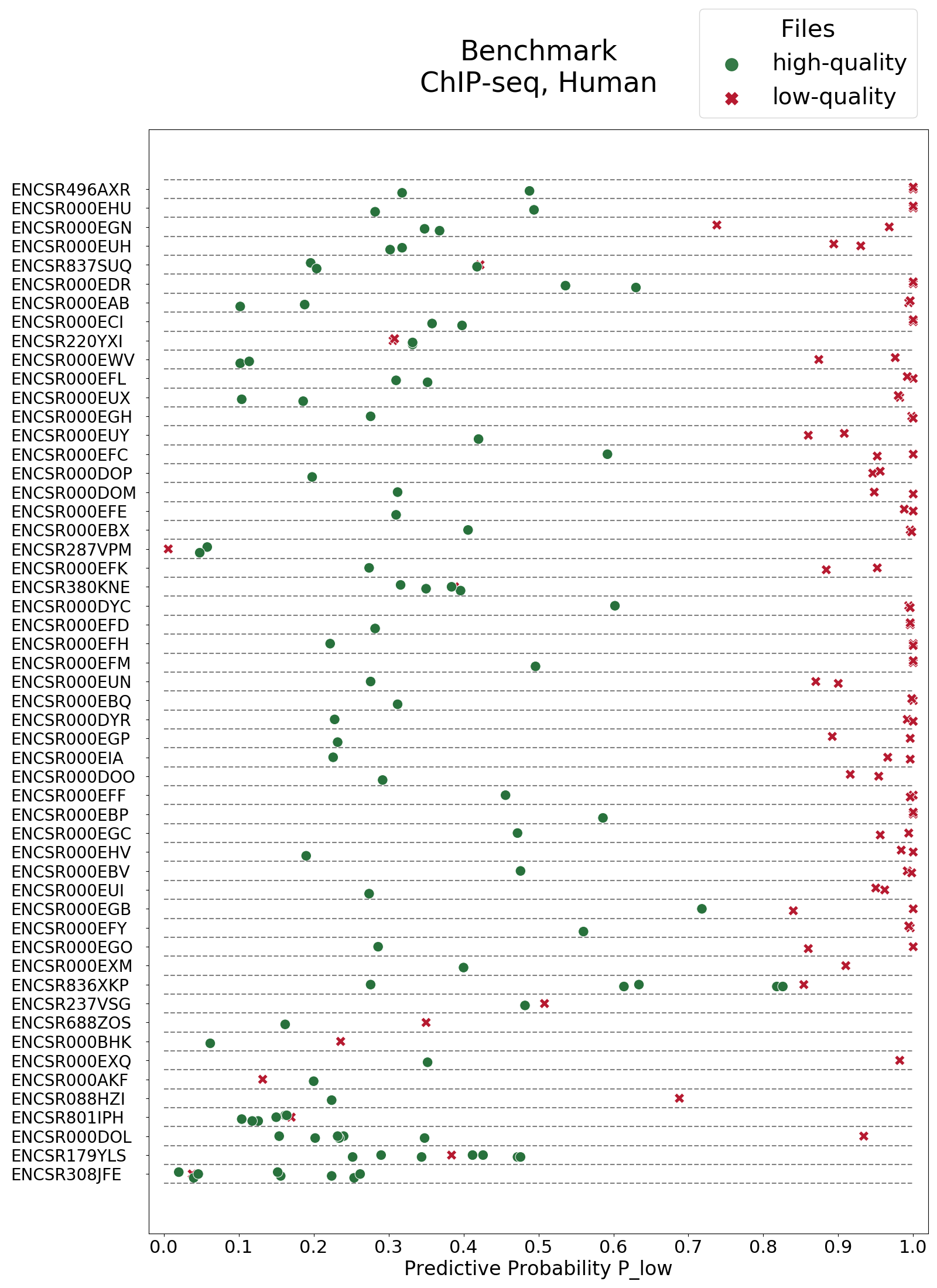
**

# Fig. S4 - Cross-species generalization

Good performances of species-specific models (optimal specialized models) in cross-species predictions demonstrate model generalization to other species. ROC curves show the classification performance for different species-assay combinations. The solid lines represent cases in which data from the same species was used to define training and testing sets. The dashed lines show the performance on cases in which the species defining the training data differs from the species defining the testing data; e.g. ”HS → MM” means the training set contains only data from human (Homo sapiens; HS), while the testing data is only from mouse (Mus musculus; MM). Legends also show values of areas under receiver operating characteristics. Feature sets: RAW (raw data), MAP (genome mapping), LOC (genomic localization), TSS (transcription start sites profile).


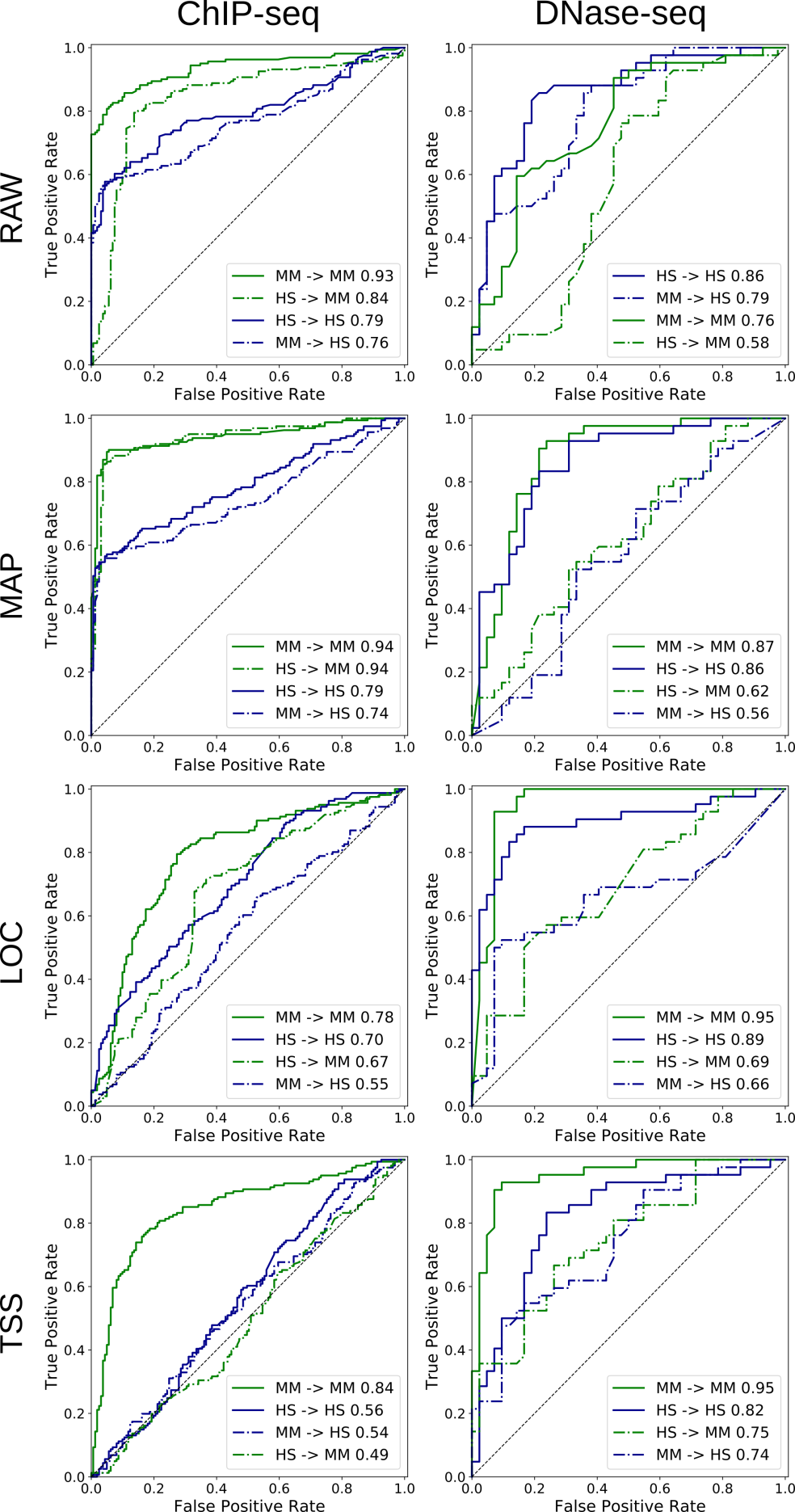


# Fig. S5 - Paired-end human ChIP-seq data subset

**A** Tuned specialized models trained on the human paired-end ChIP-seq data subset using different feature sets (y-axis) outperform one-feature predictions except with RAW features. **B** Correlation of predictive performance of the optimal generic model compared to different tuned specialized models using paired-end data demonstrates lack of bias of the generic model. For each feature set, performance of the optimal generic model (trained on all data subsets) is shown for the human paired-end ChIP-seq data subset (green bars) and compared to tuned specialized models trained for the subset only (blue bars). Error bars show standard deviations derived from 10-fold cross-validations within the grid search. Models parameters are detailed in Supplementary File 2. Feature sets: RAW (raw data), MAP (genome mapping), LOC (genomic localization), TSS (transcription start sites profile), ALL (all features).


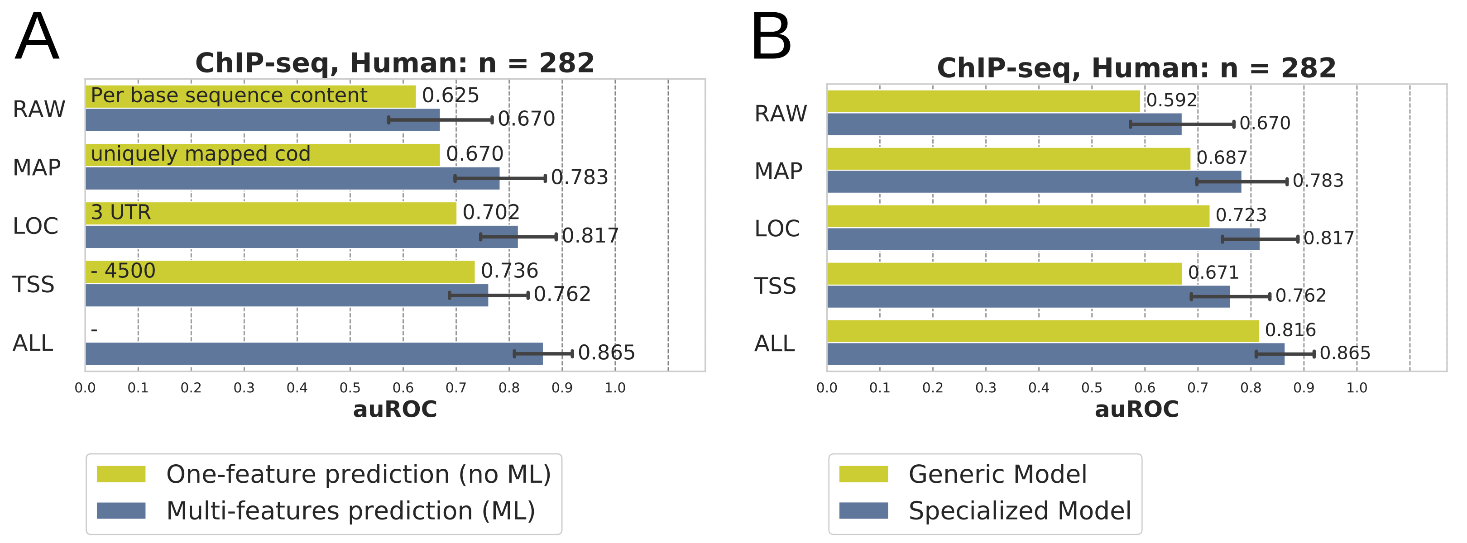


# Fig. S6 - Counts of broad peak targets in the ChIP-seq samples

Number of ChIP-seq targets related to the ChIP-seq samples of the training set that are considered broad peaks by ENCODE’s guidelines. Mouse is represented in orange and human in blue.


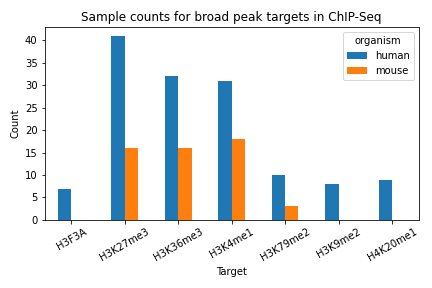


# Fig. S7 - Counts of sample names in the DNase-seq samples

Number of DNase-seq samples in the training set by cell or tissue type. Mouse is represented in orange and human in blue.


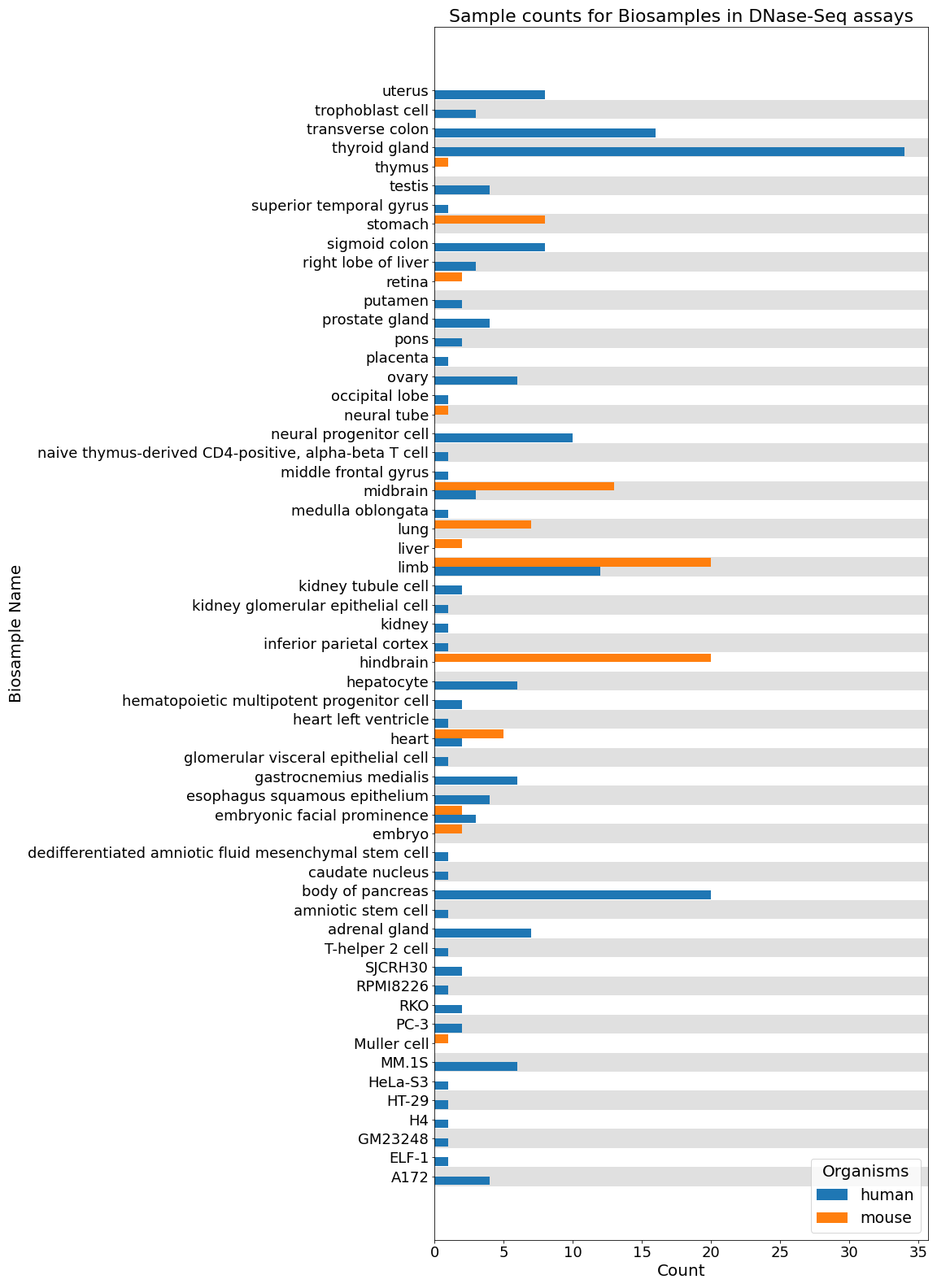


# Fig. S8 - Comparison of predictive and calibration performance

Cross-validated areas under receiver operating characteristics curves (auROC) and Brier-loss (Brier) of optimal models defined by the grid search. For each feature set, the values of two models are shown. One model selected by the highest auROC (best predictive performance) and one model selected by lowest Brier (best calibration performance) described within the x-labels. In most of the cases, the calibration can be improved slightly but it causes a small drop in the auROC as well. Only in a few cases the Brier loss can be decreased while keeping the auROC high. RAW (raw data), MAP (genome mapping), LOC (genomic localization), TSS (transcription start sites profile), ALL (all features), Generic (all data).


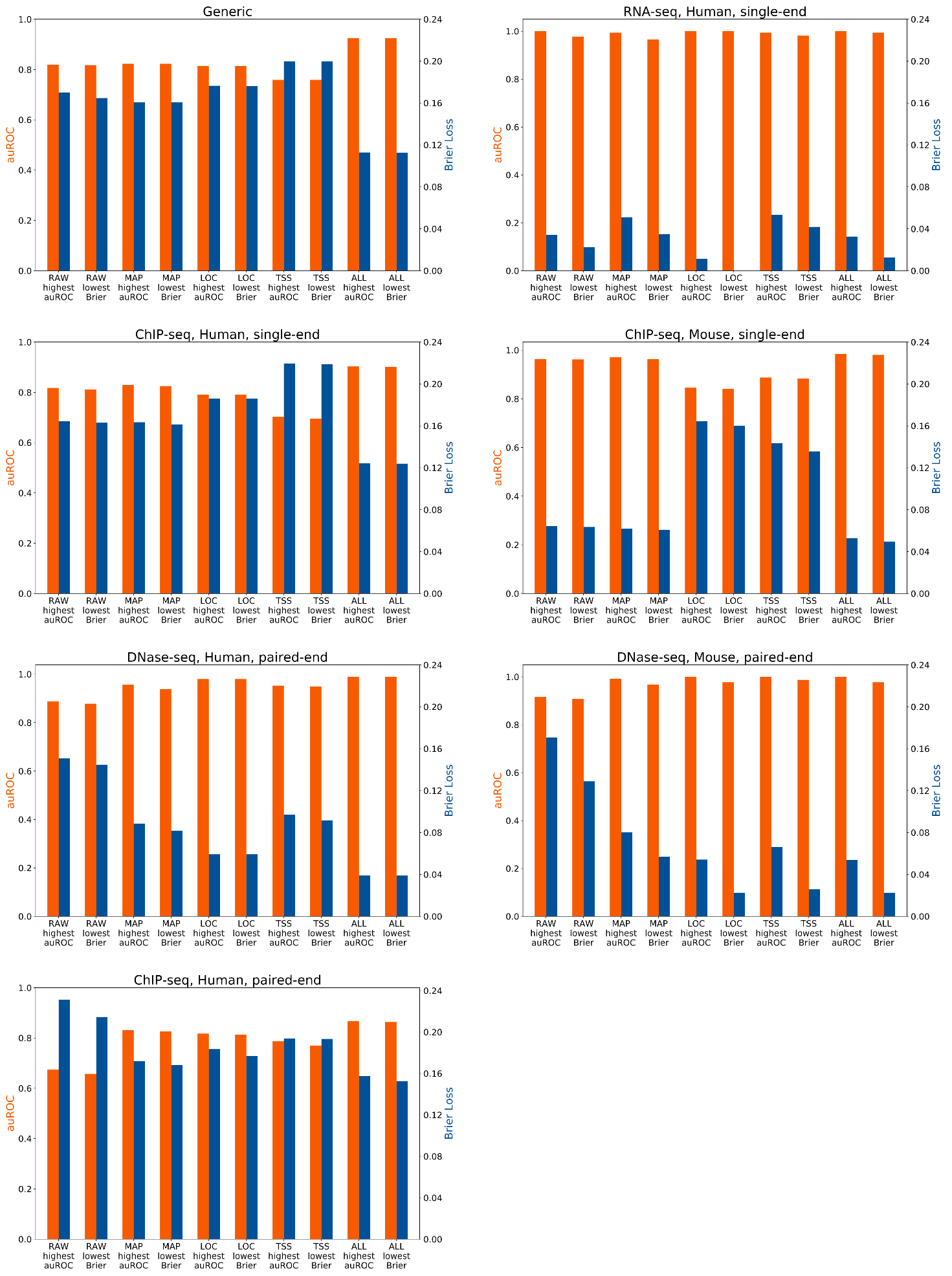


# Fig. S9 - Peak-type specific one-feature predictions

Predictive performance of each feature described by area under receiver operating characteristics curve (auROC). Column named ChIP shows values derived on all the ChIP-seq samples from human or mouse (similar to Figure 2A). Column named narrow or broad show values derived on narrow-peak or broad-peak ChIP-seq samples, respectively. These subsets are as well balanced with respect to the quality labels. Number of samples is given in parenthesis.


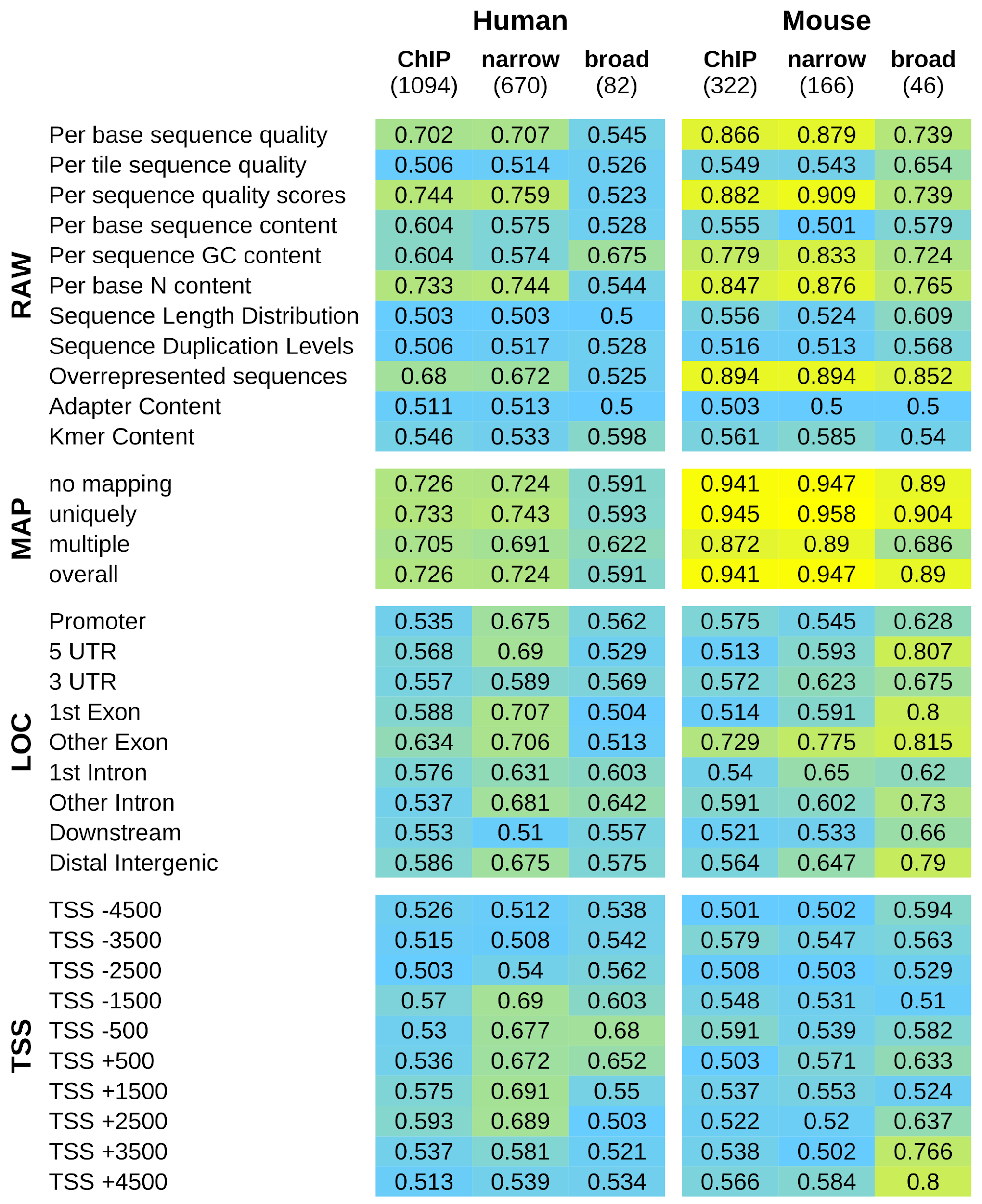


# Fig. S10 - Predictive performance of peak-type specific classification models

In order to find the optimal peak-type specific models, the grid search was applied on two more ChIP-seq subsets for human and mouse, specified by the peak type (either broad or narrow). These subsets are as well balanced with respect to the quality labels. The bars show the cross-validated area under receiver operating characteristics curve (auROC) of the models tuned by the grid search. Models considered were the following: peak-type specific models (narrow or broad), the optimal generic model (trained on the full dataset; Generic Model) and the human or mouse optimal specialized model (ChIP-seq model; trained on all human or all mouse ChIP-seq samples, respectively). The evaluation was restricted only to the samples from the peak-type specific subsets as indicated by the four subtitles of the subplots. Especially, on the human broad-ChIP-seq dataset, the peak-type specific model improves the predictive performance drastically. For the remaining subsets, the generic model is outperformed by the ChIP-seq model, which is comparable to the peak-type specific model. Feature sets: RAW (raw data), MAP (genome mapping), LOC (genomic localization), TSS (transcription start sites profile), ALL (all features).


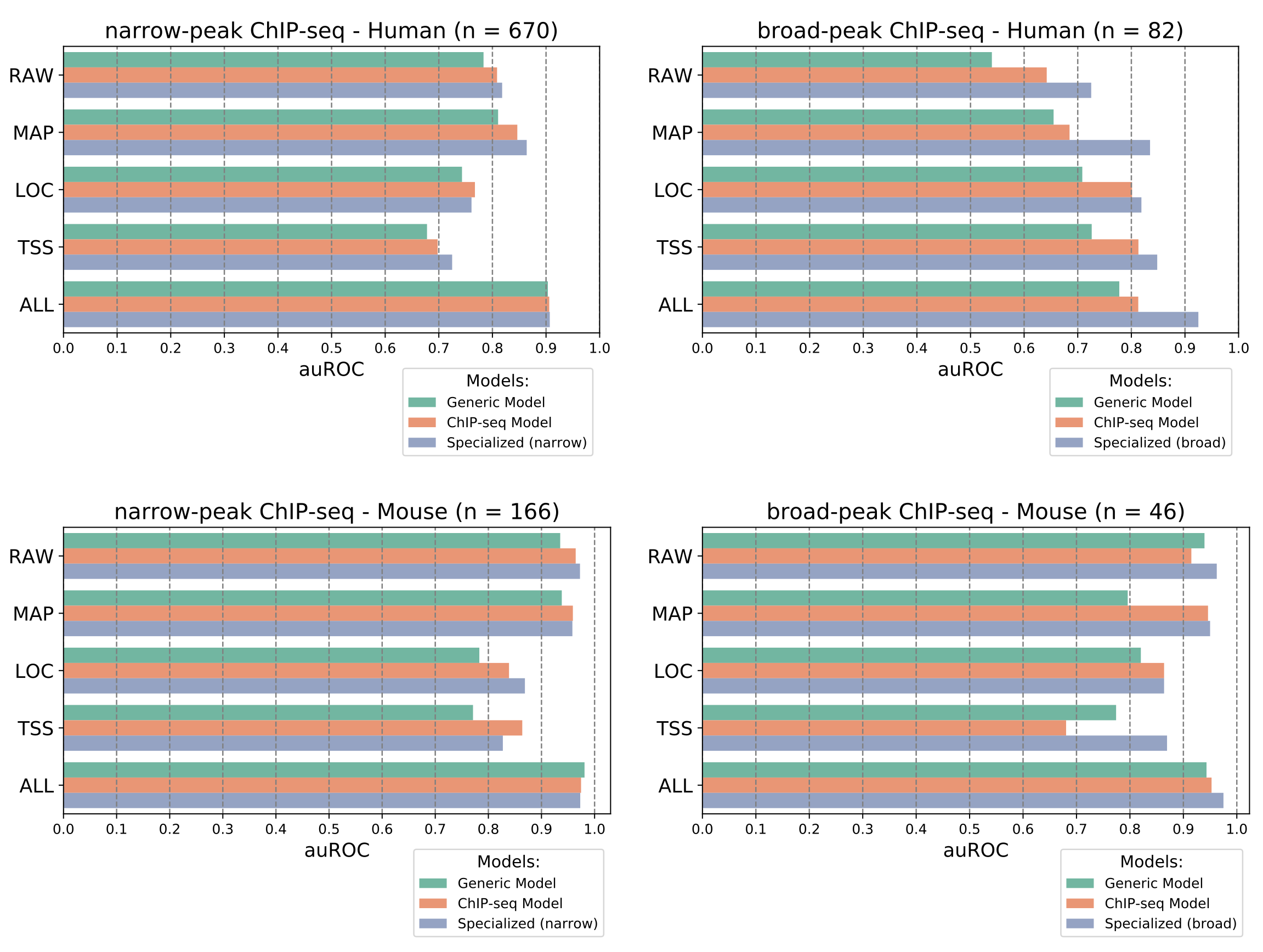


# Fig. S11 - Cross validated predictions of the optimal generic model across most frequent ChIP-seq protein targets

**A)** Results of the optimal generic model trained on all features are detailed for the most frequent ChIP-seq protein targets in the training set. **B)** Results of the generic model tuned on RAW and MAP features only. No bias could be observed in A or B for particular proteins. x-axis: low-quality probability. y-axis: number of NGS samples in the training set. Status: manual quality annotation from ENCODE. Feature sets: RAW (raw data), MAP (genome mapping).

**A.**


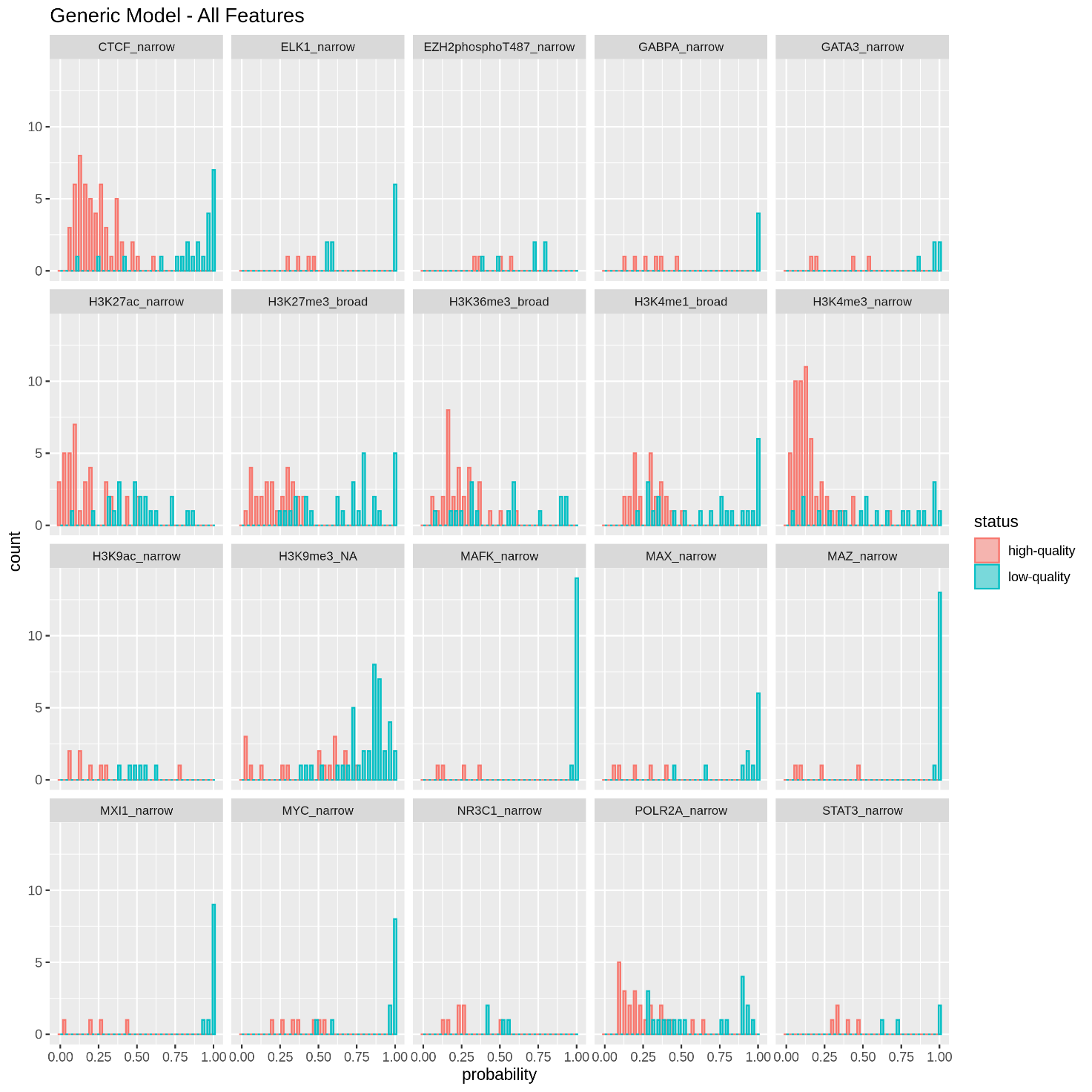


**B.**


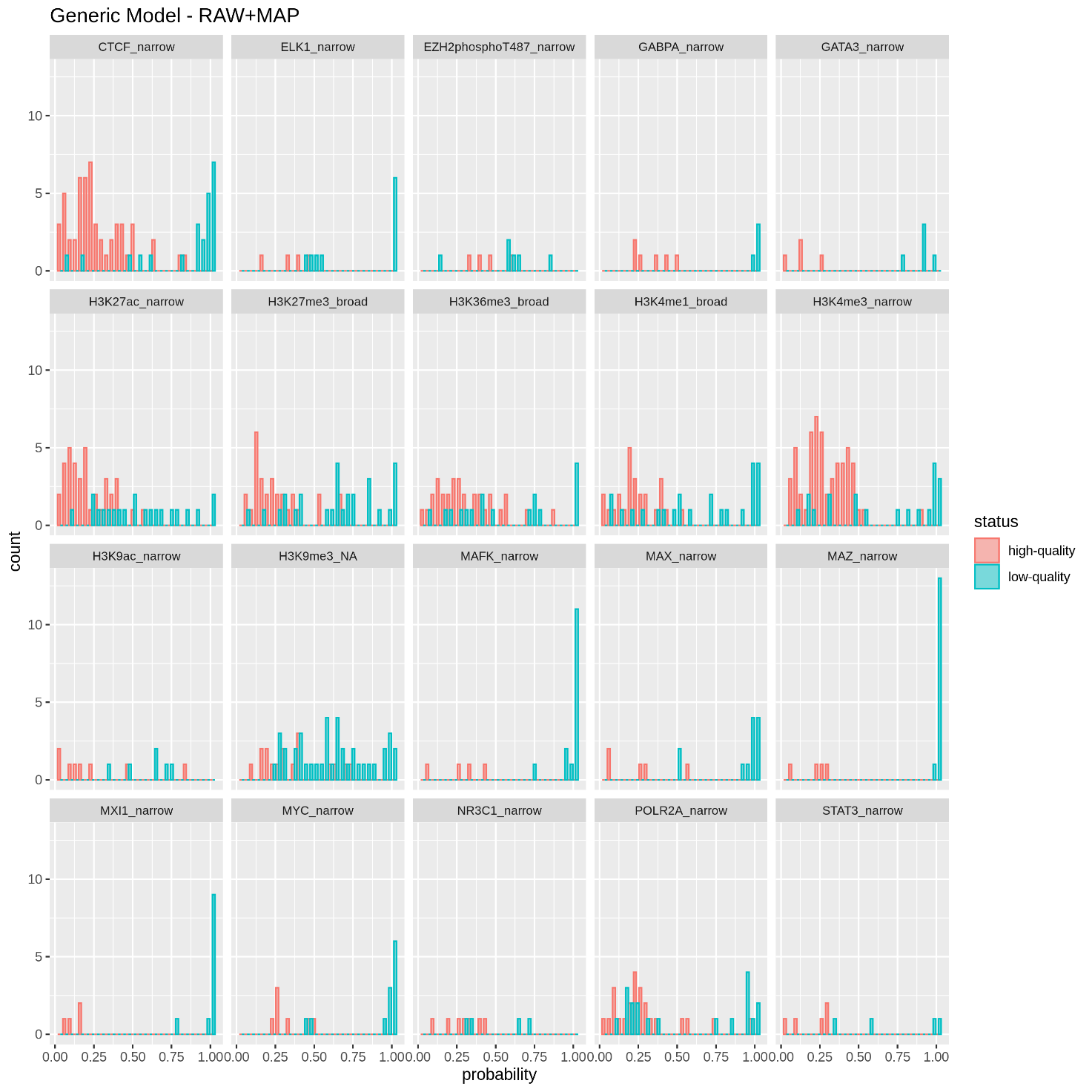


# Fig. S12 - External validations (RNA-Seq)

Principal component analysis of 6 gene-expression datasets from the GEO database including human control and disease samples. For each dataset, a first principal component analysis (PCA) including all samples is shown on the left-hand side, and a PCA excluding outliers is shown on the right-hand side. Either only outliers (on 1^st^ PCA) or all samples (2^nd^ PCA) are annotated with their sample ID and low-quality probability given by the optimal generic model. For testing purposes, 2 samples associated with highest low-quality probability were automatically defined as outliers for each group of each dataset although they may not be of real low quality. Dunn: Dunn’s index (the higher, the better the clustering). sALS: sporadic amyotrophic lateral sclerosis.


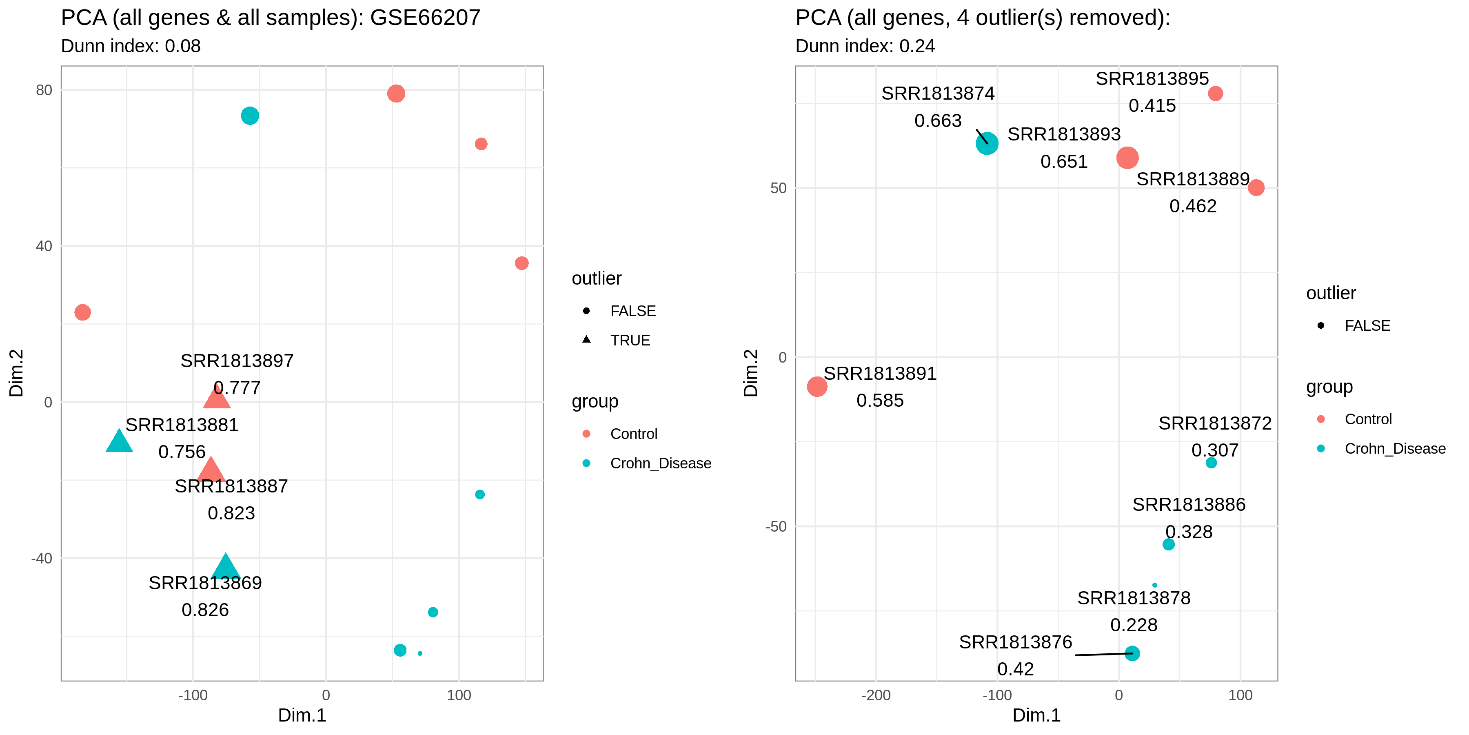

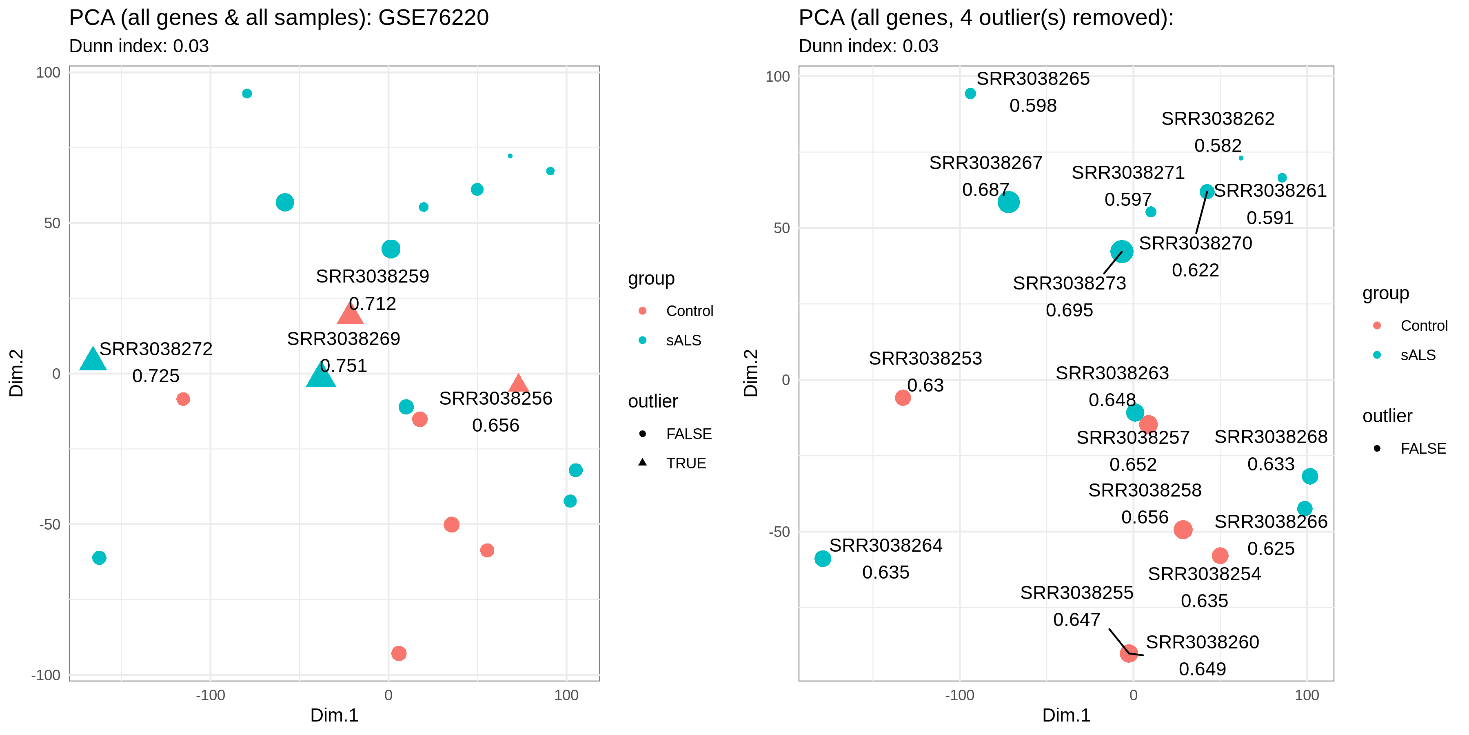

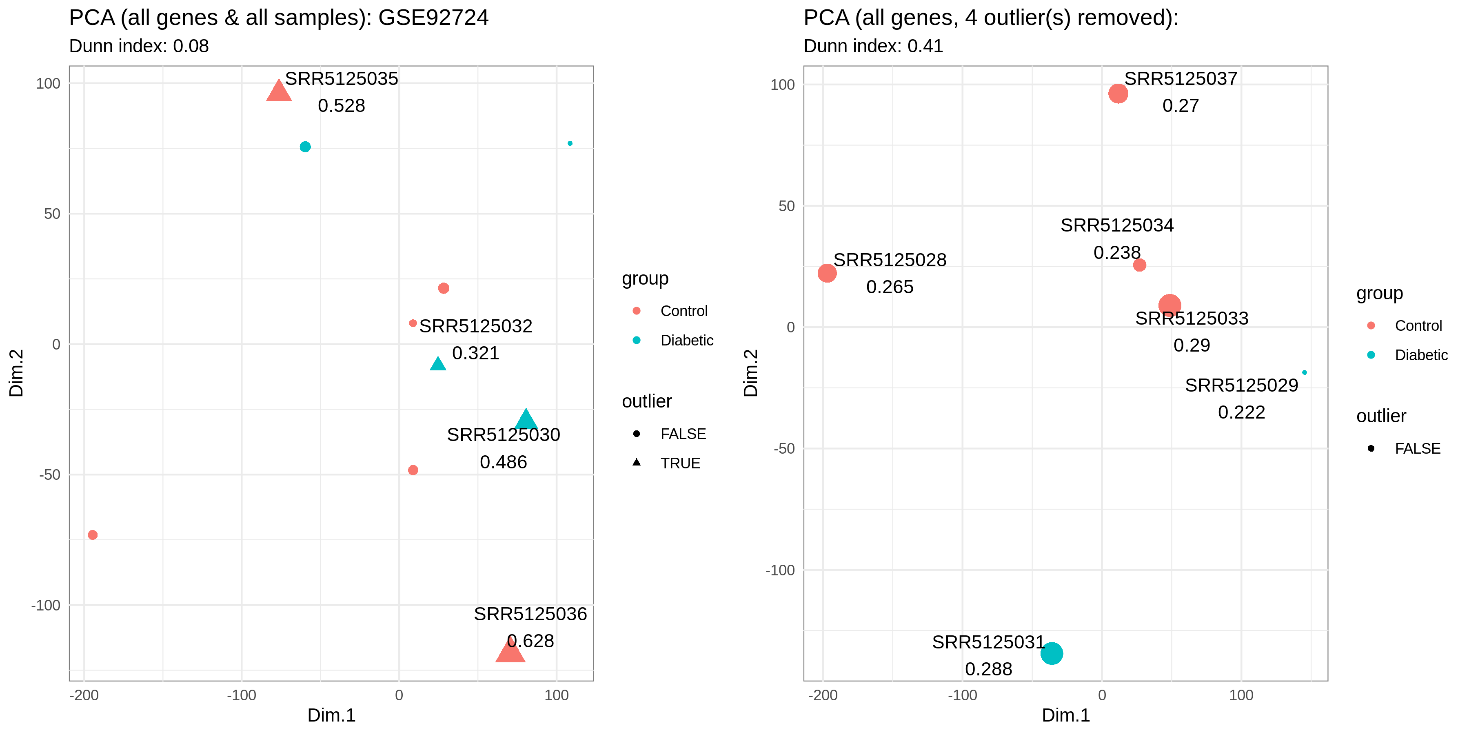

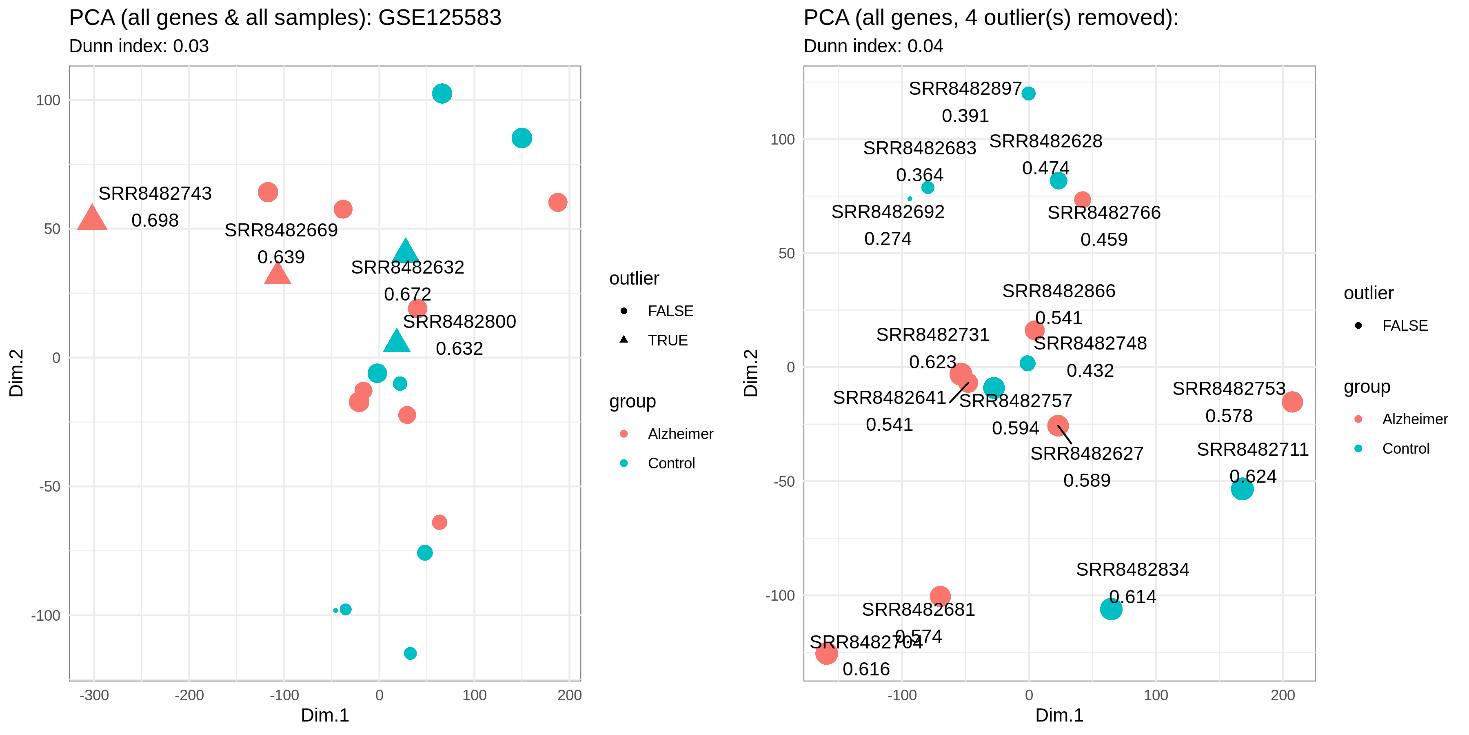

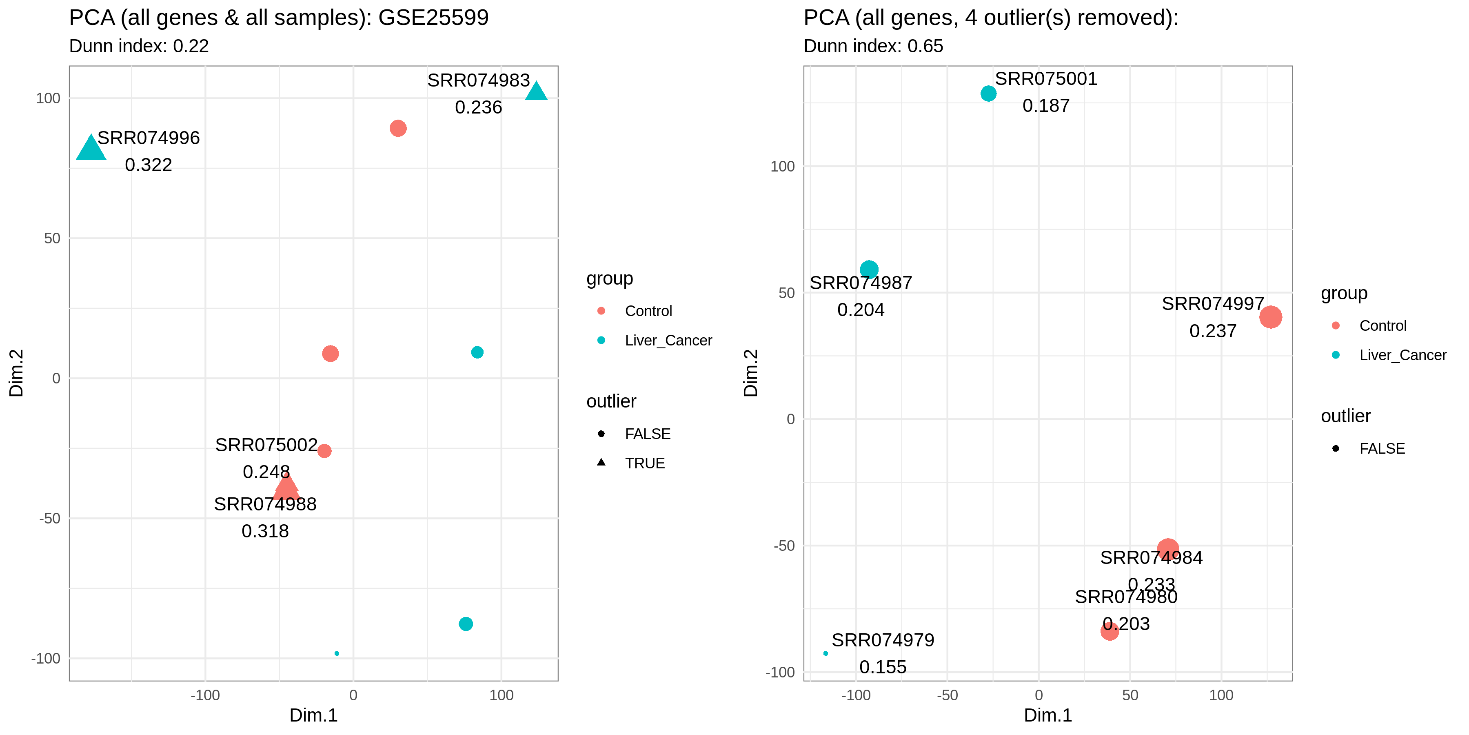

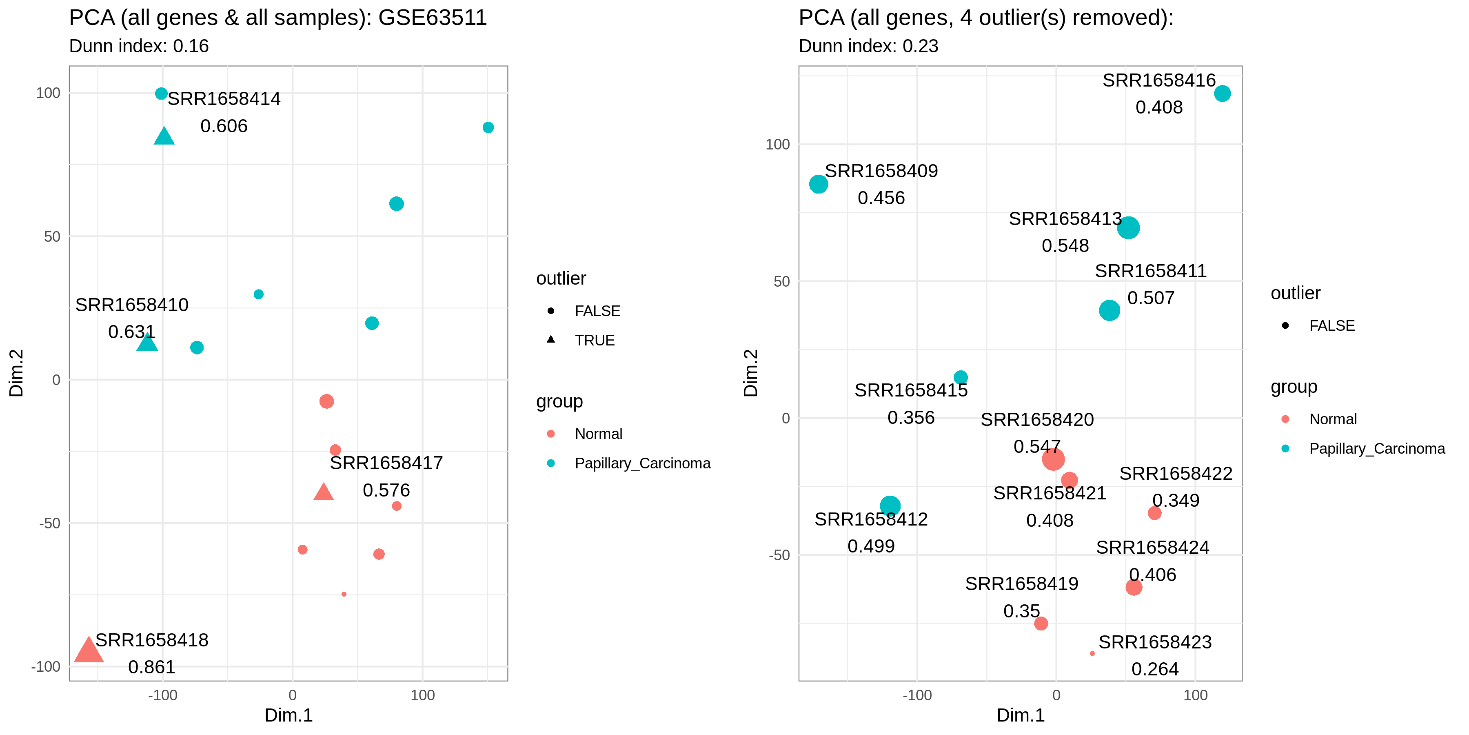


# Fig. S13 - Independent validation on Cistrome’s datasets

Quality prediction of ATAC-Seq, ChIP-seq and DNase-seq samples from the Cistrome database. We retrieved the data from the SRA database. We derived the low-quality probabilities for a total of 610 samples from 32 datasets. Cistrome quality metrics were downloaded from the Cistrome data portal. We could compare the low-quality probabilities given by the optimal generic or specialized models for all samples that were annotated in Cistrome with at least 4 quality metrics (maximum number was 5 metrics). If a metric was lower than the cutoff provided by the Cistrome’s guidelines, it was considered a bad quality control (QC) flag, a good QC flag otherwise. Because not existing for particular data subsets, the optimal specialized models were replaced by the optimal generic model. Yet, the optimal specialized models applied on ATAC-seq samples were actually the models trained on DNase-seq data (see Methods for more details). r_generic: Pearson’s correlation coefficient of the optimal generic model. r_specialized: Pearson’s correlation coefficient of the optimal specialized models.


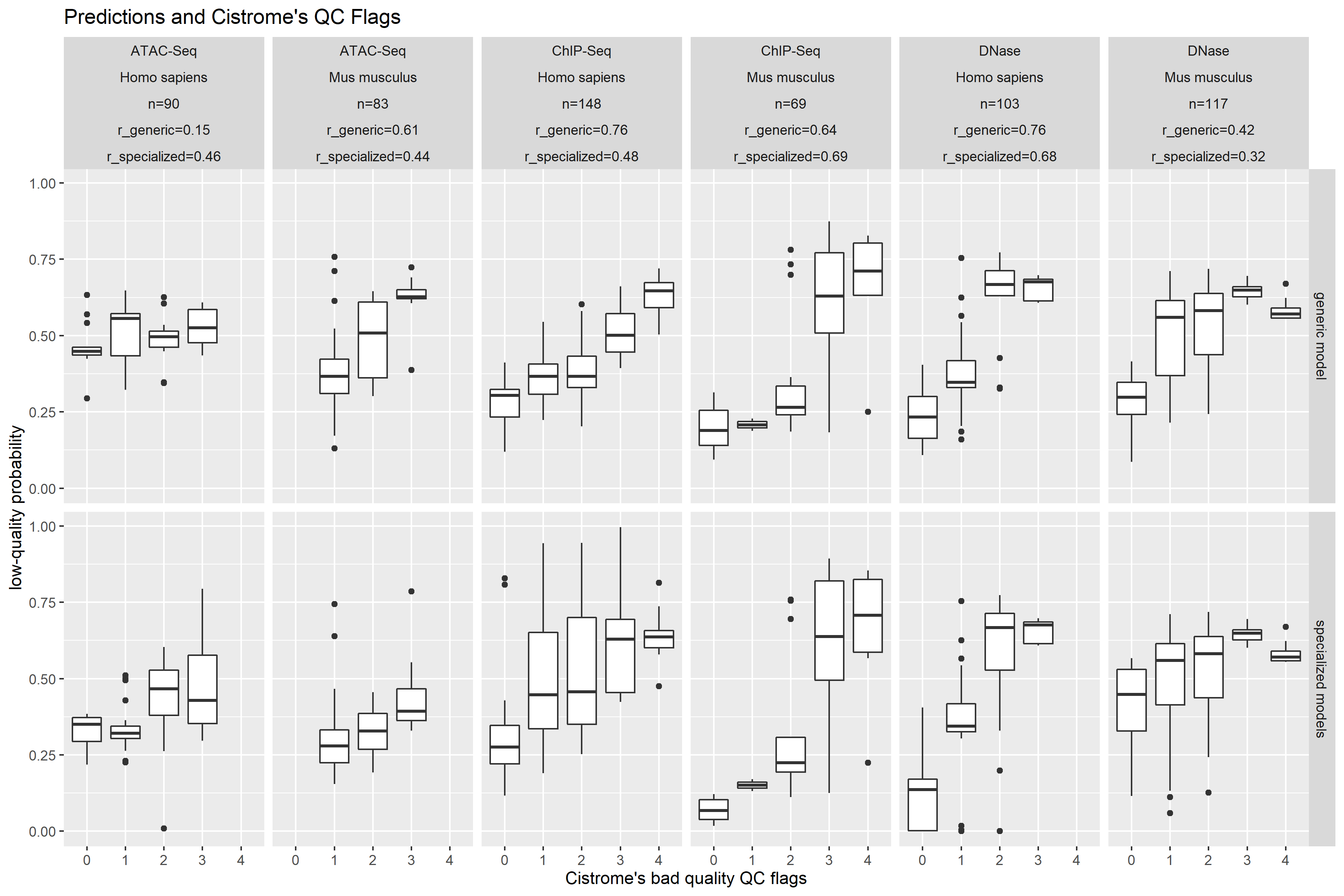


# Fig. S14 - ENCODE guidelines and status

ENCODE 3 guidelines related to minimal number of aligned reads, uniquely mapped reads and usable fragments are compared to observed values in the training set samples. Samples are compared by quality annotation from ENCODE (A,C,E) or from the optimal generic model (B,D,F; low-quality if low-quality probability<0.5, high-quality otherwise). Only samples produced within the ENCODE 3 period were used (September 2012–January 2017). ENCODE guidelines are documented at https://www.encodeproject.org/about/experiment-guidelines. Dashed lines show the minimal numbers from the guidelines: 30M aligned reads for RNA-seq (red dashed line), 45M aligned reads for H3K9me3 ChIP-seq (blue dashed line), 20M usable fragments for DNase-seq (red dashed line), 20M usable fragments for narrow-peak ChIP-seq (red dashed line) and 45M usable fragments for broad-peak ChIP-seq (blue dashed line).


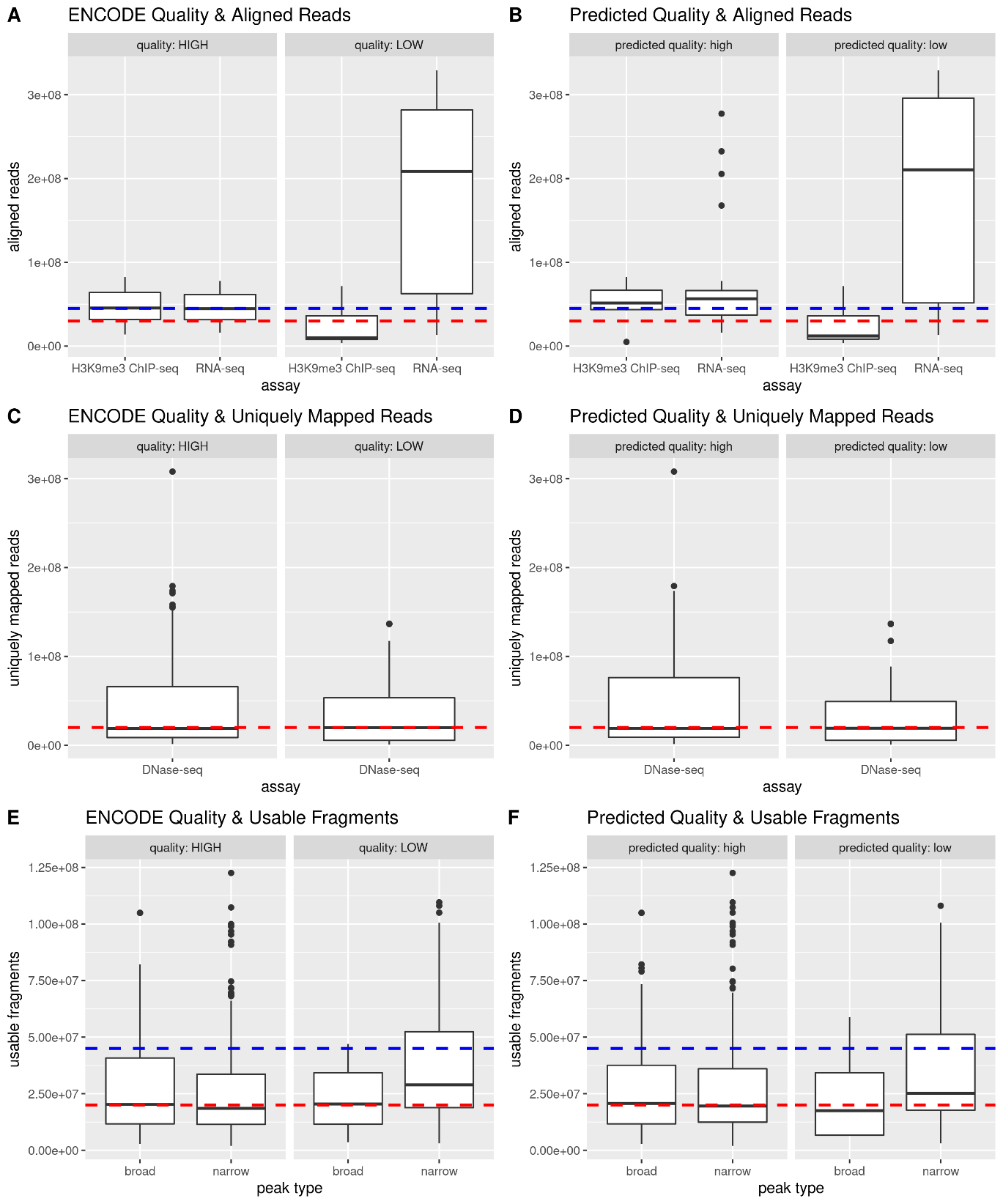

Supplement: Supplementary file 1 — Additional file 1: Figure S1. Statistical guidelines computed on the ENCODE files selection. Figure S2. Predictive performance of tuned machine learning models. Figure S3. Within-experiment benchmarks. Figure S4. Cross-species generalization. Figure S5. Paired-end human ChIP-seq data subset. Figure S6. Counts of broad peak targets in the ChIP-seq samples. Figure S7. Counts of sample names in the DNase-seq samples. Figure S8. Comparison of predictive and calibration performance. Figure S9. Peak-type specific one-feature predictions. Figure S10. Predictive performance of peak-type specific classification models. Figure S11. Cross validated predictions of the optimal generic model across most frequent ChIP-seq protein targets. Figure S12. External validations (RNA-Seq). Figure S13. Independent validation on Cistrome’s datasets. Figure S14. ENCODE guidelines and status. [file 13059_2021_2294_MOESM1_ESM.docx]
