# Supplementary material for: seqQscorer: automated quality control of next-generation sequencing data using machine learning
Source: Genome Biol. 2021 Mar 5;22:75. doi: 10.1186/s13059-021-02294-2 (PMC7934511; doi:10.1186/s13059-021-02294-2)
Supplement: Supplementary file 8 — Additional file 8. Review history. [file 13059_2021_2294_MOESM8_ESM.docx]

Review History

**First round of review**

**Reviewer 1**

**Were you able to assess all statistics in the manuscript, including the appropriateness of statistical tests used?**

Yes, and I have assessed the statistics in my report.

**Were you able to directly test the methods?**

Yes

**Comments to author:**

The authors of this manuscript aim at developing machine learning methods to automatically predict quality of NGS data. It is indeed a need in the sequencing data preprocessing and analysis community. While I think it may be a good idea, I have the following concerns about methodology and data.

Majors:

1. Existing work has shown that MLP with dropout regularization can improve prediction performance (https://jmlr.org/papers/v15/srivastava14a.html). Thus, I suggest the authors explore this option in their comparisons.

2. The experimental observations are restricted to ENCODE data which may be biased as certain protocols were applied in ENCODE to annotate data quality. Thus, I am curious about the performance of models trained on ENCODE and tested on data sets other than ENCODE (e.g. GTEx etc.). I understand that there may be no comparable data sets as big as ENCODE, but a test on a few hundreds good and bad files should be sufficient to show the generalization capacity of the methods. The last section (Application to independent diagnostic studies) in the current version is insufficient to convince readers about generalization.

3. Regarding feature selection, why was chi-square used? Since correlations between features can't be considered in univariate feature selection methods, stability selection can be considered as a better choice (https://rss.onlinelibrary.wiley.com/doi/10.1111/j.1467-9868.2010.00740.x).

Minor:

2,642human -> 2,642 human

**Reviewer 2**

**Were you able to assess all statistics in the manuscript, including the appropriateness of statistical tests used?**

Yes, and I have assessed the statistics in my report.

**Were you able to directly test the methods?**

No

**Comments to author:**

Assessing the quality of sequencing data is a critical task. Many methods and metrics have been proposed to tackle this task. The authors developed an automated pipeline that can predict the quality from the raw data. They have analyzed 2642 human and mouse raw sequence data annotated by the ENCODE consortium using 10 machine learning methods with a grid search of parameters. They assessed the quality prediction of individual features and multiple features. This study provides a comprehensive benchmark of features and models to predict ChIP-seq, DNase-seq and RNA-seq data quality.

1. The computational procedure needs to be better explained, such as which machine learning is used for prediction (see below).

2. ENCODE DCC provided guideline for each data set with recommended sequencing depth and uniquely mapped reads. It would be useful to discuss these features and how useful they are for predicting data quality. It'd be helpful to improve the guideline.

3. Related to the above point, the authors should summarize the analysis and recommend a generalized model with specific algorithm and parameters for quality prediction that can be applied in practice. This is what readers most want to know but it is lacked in the manuscript.

4. ChIP-seq data for narrow and broad peaks can be very different in terms of quality assessment. It is critical to separately evaluate broad peak ChIP-seq data and check which features are predictive.

5. Two important data types, ATAC-seq and DNA methylome, are not included in the analysis. For a comprehensive analysis study, they should be included.

Minor issues:

1. It is unclear how the one-feature quality prediction was performed. How the AUCs reported in Fig. 2 are obtained is not described. It seems no machine learning model is used. Is it only enrichment of each feature shown in Fig. S1 used to predict data quality?

2. For one-feature prediction, human and mouse DNAse-seq diverge more than the ChIP-seq data, particularly for the MAP and TSS features. Any reason for that?

3. For multi-feature prediction in Fig. 2B, it is also unclear which machine learning is used and how the AUC was obtained.

4. The same problem for Fig. 3, it is unclear which models are used to calculate the probability.

5. Fig. 1B, many data types have less than 200 files. It'd be useful to put the file number next to the bars to indicate how many files are available.

**Reviewer 3**

**Were you able to assess all statistics in the manuscript, including the appropriateness of statistical tests used?**

Yes, and I have assessed the statistics in my report.

**Were you able to directly test the methods?**

No

**Comments to author:**

The authors described a machine learning-based sequencing data QC tool. The method uses input from fastQC, Bowtie2 alignment statistics, and extra location distribution features and is trained on the released vs revoked data from ENCODE. This is an interesting approach that is potentially widely applicable. The machine learning approach could allow inexperienced user to learn from expert decisions and established a decision boundary for low quality sample. However, more evaluations and potential improvement are needed to demonstrate these potentials are practical, especially on external datasets not used in model training, as detailed below.

Major points:

1. External validation - a major concern for this study is that it heavily relies on training and validating on a specific dataset that may contain certain biases. The study on external datasets is small-scale and inadequate for demonstrating its performance on external data with confidence. A more extensive study on external datasets is needed (e.g. on Roadmap Epigenomics if it is not overlapping with the training data). Even though hand curated dataset quality labels are generally not available, the majority of the published should have passed quality control. The model predictions should reflect that and the datasets classified as the lowest quality may be manually inspected for their quality issues.

2. Evaluation. It is unknown whether the predicted probabilities are well calibrated (i.e. are 90% positive label observed when prediction is 0.9 on training data)? This is important as a user will need to rely on the score provided by the software to make QC decision with necessarily any reference to compare with. The method's performance with different thresholds or a recommended threshold (TPR, FPR, Precision) should also be presented to guide the usage.

The authors mentioned that the predicted probabilities may not be comparable across models and studies. I appreciate their honesty in bringing this up, and I think this is an important issue that need to be addressed as it otherwise greatly reduced the usefulness of this software. If the probabilities are not comparable across models, the model output may be calibrated with isotonic regression or logistic monotonic spline regression on the training data. If the probabilities are not comparable across studies, then it needs to be addressed how the user should interpret the results, without relying on the users training their own models which is not realistic most of the time.

3. Features. Training on ENCODE released vs revoked data is an interesting idea. As these labels are manual in nature, and the ENCODE data generators / curators most likely used established QC metrics and tools in making these decisions, the machine learning method should recapitulate these decisions as closely as possible. Therefore, the machine learning approach should have a comprehensive coverage of existing QC metrics in its input features. Even though the authors included a set of clearly informative features, including FastQC metrics, it is unclear whether this set of features covers metrics used on data type-specific QC tools for RNA-seq (e.g. RSeqQC ) and ChIP-seq / DNase-seq QC( e.g. ChiLin).

Another feature-related concern is the inclusion of LOC and TSS features for ChIP-seq. As ChIP-seq for different targets are expected to have highly distinct location distributions, would inclusion of these features bias against / toward particular types of ChIP-seq targets?

Minor:

1. Is there any source / references that explain the reasons for the revocation status of ENCODE data and that the reason for revocation is mostly due to low-quality?

2. Machine learning parameters. The number of parameters were mentioned in the text but no specific descriptions about what parameters were tried are provided.

**Authors Response**

**Point-by-point responses to the reviewers’ comments:**

REVIEWER #1:

The authors of this manuscript aim at developing machine learning methods to automatically predict quality of NGS data. It is indeed a need in the sequencing data preprocessing and analysis community. While I think it may be a good idea, I have the following concerns about methodology and data.

MAJORS:

1. Existing work has shown that MLP with dropout regularization can improve prediction performance (https://jmlr.org/papers/v15/srivastava14a.html). Thus, I suggest the authors explore this option in their comparisons.

*Dropout layers can have a positive impact on the model performance, especially due to their capability of preventing the model from overfitting. Since the MLP classifier from scikit-learn does not implement dropout regularization, we performed a test using a keras installation to explore the potential of dropout regularization on our data. For this analysis we used the generic (complete) dataset and all feature sets for which the optimal generic model performed the best in our grid search (area under ROC-curve (auROC) = 0.925 with a Random Forest configuration). This dataset has the largest sample size and should be most appropriate for deep learning algorithms.*

*For each scikit-learn MLP setting we used in the grid search, we ran a comparable setting within the keras library and additionally ran the same keras MLP with a dropout layer for 7 different dropout rates (0.3, 0.4, …, 0.8, 0.9). Each keras MLP was evaluated by auROC on the same folds from the ten-fold cross-validation we used for the grid search. Afterwards, we compared the auROC values and searched for the highest auROC achieved and also the maximum improvement gained by running the MLP with a dropout layer. Additionally, leveraging the parallel computations from the underlying tensorflow package, we trained deeper MLPs (up to 5 hidden layers).*

*Eventually, we saw that a keras MLP with dropout regularization can improve the auROC by 0.043 compared to the scikit-learn MLPs. However, the maximum auROC achieved by the best keras MLPs was 0.894, which is slightly higher than the one achieved by the sklearn MLPs (0.890), but still lower than the one achieved by the best Random Forest setting.*

*From this analysis we conclude that the dataset itself might be more suitable for a decision tree-based classifier and the MLP is in this case not able to outperform it. However, for future work, based on larger datasets, we assume that there is a high potential in neural network exploration.*

*In addition to the discussion about future potential of NNs on larger similar datasets, we have added a sentence to the manuscript to mention this analysis (Machine learning models section in Methods):*

*“ Besides the scikit-learn grid search we explored the potential of more complex and deeper neural networks on the generic dataset (dropout regularization; up to 5 hidden layers), however, we could not find a configuration that outperforms the best Random Forest setting. ”*

2. The experimental observations are restricted to ENCODE data which may be biased as certain protocols were applied in ENCODE to annotate data quality. Thus, I am curious about the performance of models trained on ENCODE and tested on data sets other than ENCODE (e.g. GTEx etc.). I understand that there may be no comparable data sets as big as ENCODE, but a test on a few hundreds good and bad files should be sufficient to show the generalization capacity of the methods. The last section (Application to independent diagnostic studies) in the current version is insufficient to convince readers about generalization.

*We have performed an additional external validation using 610 human and mouse files from 32 datasets not used for training (not in ENCODE) and indexed in the Cistrome database (217 ChIP-seq, 220 DNA-seq, and also 173 ATAC-seq files). We have added a table listing the datasets (Supplementary Table 4). We used the optimal generic model trained on the full ENCODE data to derive low-quality probabilities of the Cistrome files and compared with number of automatically computed bad-quality flags provided by Cistrome (Figure 7 and S13). High positive correlations show the generalization of this model to the independent samples across different subsets. Results for optimal specialized models showed also positive correlations but mostly lower and with more variance. Although we could not compare to a binary categorization of high- and low- quality such as with ENCODE, we can clearly observe the following relation: the higher the low-quality probability, the lower the quality according to Cistrome’s guidelines. A new section of the manuscript details this additional validation (see section “Testing models generalizability on independent datasets”).*

*Moreover, we have updated the validation on independent RNA-seq samples to 6 GEO datasets for a total of 90 samples. We compared the clustering of the samples by group (control or disease) on a PCA before and after removal of predicted outlier samples, arbitrarily defined as the 2 samples per group with highest low-quality probabilities (optimal generic model). Using the Dunn’s index to evaluate the quality of the clustering (the higher the better), the removal of outliers led to improvements in 4 datasets and was not detrimental otherwise (Figure 6 and S12). This demonstrates the generalization of the model to independent RNA-seq samples and its relevance to identify potential outliers.*

3. Regarding feature selection, why was chi-square used? Since correlations between features can't be considered in univariate feature selection methods, stability selection can be considered as a better choice (https://rss.onlinelibrary.wiley.com/doi/10.1111/j.1467-9868.2010.00740.x).

*We agree that there are further feature selection methods that might improve our approach. We used chi-square feature selection, since simple filter methods are lightweight and the relatively small dimension of our feature space would not necessarily require more sophisticated selection methods. Nevertheless, following the reviewer’s suggestion, we included two other methods in the grid search: the stability selection method as suggested and the recursive feature elimination (RFE) from scikit-learn based on the Support Vector Regressor with a linear kernel. As suggested by the corresponding github description for the stability selection, we used the logistic regression with the liblinear solver and l1 penalty for the stability selection setup.*

*After examining the results from the extended grid search, we observed only in two cases an improvement of 0.001 in auROC when stability selection was applied within the cross-validation. The small impact of this method on the performance may be related to the better suitability of the method to data of higher dimensionality. For both the chi-square and RFE methods, the average improvement was 0.005 of auROC, which is also very low. However, at most, the predictive performance measured in auROC could be increased by 0.18 and 0.19 by chi-square and RFE, respectively. Because of this, the chi-square and RFE feature selections are precomputed for the usage in the software tool in case the predictive performance could be improved by one of the two methods. The text in the manuscript was changed according to these extensions and the corresponding plot (Fig. 4C) was updated.*

MINOR:

2,642human -> 2,642 human

*Thanks for pointing out this typo. We have fixed it.*

REVIEWER #2:

Assessing the quality of sequencing data is a critical task. Many methods and metrics have been proposed to tackle this task. The authors developed an automated pipeline that can predict the quality from the raw data. They have analyzed 2642 human and mouse raw sequence data annotated by the ENCODE consortium using 10 machine learning methods with a grid search of parameters. They assessed the quality prediction of individual features and multiple features. This study provides a comprehensive benchmark of features and models to predict ChIP-seq, DNase-seq and RNA-seq data quality.

MAJOR

1. The computational procedure needs to be better explained, such as which machine learning is used for prediction (see below).

*We thank the reviewer for the valuable comments and ideas. We have made multiple changes to the manuscript in order to clarify the usage of machine learning methods. We have clarified the difference made between models tuned by the grid search and optimal models (best performing tuned model by data subset). We have also included 2 tables to provide exact parameters used to train those models (Supplementary Tables 2 and 3), and a table detailing all tested parameter values in the grid search (Supplementary Table 1)*

2. ENCODE DCC provided guideline for each data set with recommended sequencing depth and uniquely mapped reads. It would be useful to discuss these features and how useful they are for predicting data quality. It'd be helpful to improve the guideline.

*The ENCODE guidelines define minimal amounts of aligned reads, uniquely mapped reads or usable fragments depending on the data type. The current guidelines define 30 million aligned reads for RNA-Seq, 20 uniquely mapping reads for DNase-seq, 20 million usable fragments for narrow-peak Histone and TF ChIP-seq, 45 million usable fragments for broad-peak Histone ChIP-seq, and 45 million total mapped reads for H3K9me3 ChIP-seq. Importantly, the ENCODE guidelines have a broader scope as they also detail other important points related for example to types of replicates, antibodies or annotations.*

*Nevertheless, we have compared the ENCODE data files used to train the models with the guidelines (panels A, C and E on Supplementary Figure S14). We observed that the minimum value defined by the guideline matches actually the observed median of released ENCODE files in DNase-seq, narrow-peak ChIP-seq, and H3K9me3 ChIP-seq. Although approximately 75% of the released RNA-seq files are above the guideline, the revoked RNA-seq files are also above the value but with a higher variance. Finally, more than 75% of the broad-peak ChIP-seq files do not reach the minimal value, for both released and revoked files. Consequently, the ENCODE DCC recommendations on sequencing depth, mapped reads and usable fragments can not be used to classify the data by quality.*

*Together with the larger content of the guidelines, our observations illustrate the complexity of the quality control procedure in ENCODE that can not be summarized by a single or a pair of features. Due to its good predictive performance, our software reproduces comparable results (panels B, D and F on Supplementary Figure S14). We have added more details to the manuscript together with more information about revoked data:*

*In the Methods:*

*“In ENCODE, the status of whole experimental datasets or single files is changed to revoked if they were deemed erroneous or significantly below standards after release. We used this status as an indication of the quality of a file and considered FastQ files to be low quality when revoked and high-quality when released.”*

*In the Results (first paragraph):*

*“Interestingly, minimum number of uniquely mapped reads and usable fragments mentioned in the ENCODE guidelines cannot be used to categorize the ENCODE data with respect to quality (Figure S14 A, C and E).”*

3. Related to the above point, the authors should summarize the analysis and recommend a generalized model with specific algorithm and parameters for quality prediction that can be applied in practice. This is what readers most want to know but it is lacked in the manuscript.

*We agree that this was not clearly communicated in the manuscript. We refer to this model as the optimal generic model. It was trained on all samples and all features contrary to optimal specialized models that were trained on data specific to a species and an assay type. It is based on Random Forest with 1000 estimators (see Supplementary Table 3 for all parameters). It generalizes very well to various types of data in the independent validation (Figure 6, 7, S12 and S13), it is well calibrated (Figure S8), and it is not biased by data subset (Figure 5B), library layout (Figure S5B) or inclusion of quality features related to reads localization (LOC and TSS features; Figure S11). It also performs as good as the best specialized models (Figure 5B). The generalized model is the default in the software. It is described and discussed in different sections of the manuscript such as in the discussion:*

*“This model also generalized better to external datasets, including human and mouse RNA-seq, ChIP-seq, DNase-Seq and even ATAC-Seq data, a type of assay that had not been used in the ENCODE training set.”*

*“Given its good performance and generalization on independent samples, the optimal generic model is the default model in the software.”*

4. ChIP-seq data for narrow and broad peaks can be very different in terms of quality assessment. It is critical to separately evaluate broad peak ChIP-seq data and check which features are predictive.

*In order to better characterize broad and narrow peak ChIP-seq data, we created subsets of single-end ChIP-seq data for human and mouse. These subsets were also balanced with respect to the quality status. The single-end data was used because the broad peak samples are extremely underrepresented in the paired-end ChIP-seq subset.*

*We performed the one-feature performance analysis as it was done in Figure 2A of the main manuscript (Supplementary Figure S9). Results show the one-feature auROC for the standard ChIP-seq and the two peak-type-specific datasets. Clear differences can be observed between narrow and broad peaks. We explain the larger differences of broad peak data across species by difference between the samples in term of sample types (Supplementary Figure S7). The following sentences have been included in section One-feature quality predictions as baseline and guidelines:*

*“Comparing species, DNase-seq showed more differences than ChIP-seq results. This is probably due to the large difference in composition between human and mouse DNase-seq samples (Supplementary Figure S7). Analyzing separately narrow and broad peak samples from the ChIP-seq results, we could observe an under-representation of broad peak samples (only 82 human and 46 mouse samples) and differences between peak types (Supplementary Figure S9).”*

*In order to analyze if this has an impact for machine learning classification we applied the grid search to obtain peak-type-specialized models (Supplementary Figure S10). We compared them to the specialized models for ChIP-seq (either human or mouse ChIP-seq samples) and generic models (all samples). When using all features on the narrow-peak data, all models performed similarly. When using all features on the broad-peak data, all models were close for mouse but the peak-type-specialized model performed better for human.*

*The broad peak models for mouse data were trained on rather small datasets, which decreases the reliability of the model. For human broad peak ChIP the data is also not large; however, we see an improvement over the ChIP-seq model. Because of the latter observation we decided to include an optional parameter to the software that allows the specification for broad or narrow peak input data. The following was added to the manuscript in section Multi-feature quality predictions by machine learning:*

*“Nevertheless, we also evaluated separate models for narrow-peak and broad-peak ChIP-seq samples (Supplementary Figure S10). Considering all features, human and mouse narrow-peak models performed similarly to the generic and less specialized ChIP-seq models described above. Broad-peak models outperformed the other models although we could not exclude a bias due to the lower sample size of the underlying training sets.”*

5. Two important data types, ATAC-seq and DNA methylome, are not included in the analysis. For a comprehensive analysis study, they should be included.

*There were no revoked files in ENCODE for these assay types, that would enable us to build and test models. Support of DNA methylome data is planned for a future update, however, we could show in our external validation that human and mouse ATAC-Seq data can be correctly predicted with our optimal generic model and possibly better with the optimal specialized model for DNase-seq (Figure 7 and S13).*

*Thanks to an arbitrary binary categorization of the Cistrome samples based on the number of automatically cpmputed quality flags (bad quality if 3 bad-quality flags or more, high quality otherwise), we were able to test a model trained directly on the Cistrome ATAC-seq data. It showed high performance in 10-fold cross-validations (auROC = 0.91; no independent validation) and was included in our software to enable users to apply it to their own ATAC-Seq data.*

*This is now described in the Results section as follows:*

*“On the ATAC-Seq data, the generic model also produced probabilities that positively correlated with the flags. However, the correlation was high with mouse data (0.61) and only moderate with human data (0.15). Applying the specialized DNase-seq models to ATAC-seq data also worked with mouse data (0.44) and improved the results for human data (0.46). Although this is not an external or independent validation, we note that a Random Forest model directly trained on ATAC-seq data showed high performance in 10-fold cross-validations (auROC of 0.910 and auPRC of 0.932; see Methods).”*

MINOR ISSUES:

1. It is unclear how the one-feature quality prediction was performed. How the AUCs reported in Fig. 2 are obtained is not described. It seems no machine learning model is used. Is it only enrichment of each feature shown in Fig. S1 used to predict data quality?

*The reviewer is right. No machine learning is involved and we use it as baseline for evaluating the machine learning models in figure 2B. The following paragraph has been added to the manuscript for clarification:*

*“The one-feature ROC-curves were derived by using the feature values as probabilities describing a non-machine learning probabilistic model. For example, the higher the uniquely mapped reads rate, the lower the probability of having a low-quality sample. A further example is the no mapping rate. In this case the higher these values, the higher the probability of having a low-quality sample. Hence, the features need to be treated differently. Therefore, we derived two ROC curves using first the default quality labels and second the inverted labels. Finally, the maximum area under the curve for a given feature was reported.”*

2. For one-feature prediction, human and mouse DNAse-seq diverge more than the ChIP-seq data, particularly for the MAP and TSS features. Any reason for that?

*We hypothesize that it could be related to the difference in sample types included in each DNAse-seq subset. We have added Supplementary Figure S7 to show those differences and as mentioned above the following sentence in the Results section:*

*“Comparing species, DNase-seq showed more differences than ChIP-seq results. This is probably due to the large difference in composition between human and mouse DNase-seq samples (Supplementary Figure S7).”*

3. For multi-feature prediction in Fig. 2B, it is also unclear which machine learning is used and how the AUC was obtained.

*In Figure 2B, the one-feature prediction values are the best performing auROC per feature set. The machine learning values were derived from the best performing specialized model of the complete parameter space in a gridsearch. For a given data subset, it was trained with all features from that set and with all four sets for ALL. The auROC values are the mean auROC of a 10-Fold cross validation. We have updated the following sentence in the legend of Figure 2 for clarification about AUC and used models:*

*“Predictive performances of optimal generic and specialized machine learning models trained using given feature sets (y-axis) outperform one-feature predictions. Bars show mean value and error bars show standard deviations derived from 10-fold cross-validations within the grid search.”*

4. The same problem for Fig. 3, it is unclear which models are used to calculate the probability.

*For this analysis we always used the best specialized model, hence, the model that achieved the best auROC based on the corresponding dataset specification (species, assay, run-type). We expanded the figure description in order to clarify this point as follows:*

*“The low-quality probabilities Plow shown in panels B, C, and D were computed by the optimal specialized models trained on the corresponding data subset but without the files from the experiment under investigation”*

5. Fig. 1B, many data types have less than 200 files. It'd be useful to put the file number next to the bars to indicate how many files are available.

*We have updated the figure accordingly.*

REVIEWER #3:

The authors described a machine learning-based sequencing data QC tool. The method uses input from fastQC, Bowtie2 alignment statistics, and extra location distribution features and is trained on the released vs revoked data from ENCODE. This is an interesting approach that is potentially widely applicable. The machine learning approach could allow inexperienced user to learn from expert decisions and established a decision boundary for low quality sample. However, more evaluations and potential improvement are needed to demonstrate these potentials are practical, especially on external datasets not used in model training, as detailed below.

MAJOR POINTS:

1. External validation - a major concern for this study is that it heavily relies on training and validating on a specific dataset that may contain certain biases. The study on external datasets is small-scale and inadequate for demonstrating its performance on external data with confidence. A more extensive study on external datasets is needed (e.g. on Roadmap Epigenomics if it is not overlapping with the training data). Even though hand curated dataset quality labels are generally not available, the majority of the published should have passed quality control. The model predictions should reflect that and the datasets classified as the lowest quality may be manually inspected for their quality issues.

*As detailed above for Reviewer 1, we have performed an additional external validation using 610 human and mouse files from 32 datasets not used for training (not in ENCODE) and indexed in the Cistrome database (217 ChIP-seq, 220 DNA-seq, and also 173 ATAC-seq files). We have added a table listing the datasets (Supplementary Table 4). We used the optimal generic model trained on the full ENCODE data to derive low-quality probabilities of the Cistrome files and compared with number of automatically computed bad-quality flags provided by Cistrome (Figure 7 and S13). High positive correlations show the generalization of this model to the independent samples across different subsets. Results for optimal specialized models showed also positive correlations but mostly lower and with more variance. Although we could not compare to a binary categorization of high- and low- quality such as with ENCODE, we can clearly observe the following relation: the higher the low-quality probability, the lower the quality according to Cistrome’s guidelines. A new section of the manuscript details this additional validation (see section “Testing models generalizability on independent datasets”).*

*Moreover, we have updated the validation on independent RNA-seq samples to 6 GEO datasets for a total of 90 samples. We compared the clustering of the samples by group (control or disease) on a PCA before and after removal of predicted outlier samples, arbitrarily defined as the 2 samples per group with highest low-quality probabilities (optimal generic model). Using the Dunn’s index to evaluate the quality of the clustering (the higher the better), the removal of outliers led to improvements in 4 datasets and was not detrimental otherwise (Figure 6 and S12). This demonstrates the generalization of the model to independent RNA-seq samples and its relevance to identify potential outliers.*

2. Evaluation. It is unknown whether the predicted probabilities are well calibrated (i.e. are 90% positive label observed when prediction is 0.9 on training data)? This is important as a user will need to rely on the score provided by the software to make QC decision with necessarily any reference to compare with. The method's performance with different thresholds or a recommended threshold (TPR, FPR, Precision) should also be presented to guide the usage.

The authors mentioned that the predicted probabilities may not be comparable across models and studies. I appreciate their honesty in bringing this up, and I think this is an important issue that need to be addressed as it otherwise greatly reduced the usefulness of this software. If the probabilities are not comparable across models, the model output may be calibrated with isotonic regression or logistic monotonic spline regression on the training data. If the probabilities are not comparable across studies, then it needs to be addressed how the user should interpret the results, without relying on the users training their own models which is not realistic most of the time.

*We appreciate that the reviewer raised the issue of calibration and decision making. We performed the grid search again and calculated the Brier loss metric for each algorithm-parameter setting combination. The Brier loss describes how the predicted probabilities are calibrated and allows us to assess the trained models with respect to this aspect. We then analyzed two extreme selections for the best model, one only by highest auROC ignoring the Brier loss and another one only by lowest Brier loss ignoring the auROC. Supplementary Figure S8 shows the auROC and Brier loss for these two models for different datasets and feature set scenarios. In most of the cases the lowest Brier loss is not much lower than the Brier loss achieved by the model with the highest auROC. In some cases an improvement could be observed, however in such cases the Brier loss is usually already low (RNA-seq and DNase-seq). Another observation is that it is beneficial to use all feature sets together as the models trained with all features achieved the lowest Brier loss values. In general, the Brier loss metric suggests that the probabilities are well calibrated in case all feature sets are used. This is now mentioned in section Best performing model selection.*

*We also investigated the potential of calibration methods from the scikit-learn package to calibrate the probabilities of the best performing classifier. However, we could not observe an improvement for the default calibrator neither for the isotonic nor for the sigmoid method. Thus, on our datasets, changes with respect to the calibration are rather caused by the different classifiers than by a calibration method. Because of this we decided to extend our software by an optional user-defined parameter that allows to use the best algorithm-parameter setting selected by the lowest Brier score.*

*Moreover, in order to simplify the decision making for the user, we extended the implementation of the software to display more information to the user. This additional information describes the cross-validated Precision, Recall, F1, and accuracy for different decision thresholds. From this information the user can select the best decision threshold when using the tool on new data.*

*The usability of the approach on independent data is thoroughly investigated by the additional analysis on large independent datasets from GEO and CistromeDB (Figure 6, 7, S12 and S13).*

*We also included the following explanation for comparisons across studies in the Discussion:*

*“Yet, we could expect that a technical batch effect would prevent the comparison of the results of the models between datasets, especially with RNA-seq datasets. In such a case, it would still be possible to compare probabilities within a batch, but the comparison between batches could be done using ranks of probabilities.”*

3. Features. Training on ENCODE released vs revoked data is an interesting idea. As these labels are manual in nature, and the ENCODE data generators / curators most likely used established QC metrics and tools in making these decisions, the machine learning method should recapitulate these decisions as closely as possible. Therefore, the machine learning approach should have a comprehensive coverage of existing QC metrics in its input features. Even though the authors included a set of clearly informative features, including FastQC metrics, it is unclear whether this set of features covers metrics used on data type-specific QC tools for RNA-seq (e.g. RSeqQC ) and ChIP-seq / DNase-seq QC( e.g. ChiLin).

Another feature-related concern is the inclusion of LOC and TSS features for ChIP-seq. As ChIP-seq for different targets are expected to have highly distinct location distributions, would inclusion of these features bias against / toward particular types of ChIP-seq targets?

*We agree that the machine learning method should recapitulate the QC decisions as closely as possible. For this purpose, we demonstrate the performance of the models throughout the manuscript, especially in Figure 3C showing results within experiments, and in Supplementary Figure S14 showing recapitulation of the sample quality in comparison to ENCODE guidelines. To make the models applicable across various types of NGS data files and to keep preprocessing times low, we have selected a limited number of complementary features that are broad in scope. Yet, some features partially overlap with the output of data-type-specific QC tools such as RSeqQC (e.g. read quality or read distribution). The independent validation on Cistrome datasets and related guidelines demonstrates the agreement of our models with Cistrome QC guidelines using assay-specific features such as the Fraction of Reads in Peaks (FRiP score) or the union DHS (DNase I hypersensitive site) overlap, which uses peak information not used by the models (Figure S7 and S13 and section External validation on datasets from Cistrome).*

*The number of features will certainly be extended in the future after careful evaluation of potential bias that may occur, such as described by the reviewer for LOC and TSS features. To investigate a potential bias with those sets of features, we have plotted the distribution of probabilities returned by the software for various protein targets in ChIP-seq experiments. This was done for the optimal generic model either using all features or using RAW and MAP features only (Supplementary Figure S11). In general, we could observe no bias against or toward particular types of ChIP-seq targets after inclusion of LOC and TSS features. Together with the higher performance of models using all features (figure 2B), we conclude that LOC and TSS features are valuable and do not bias the models.*

*This is now mentioned in the manuscript for example in the Results (section Generic model):*

*“We compared the predictions across different ChIP-seq protein targets; we were unable to observe any bias towards particular targets or an effect of including LOC and TSS features in the model (Supplementary Figure S11). Moreover, we could observe consistency of the predictions with the real annotations when observing distributions of uniquely mapped reads and usable fragments in relation to the ENCODE guidelines (Supplementary Figure S14 A-F).”*

MINOR:

1. Is there any source / references that explain the reasons for the revocation status of ENCODE data and that the reason for revocation is mostly due to low-quality?

*There are descriptions about the file status within the ENCODE documentation under “Status Terminology”:*

*https://www.encodeproject.org/help/getting-started/status-terms/#FileStatuses*

*The two status “revoked” and “archived” are essentially assigned to files that were at some point “released”. According to the descriptions, there is a key difference that files are mostly archived when data is outdated or superfluous whereas the revoked status is used when “This file was deemed erroneous or significantly below standards after it had been released”. From this we conclude that revoked files are consequently from much lower quality than “released” files.*

*We updated the corresponding paragraph in the manuscript to provide more details from this description:*

*“In ENCODE, the status of whole experimental datasets or single files is changed to revoked if they were deemed erroneous or significantly below standards after release. We used this status as an indication of the quality of a file and considered FastQ files to be low quality when revoked and high-quality when released. ”*

2. Machine learning parameters. The number of parameters were mentioned in the text but no specific descriptions about what parameters were tried are provided.

*We have added 3 tables to the supplementary material describing all parameters used within the grid search and also the best performing algorithm parameter setting for the different datasets (Supplementary Tables 1, 2 and 3).*

**Second round of review**

**Reviewer 1**

My previous three concerns have been properly addressed in this revision manuscript. I have no further comments.

**Reviewer 2**

The authors have addressed my comments.

**Reviewer 3**

I thank the authors for the revision. They have sufficiently addressed my points.
